# Orthogonal, modular anion–cation and cation–anion self-assembly using pre-programmed anion binding sites†

**DOI:** 10.1039/d2sc05121d

**Published:** 2023-02-03

**Authors:** Ayan Dhara, Rachel E. Fadler, Yusheng Chen, Laura A. Köttner, David Van Craen, Veronica Carta, Amar H. Flood

**Affiliations:** a Department of Chemistry, Indiana University 800 East Kirkwood Avenue Bloomington IN 47405 USA aflood@indiana.edu; b Department of Chemistry and Biochemistry, University of Windsor Windsor Ontario N9B 3P4 Canada; c Wayne State University Law School, Wayne State University 471 W Palmer Ave Detroit MI 48202 USA; d Department of Chemistry and Pharmacy, Friedrich-Alexander-Universität Erlangen-Nürnberg Nikolaus-Fiebiger-Str. 10 91058 Erlangen Germany; e Department of Chemistry and Chemical Biology, Technische Universität Dortmund Otto-Hahn-Str. 6 44227 Dortmund Germany

## Abstract

Subcomponent self-assembly relies on cation coordination whereas the roles of anions often only emerge during the assembly process. When sites for anions are instead pre-programmed, they have the potential to be used as orthogonal elements to build up structure in a predictable and modular way. We explore this idea by combining cation (M^+^) and anion (X^−^) binding sites together and show the orthogonal and modular build up of structure in a multi-ion assembly. Cation binding is based on a ligand (L) made by subcomponent metal-imine chemistry (M^+^ = Cu^+^, Au^+^) while the site for anion binding (X^−^ = BF_4_^−^, ClO_4_^−^) derives from the inner cavity of cyanostar (CS) macrocycles. The two sites are connected by imine condensation between a pyridyl-aldehyde and an aniline-modified cyanostar. The target assembly [LM-CS-X-CS-ML],^+^ generates two terminal metal complexation sites (LM and ML) with one central anion-bridging site (X) defined by cyanostar dimerization. We showcase modular assembly by isolating intermediates when the primary structure-directing ions are paired with weakly coordinating counter ions. Cation-directed (Cu^+^) or anion-bridged (BF_4_^−^) intermediates can be isolated along either cation–anion or anion–cation pathways. Different products can also be prepared in a modular way using Au^+^ and ClO_4_^−^. This is also the first use of gold(i) in subcomponent self-assembly. Pre-programmed cation and anion binding sites combine with judicious selection of spectator ions to provide modular noncovalent syntheses of multi-component architectures.

## Abstract 2

Use of pre-programmed anion binding sites provide structure-directing elements in subcomponent self-assembly that operate in an orthogonal way to the traditional role of cations.

## Graphical abstract

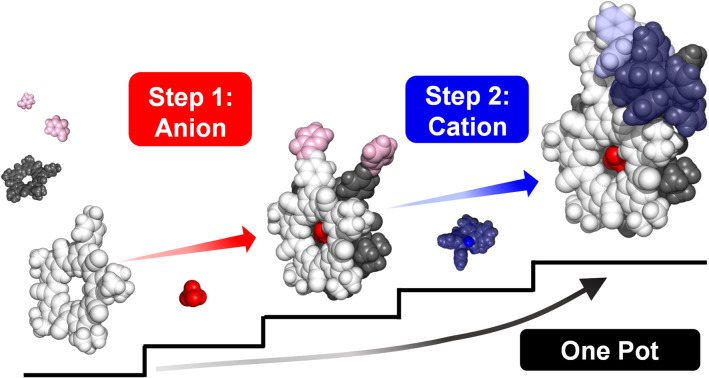

## Introduction

**Fig. 1:**
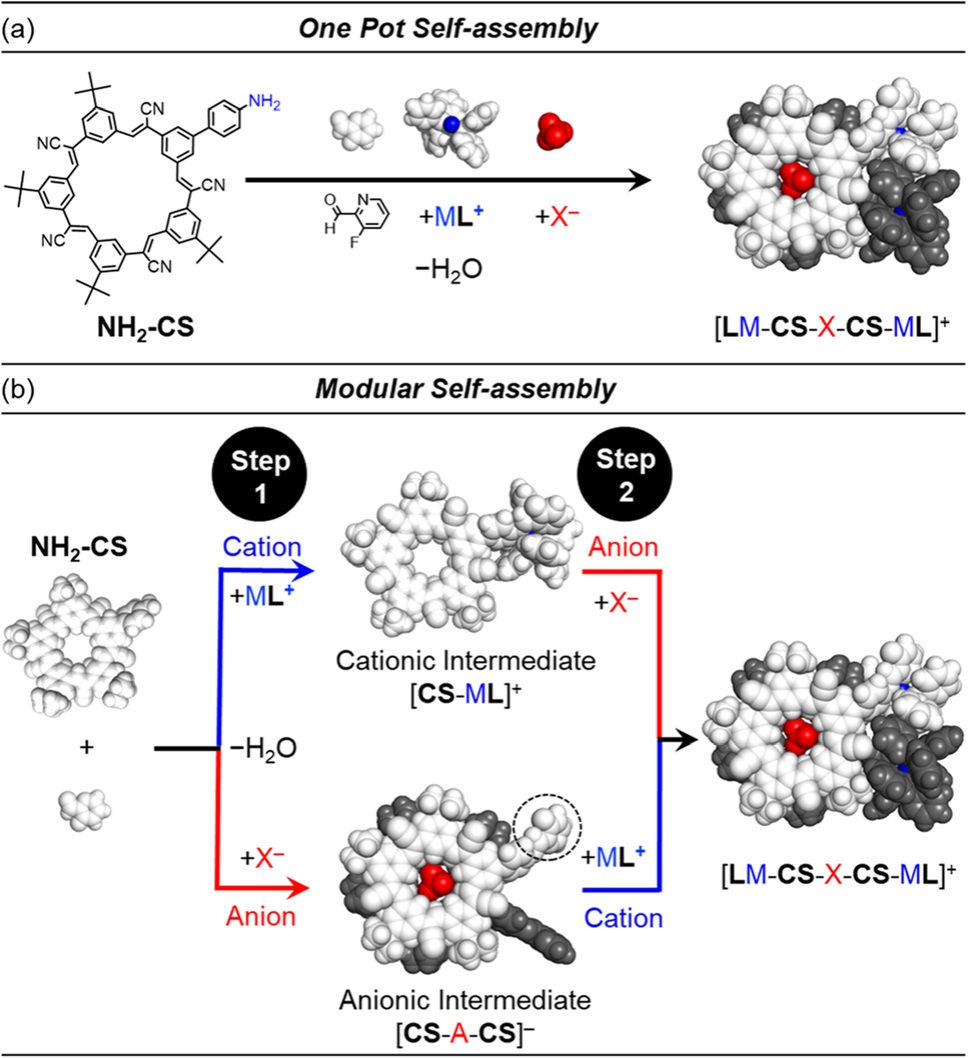
Schematic summary of the (a) one-pot and (b) two-step self-assembly pathways that use pre-programmed and orthogonal coordination of cations and anions. In the resulting assembly (at right) the top cyanostar is shown in white and the bottom one is shown in dark grey for clarity. The Cu^+^ cation is shown in blue, and the BF_4_^−^ anion shown in red. The pyridyl-based aldehyde is highlighted by the dotted circle in the anionic intermediate.

The role of anions in cation-directed self-assembly is growing in importance. They act as templates^1–3^ for coordination cages,^4^ helicates^5^ and knots,^6^ triggers for switching between structures,^1,7,8^ they solubilize^9^ the final assembly in desired solvents, and get encapsulated as guests.^10^ These many roles have grown on the back of metal-directed subcomponent self-assembly^11^ involving the elegantly direct and *in situ* Schiff-base condensation of modular ligands. Across all these roles, the anion's binding site is built up dynamically by the cation-directed self-assembly^12–15^ where metal ion coordination remains the primary structure-directing interaction. The anions have only rarely been explored as orthogonal motifs for building up structural features in a manner that matches metal ions. A notable exception^16^ from Jansone-Popova is the synergistic one-pot self-assembly of a helicate around divalent metal cations, (Cu^2+^) and sulfate anions (SO_4_^2−^) all directed by heteroditopic ligands composed of separate cation and anion binding sites. Herein, we investigate the orthogonality between anion coordination chemistry^17,18^ and cation coordination for the modular build-up of ion-driven assemblies (Fig. 1).

The realization of structure-directing anion coordination is enabled by advances in receptor design.^19–23^ Examples include anion helicates^24^ with ditopic ligands bearing terminal ureas wrapped around phosphate (PO_4_^3−^).^25^ Cages,^26^*e.g.*, tetrahedra, have combined urea motifs with highly charged phosphates in the vertices.^27^ Polymers have been developed, *e.g.*, the Texas-sized box electrostatically stabilizes the polymerization of terephthalates,^28^ and cyanostar stabilizes polymerization of diphosphonates^29^ or diphosphates.^30^ The counter cations in these cases, once again, play secondary roles.

Examples of multi-ion assemblies with anions and cations serving as equal partners are under-represented.^31–35^ Ion-pair receptors can bind cations, anions and ion pairs but such receptors are not used to build up structure unless both ions^36^ bind at the same time to pre-formed metal and anion binding sites.^37^ The most powerful among them afford cooperativity^38–40^ with selective capture of ion pairs over the separate ions.^16^ Such systems rely on one-pot processes with mutual binding of anion and cation without the isolation of intermediates for modular assembly.

Orthogonality between metal coordination and anion recognition offers advantages for self-assembly. When cation and anion do not interact either with each other or the other's binding sites then the assembly should be pre-programmable in a predictable way. Orthogonality also lends modularity. Thus, swapping one type of anion for another alters the composition but not the structural features of the final assembly. These characteristics of predictability and modularity can potentially be demonstrated by separating a one-pot multi-component self-assembly (Fig. 1a) into discrete steps (Fig. 1b).

**Fig. 2:**
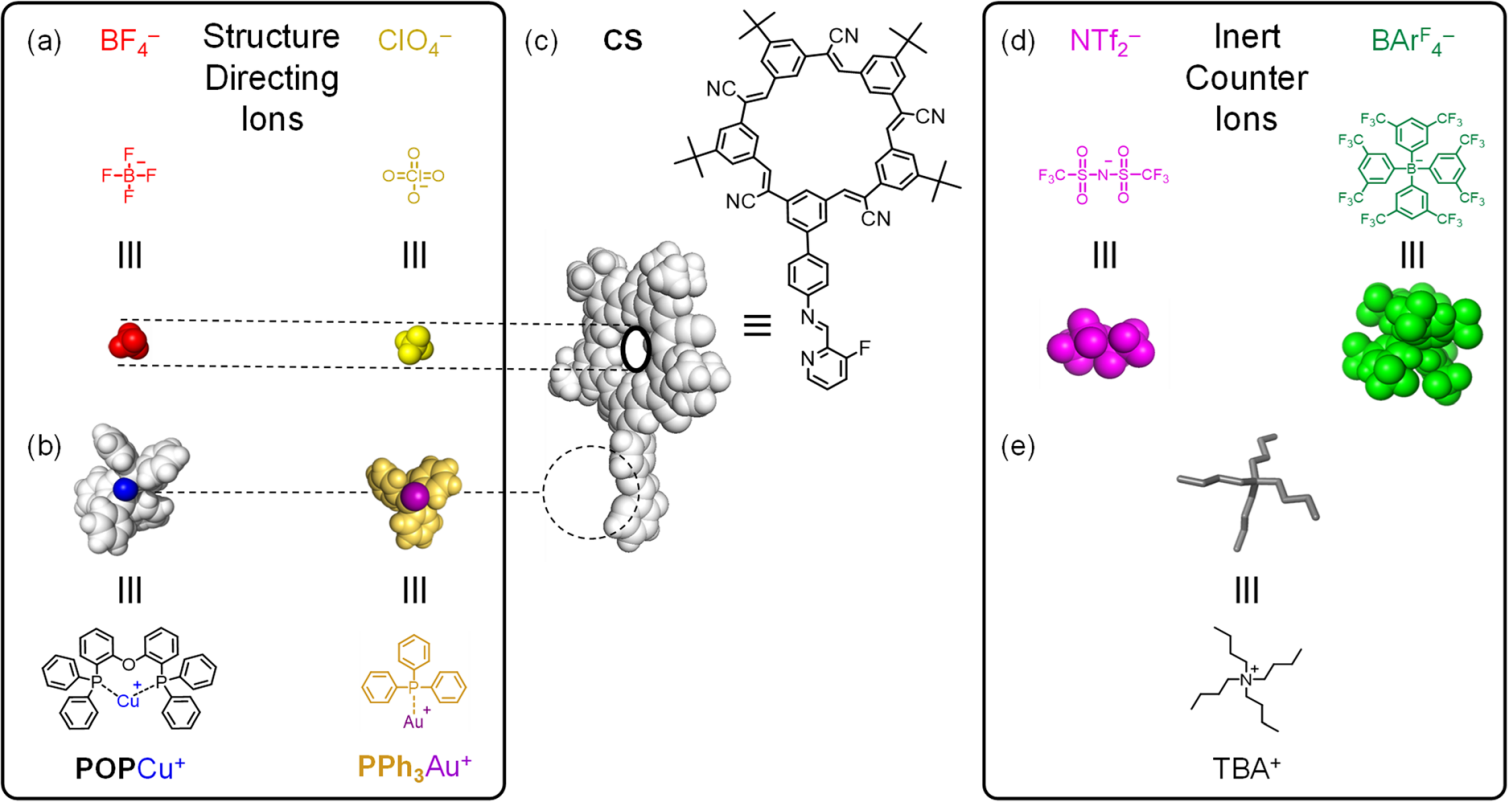
Multi-component ion-directed self-assembly makes use of (a) structure-directing anions and (b) cations, (c) the pre-programmed anion-binding ligand, cyanostar (CS), as well as (d) inert counter anions and (e) counter cations. The Cu^+^ is blue, and the Au^+^ cation is dark purple. Their complexes are distinguishable by their ligands where POP is white and PPh_3_ is gold. Anions are color-coordinated where BF_4_^−^ is red, ClO_4_^−^ is yellow NTf_2_^−^ is purple, and BArF_4_^−^ is green.

Herein, we explore the orthogonality and modularity of ion-driven assembly by combining cation-directed subcomponent self-assembly between imines and metal moieties^42^ (ML; L = POP, PPh_3_; Fig. 2b) with pre-programmed anion binding sites (Fig. 2).^41^ We use cyanostar macrocycles (CS, Fig. 2c)^22^ for strong anion coordination (X^−^, Fig. 2a). Structure-directing cations (M = Cu^+^, Au^+^; Fig. 2b) and anions (X^−^ = BF_4_^−^, ClO_4_^−^; Fig. 2a) can be added to form target assemblies in one-pot confirming the orthogonality of assembly. Modularity is demonstrated by first isolating supramolecular intermediates of either ion (Fig. 1b). The intermediates are accessed by using non-coordinating counterions (Fig. 2d and e). The cationic intermediate [LCu-CS]^+^, and the anionic intermediate [NH_2_CS-BF_4_-NH_2_CS]^−^, can be isolated by trituration and used in subsequent reactions. Exemplary studies with copper(i) and tetrafluoroborate indicate the intermediate can be used as a substrate for a second coordination step to form the final assembly [LCu-CS-BF_4_-CS-CuL],^+^ (Fig. 1b). Modularity was also demonstrated using Au^+^ and ClO_4_^−^ ions. Surprisingly, this is the first example of subcomponent self-assembly^43^ with Au(i). We used other anions (phosphates, phosphonates, bisulfate) to explore the limits of this strategy. Thus, pre-programmed cation and anion binding sites serve as orthogonal interactions that form supramolecular assemblies and help contribute to the growing exploration of methods^44,45^ capable of orthogonal, modular noncovalent synthesis.

## Results and discussions

### Design of the components used in cation–anion self-assembly

In order to demonstrate orthogonality, we needed to simplify the number of potential products that form. We used ancillary ligands on the metals to block the possibility of cross linking. We substituted just one of the five cyanostar arms (Fig. 1a) to enable coordination of one metal ion moiety to the macrocycle as opposed to five in the *C*_5_-symmetric cyanostar. The only drawback to mono-substitution is the presence of overlapping peaks in the NMR spectra. These ligand and receptor features all favor formation of fewer species.

We used knowledge of the recognition patterns of the binding sites to design the various components.^42^ The mono-substituted cyanostar^46^ (NH_2_-CS, Fig. 1) contains a 4-aminophenyl group. We expect this aniline to enable imine formation upon reaction with aldehydes in the presence of metal ions.^43^ We also anticipate anion coordination with NH_2_-CS will match the behavior of parent cyanostar (pCS)^22^ to direct formation of a 2 : 1 sandwich complex with a bridging anion, X^−^. For metal complexation, we selected a fluoro-substituted picolinaldehyde, F-PyCHO, as the partner to the NH_2_-CS aniline for subcomponent assembly.

We identified two metal precursors to explore modularity. The copper(i) complex of bis(2-(diphenylphosphino)phenyl)ether (POP) as the bis-acetonitrile solvato species, {POPCu(MeCN)_2_}^+^, was selected as the first. The POP ligand is known to terminate the assembly after imine coordination^42^ and the acetonitrile is labile. The second is a gold(i) triphenylphosphine moiety, {PPh_3_Au}^+^. Gold(i) has never been used in subcomponent self-assembly and it has unique coordination geometries.^47^ Gold(i) is typically two coordinate,^48^ but three has also been observed when using σ-donors of comparable strength.^49^ The imine nitrogen is a relatively strong donor,^50^ therefore a two-coordinate geometry is possible. Alternatively, the chelating imine is similar to phenanthroline that can favor three-coordinate geometries with gold(i).^51^ A model gold(i) complex was examined to confirm the preferred mode of coordination prior to the cation–anion self-assembly.

### Strategies for orthogonal cation–anion self-assembly

Our strategy for demonstrating the principle of cation and anion self-assembly relies on two design principles: orthogonal binding sites and weak ion-pair interactions. The cyanostar component has an anion-binding site native to its cavity and one cation binding site added to its exterior. Based on their preferred reactivity,^22,42^ these sites do not compete with each other. The second criterion requires knowledge of ion coordination and ion pairing. Copper(i) ions are known to coordinate with free imines while BF_4_^−^ is complementary to the cyanostar. These ions (Cu^+^ and BF_4_^−^) do not display strong interactions with each other in the solvents we use here.^52^ While BF_4_^−^ anions are considered weakly coordinating to metals, they display some of the strongest binding affinities to cyanostar.^22^ But not all anions that bind tightly to cyanostar are inert to metal coordination (see below). These design principles are anticipated to be transferrable to other pairs of ions. To this end, we investigated gold(i), Au^+^, and perchlorate, ClO_4_^−^ to verify the generality of this assembly strategy.

Finally, isolation of intermediates requires selection of non-binding counter ions. Their use enables structure-directing ions to be delivered separately and sequentially. Bulky anions tetrakis(3,5-bis(trifluoromethyl)phenyl)borate (BAr^F^_4_^−^, Fig. 2d) and triflimide (NTf_2_^−^, Fig. 2d) fulfill the requirements for weak counter anions. They are well-established to coordinate poorly to both metal ions^53,54^ and cyanostar.^55^ NTf_2_^−^ has a low affinity (250 ± 30 M^−1^) for cyanostar.^52^ Similarly, the TBA^+^ counter cation (tetrabutylammonium, Fig. 2e) is not known to coordinate to metal-binding ligands. While the ion pairing of TBA^+^ in dichloromethane with our target anions, *e.g.* BF_4_^−^ log *K* = 4.6 (ref. 56) and ClO_4_^−^ log *K* = 4.24 (ref. 57) is not negligible, they are weaker than the coordination with parent cyanostar, *e.g.*, log *β*(pCS-ClO_4_^−^-pCS) ∼12.^22^ Thus, any ion pairing does not interfere.

### One-pot, multi-component self-assembly using Cu^+^-coordination and cyanostar-BF_4_^−^ binding

**Fig. 3:**
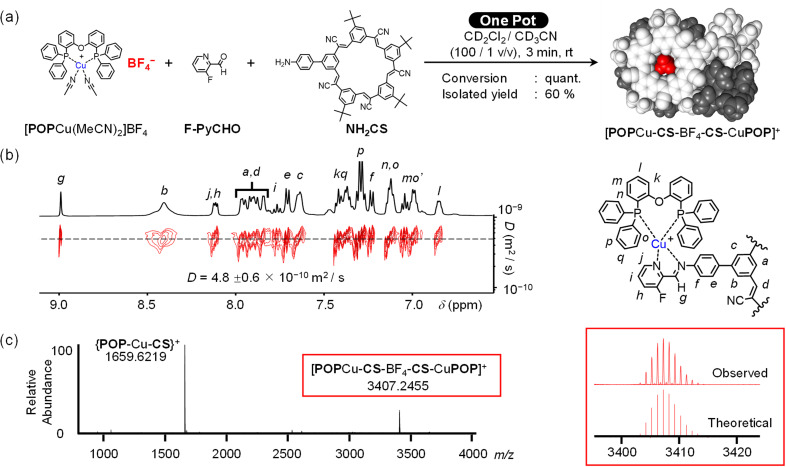
(a) One-pot reaction to form [POPCu-CS-BF_4_-CS-CuPOP]^+^ and characterization by (b) DOSY NMR (CD_2_Cl_2_, 2 mM), and (c) ESI-MS spectrum of assembly (CH_2_Cl_2_, 2 mM).

Orthogonality between the two binding sites was verified using a traditional one-pot assembly. The structure-directing Cu^+^ and BF_4_^−^ ions can be added as a single precursor (Fig. 3a). The target assembly [POPCu-CS-BF_4_-CS-CuPOP]^+^, is a dicopper and anion-bridged dimer. The copper binding sites are products of imine condensation between NH_2_CS and F-PyCHO in the presence of the salt [POPCu(MeCN)_2_]·BF_4_. Mixtures of the three components proceed near-quantitatively through to the target assembly. The product was isolated as a BF_4_^−^ salt after solvent removal. Exploration of the assembly conditions revealed that excess aldehyde enhanced the yield and unreacted aldehyde could be removed with ether washes. Excess NH_2_CS was not investigated on account of its poorer solubility relative to the aldehyde.

Product identity was confirmed using NMR spectroscopy and electrospray ionization mass spectrometry (ESI-MS). The well-resolved ^1^H NMR peaks (Fig. 3b) indicate high-fidelity Cu(i)-complexation and BF_4_^−^ binding in the target assembly. Diffusion ordered spectroscopy (DOSY, Fig. 3b) verified formation of a single monodisperse species. ESI-MS shows the peak for the parent assembly [POPCu-CS-BF_4_-CS-CuPOP]^+^ at 3407.2455 *m*/*z* (Fig. 3c). Under ESI-MS conditions, minor multimer peaks are observed from 0.5 Dalton peak-to-peak separation associated with a dimer of the target: [POPCu-CS-BF_4_-CS-CuPOP]_2_^2+^. We also see a daughter peak corresponding to the anion-free half of the assembly assigned to the {CS-CuPOP}^+^ moiety at 1659.6219 *m*/*z*.

Imine formation and metal coordination show characteristic changes in the chemical shifts of key protons upon formation of the target assembly (Fig. S53†). The aldehyde (10.16 ppm) is consumed to form the imine (9.01 ppm).^58^ Other proton close to the metal binding site are characteristic of imine bond formation and copper complexation.^42^ The aniline's ring hydrogen (H_f_, 6.84 ppm) shifts downfield (7.23 ppm), and the phosphine protons (H_k_) shift modestly from 7.02 to 7.09 ppm.

**Fig. 4:**
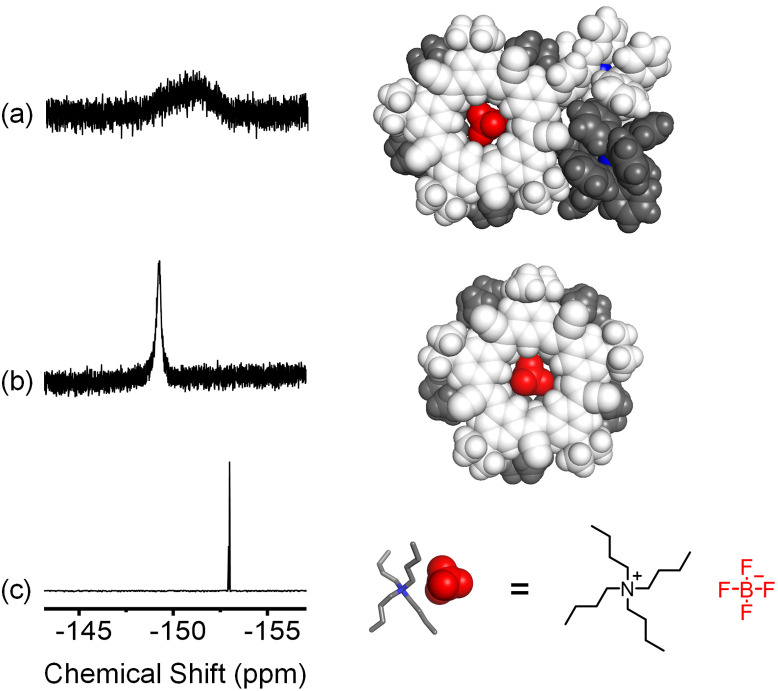
^19^F NMR (a) [POPCu-CS-BF_4_-CS-CuPOP]^+^ created in one-pot, (b) [pCS-BF_4_-pCS]^−^, and (c) TBABF_4_ in CD_2_Cl_2_ (298 K, 376 MHz).

Anion binding in the target assembly is confirmed by ^1^H NMR (Fig. S53†). Peak assignments were based on ROESY studies, controls, and assignments of a related cyanostar.^21^ The cluster of peaks at 8.76 ppm in NH_2_CS are assigned to the outer H_b_ protons of the macrocycle. After one-pot assembly, these shift upfield to 8.41 ppm matching the parent cyanostar.^22^ The ^19^F NMR signal from BF_4_^−^ shows a single broad peak at −150 ppm (Fig. 4a) consistent with exchange averaging between complexed and outer-sphere ions.^59^ The spectrum of BF_4_^−^ as a TBA^+^ salt shows a sharp peak at −152 ppm (Fig. 4c) while the 2 : 1 complex formed between BF_4_^−^ and the parent cyanostar (Fig. 4b) resonates as a sharp peak at −148 ppm.

### Cation-directed supramolecular intermediates

Formation of the desired multi-component assembly [POPCu-CS-BF_4_-CS-CuPOP]^+^, in one pot (Fig. 1a) with high yields provides a basis for exploring stepwise synthetic strategies (Fig. 1b). We separately investigated generation of cation-directed and anion-directed intermediates by relying on their pre-programmed binding sites. In traditional cation-directed assembly, it is not possible to isolate the anion-directed intermediates because the anion binding site is built *in situ* instead of being pre-programmed.

**Fig. 5:**
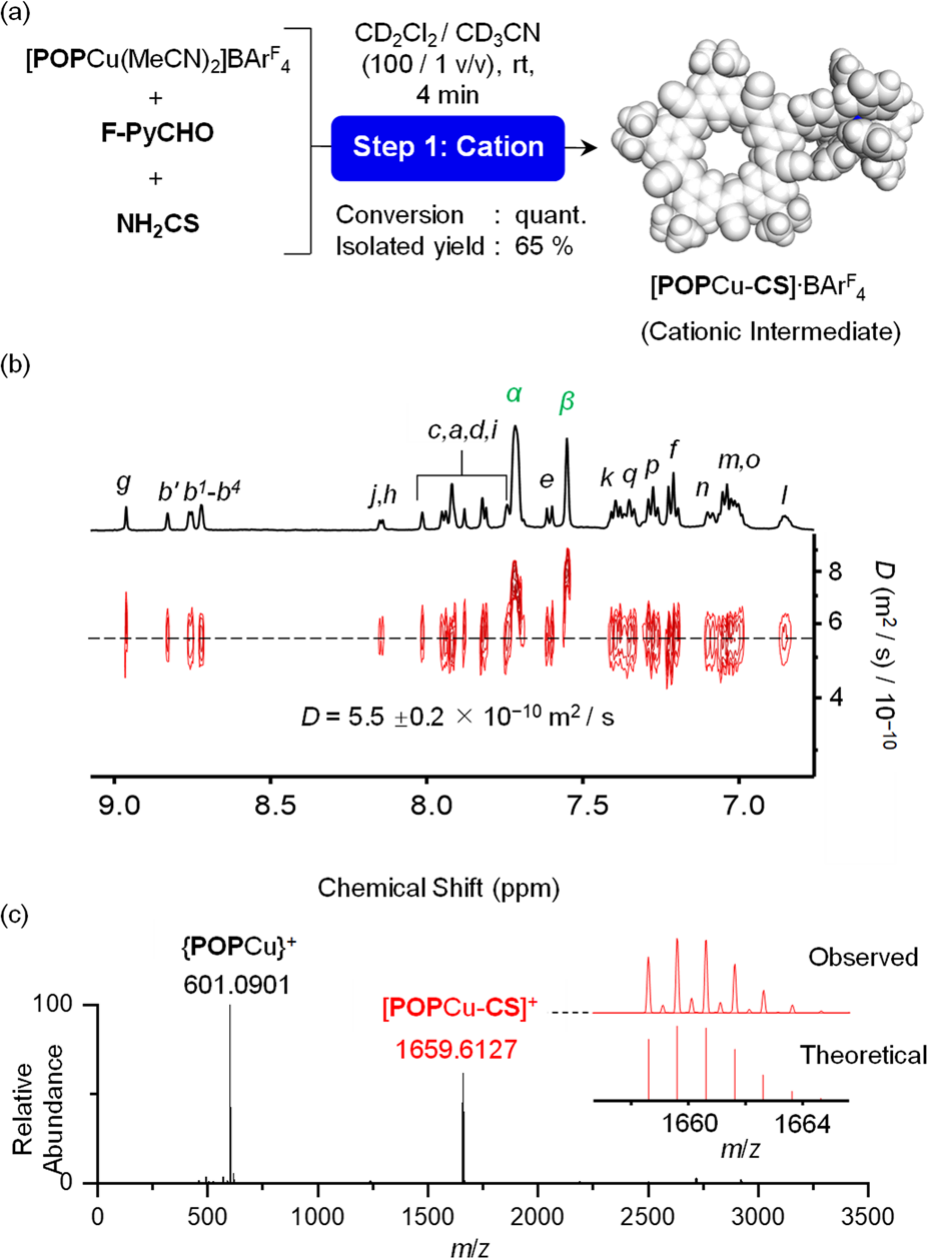
(a) Reaction to form [POPCu-CS]^+^ and characterization by (b) DOSY NMR (CD_2_Cl_2_, 2 mM), and (c) ESI-MS spectrum (CH_2_Cl_2_, 2 mM).

**Fig. 6:**
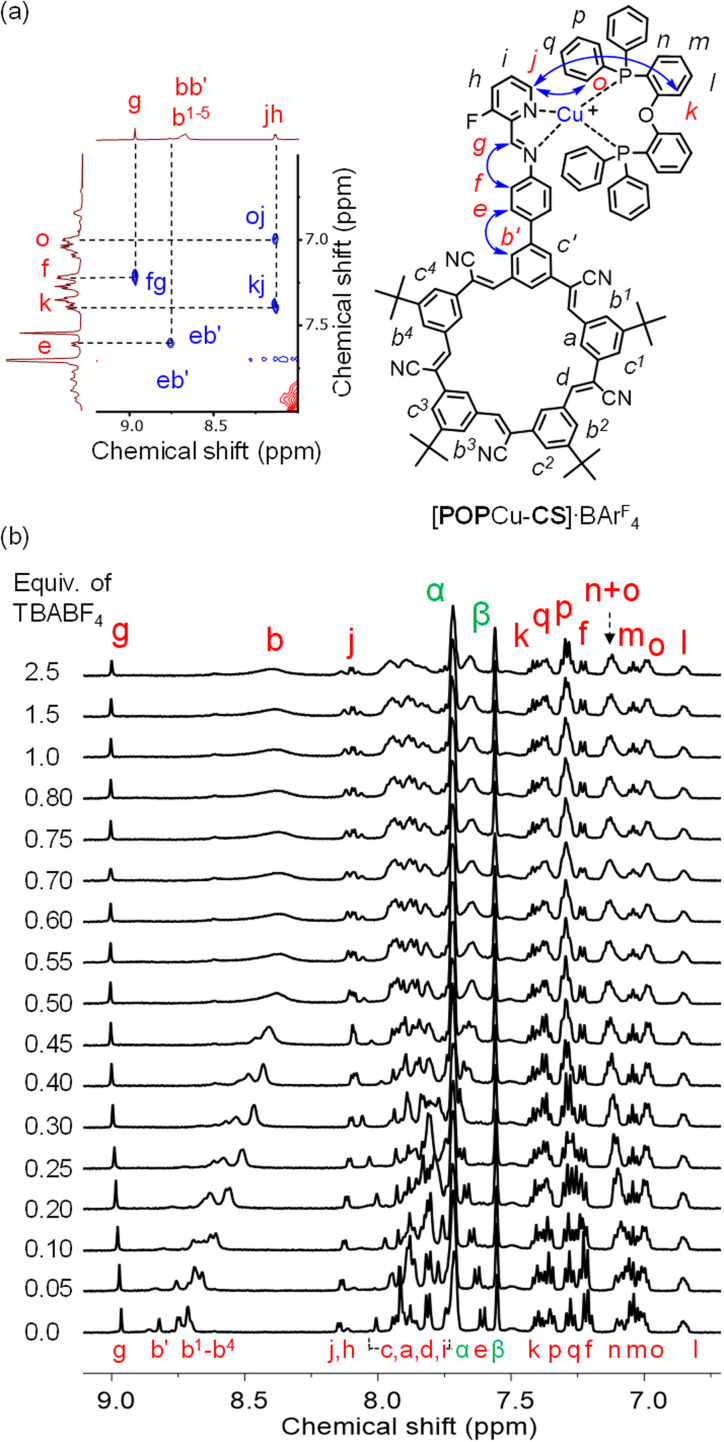
(a) The aromatic region of ROESY NMR data of [POPCu-CS]·BAr^F^_4_. (b) ^1^H NMR titration of [CS-POPCu]·BAr^F^_4_ (2 mM) with TBABF_4_ (CD_2_Cl_2_, 400 MHz, rt).

The cation-directed supramolecular intermediate was prepared under similar conditions as the one pot but using the copper precursor [POPCu(MeCN)_2_]^+^ as a BAr^F^_4_^−^ salt (Fig. 5a). Identification of the desired monomeric intermediate [POPCu-CS]^+^ was obtained from the ESI-MS with a peak at 1659.6127 *m*/*z* (Fig. 5c). DOSY confirmed formation of a single monodisperse species (Fig. 5b). Characteristic shifts in NMR peaks confirm formation of intermediate [POPCu-CS]^+^ (Fig. S54†). The BAr^F^_4_^−^ anion peaks are unshifted indicative of its innocence. ROESY data (Fig. 6) on the intermediate confirms imine formation and {POPCu}^+^ complexation.

### Modular synthesis using a universal cation-directed supramolecular intermediate with different anions

**Fig. 7:**
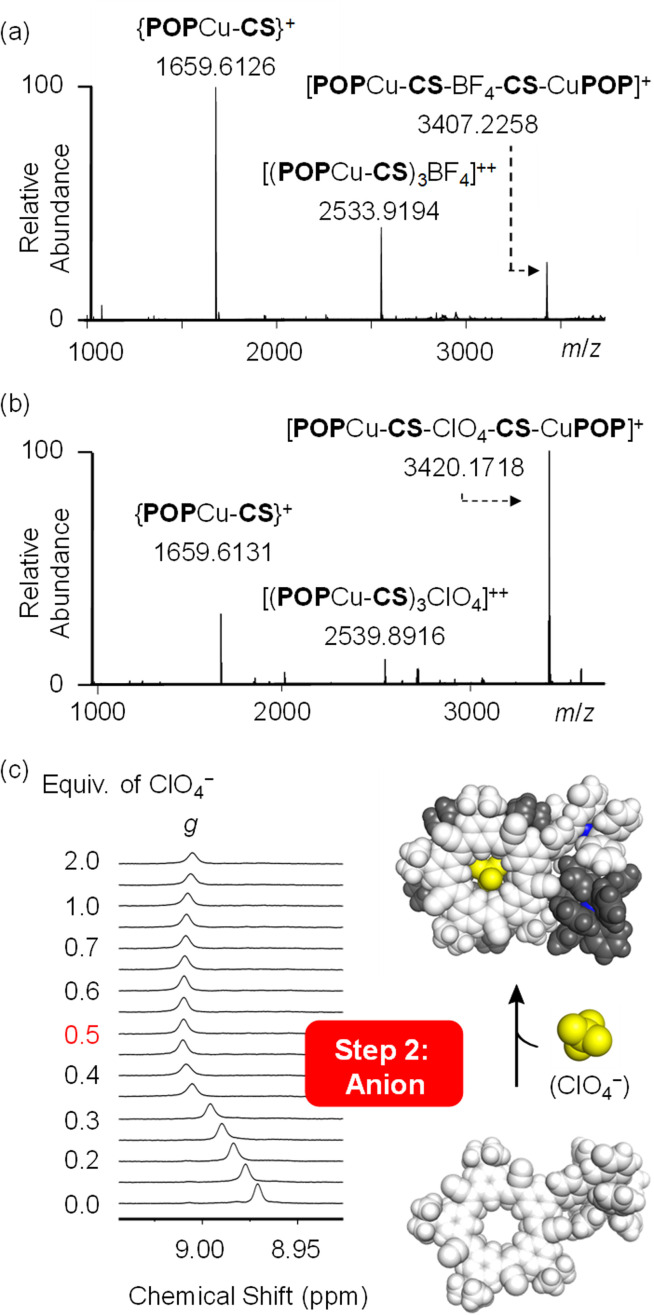
ESI-MS spectra (CH_2_Cl_2_, 0.5 mM) of (a) [POPCu-CS-BF_4_-CS-CuPOP]^+^ and (b) [POPCu-CS-ClO_4_-CS-CuPOP]^+^ formed by cation–anion pathway. (c) ^1^H NMR titrations of intermediate [CS-POPCu]·BAr^F^_4_ (2 mM) with TBAClO_4_ (CD_2_Cl_2_, 400 MHz, rt).

Preparation of the cationic intermediate [POPCu-CS]^+^ allowed investigation of different anion-driven products with BF_4_^−^ or ClO_4_^−^ (Fig. 6 and 7) to illustrate modularity. Addition of TBABF_4_ to [POPCu-CS]^+^ led to the cyanostar-mediated dimerization around the bridging BF_4_^−^ anion. ^1^H NMR titration monitoring addition of TBABF_4_ (Fig. 6b) to [POPCu-CS]^+^ indicate formation of a 2 : 1 complex with saturation at 0.5 equivalents (Fig. 6b). Diffusion NMR confirmed formation of a single monodisperse species (Fig. S62†). The dimeric assembly [POPCu-CS-BF_4_-CS-CuPOP],^+^, was verified using ESI-MS (Fig. 7a) matching the one prepared by the one-pot method (Fig. 3c). A peak at 2533.9194 *m*/*z* indicates formation of a higher-order trimer [(POPCu-CS)_3_BF_4_]^2+^ only under the ESI-MS conditions.^20,29,30,57,60^

Consistent with the modularity of the cation–anion method, addition of TBAClO_4_ generated the same types of signatures as those seen with BF_4_^−^. The dimeric assembly was seen by ESI-MS (Fig. 7b) with the same daughter and higher-order peaks. The diffusion coefficient matched the ones seen using the BF_4_^−^ anion (Fig. S63†). The ^1^H NMR titrations also showed saturation at the expected 0.5 equivalence point TBAClO_4_ (Fig. 7c).

### Stepwise synthesis using anion-linked intermediates

**Fig. 8:**
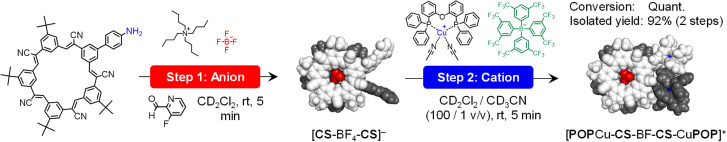
Stepwise anion–cation synthesis.

The function of the pre-programmed anion binding site can be demonstrated by inverting the ion addition sequence using an anion–cation pathway. The anion-linked supramolecular intermediate [CS-BF_4_-CS]^−^ (Fig. 8) was created by condensation of 2 equivalents each of NH_2_-CS and aldehyde F-PyCHO in the presence of 1 equivalent of TBABF_4_. The ESI-MS analyses showed the intermediate (Fig. S14†) present as a variety of MeOH adducts under the conditions of the experiment. The 1 : 4 ratio seen by NMR between the free aldehyde (F-PyCHO) and the imine suggests 80% reaction. Metal-ion chelation is required to drive complete condensation.

**Fig. 9:**
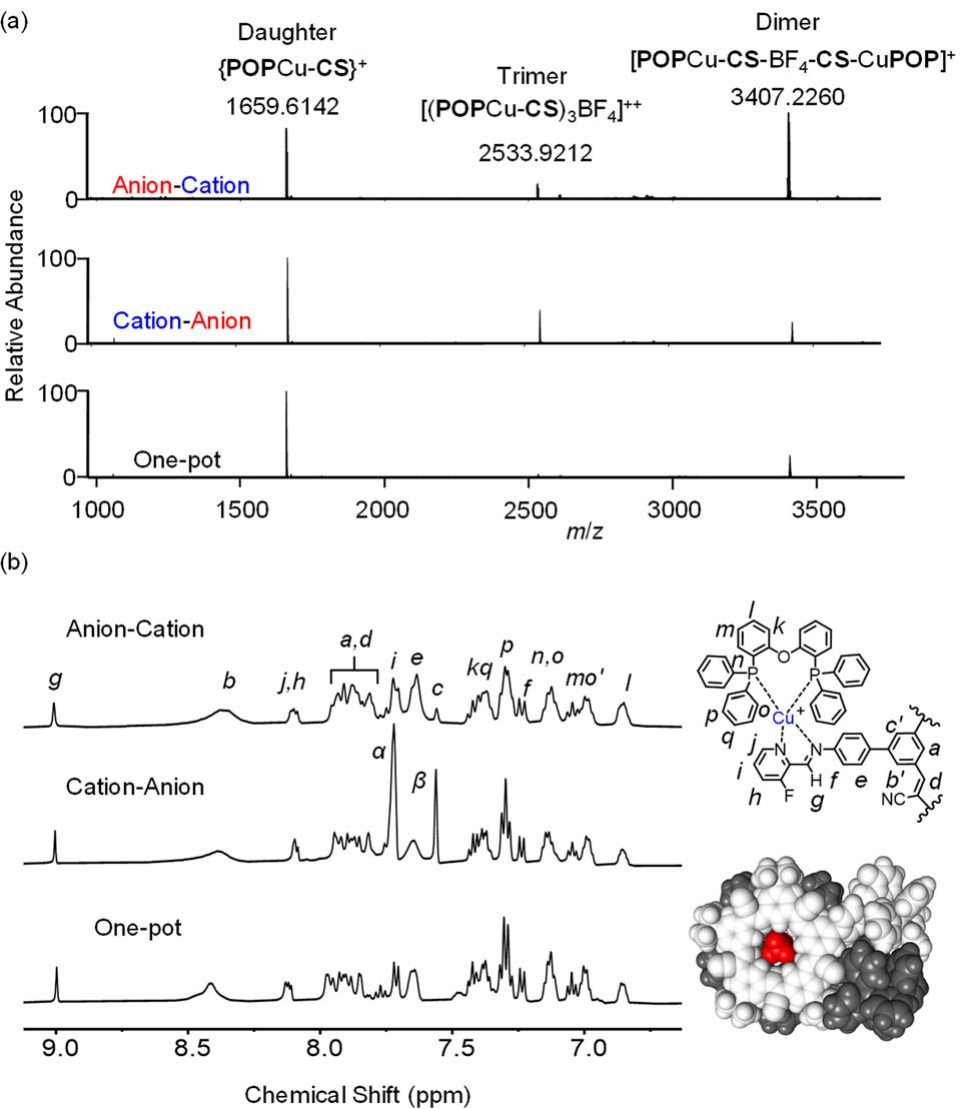
(a) ESI-MS (CH_2_Cl_2_, 0.5 mM) and (b) diagnostic ^1^H NMR peaks (CD_2_Cl_2_, 0.5 mM) for [POPCu-CS-BF_4_-CS-CuPOP]^+^ for the anion–cation stepwise, cation–anion stepwise, and one-pot assembly.

Addition of [POPCu(MeCN)_2_]^+^ in the second step (Fig. 8) drove complete formation of the imine and the target assembly, [POPCu-CS-BF_4_-CS-CuPOP]^+^. Notably, the product of the anion–cation assembly pathway shows the same diagnostic signatures in ESI-MS and NMR (Fig. 9) as the products of the one-pot and cation–anion methods confirming the same structure is made along three different synthetic pathways. The nature of the counter anions depends on the pathway used for self-assembly. In the cases where more than one counter anion is present, they are in exchange with the various species present in solution. For example, the ^19^F NMR spectrum of the gold assembly obtained *via* the cation–anion pathway shows two peaks. One results from BF_4_^−^ in fast exchange between being its cyanostar-bound and unbound states while the other corresponds to the uncomplexed NTf_2_^−^ anion (Fig. S30†).

### Orientation of the macrocycles in the dimer

**Fig. 10:**
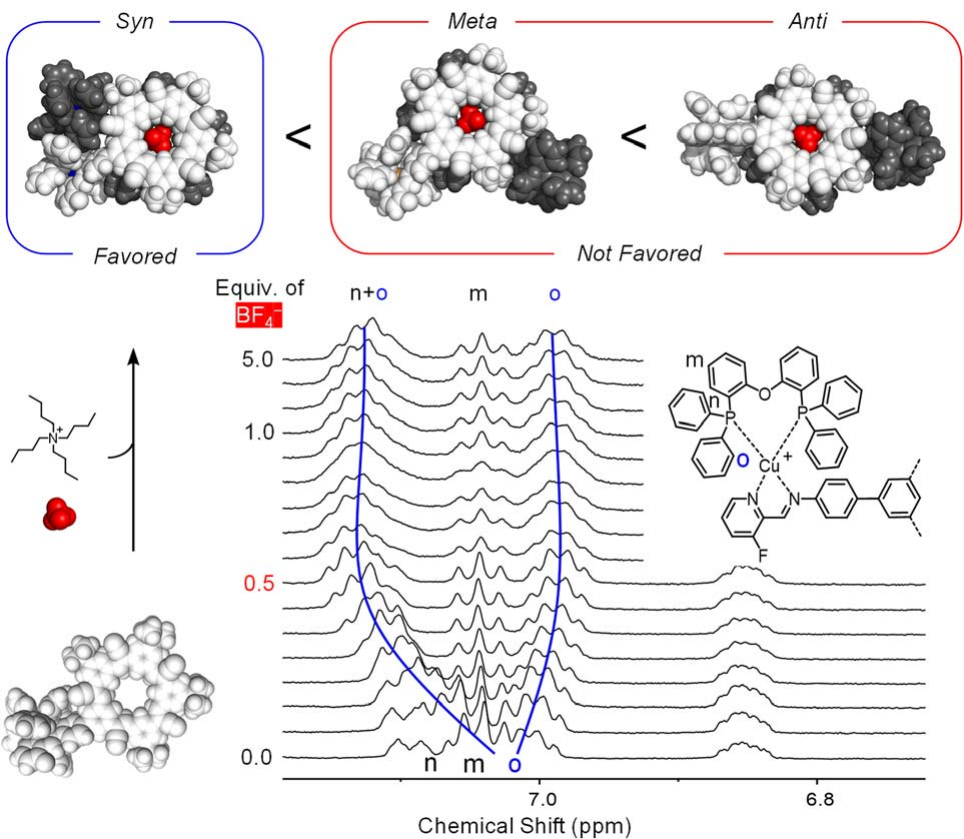
^1^H NMR titration of TBABF_4_ into [POPCu-CS]^+^ (4 mM) in CD_2_Cl_2_ (298 K, 400 MHz).

Structurally, the copper complexes in the product [POPCu-CS-BF_4_-CS-CuPOP]^+^ can be either *syn*, *meta*, or *anti* (Fig. 10). Molecular mechanics shows *syn* to be favored over *meta* (16 kJ mol^−1^) and *anti* (86 kJ mol^−1^), which is consistent with a related complex.^46^ An NMR titration following addition of BF_4_^−^ to [POPCu-CS]^+^ to form the dimerized product [CuCS-BF_4_-CSCu]^+^ supports the *syn* geometry. Therein, the phosphine ligands are close enough to lower their symmetry into two different environments (H_o_, Fig. 10). Molecular models on the *syn* geometry how one of the two phenyls engaging in π stacking. These findings suggest the *syn* geometry is favored by stabilizing contacts between coordinated POP ligands.

### Use of gold(i) showcases modularity of cation-directed supramolecular intermediates

**Fig. 11:**
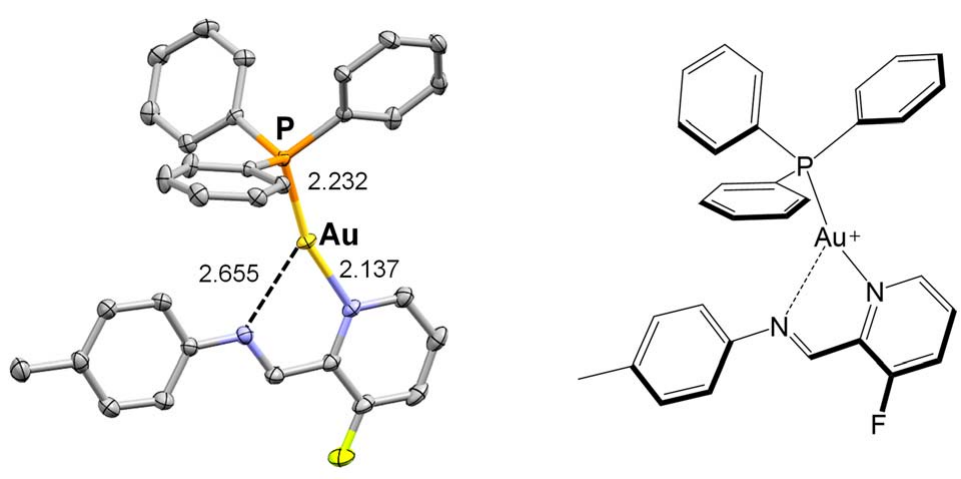
Molecular structure of the three-coordinate gold(i) model complex as determined by X-ray crystallography (50% ellipsoids). Hydrogen atoms and counter anion omitted for clarity.

Gold(i) was used to investigate the modularity of assembly. To model the first use of gold(i) ions in subcomponent self-assembly, we prepared a complex using a simple aniline. We mixed a gold(i) precursor as a triflimide salt, [PPh_3_Au]·NTf_2_ with F-PyCHO and the aniline *p*-toluidine. X-ray diffraction studies showed formation of tricoordinate gold(i) cation (Fig. 11) with a chelating imine. ^1^H and ^19^F NMR, and ESI-MS spectra (Fig. S36–S38†), are consistent with formation of the gold(i) complex seen in the solid state.

**Fig. 12:**
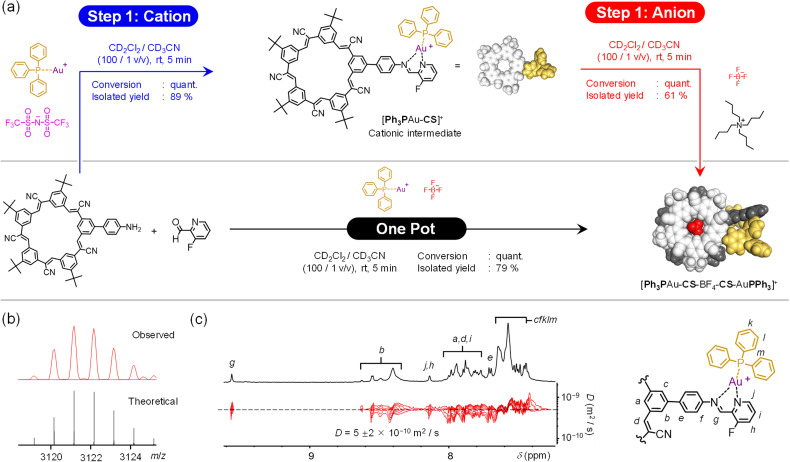
(a) Reactions to form [PPh_3_Au-CS-BF_4_-CS-AuPPh_3_]^+^ by one-pot and cation–anion pathways. (b) ESI-MS (CH_2_Cl_2_, 0.5 mM) of [PPh_3_Au-CS-BF_4_-CS-AuPPh_3_]^+^ prepared *via* cation–anion pathway. (c) DOSY NMR (CD_2_Cl_2_, 2 mM) of [PPh_3_Au-CS-BF_4_-CS-AuPPh_3_]^+^ created in one-pot assembly.

We examined the various synthetic pathways using the Au^+^ and BF_4_^−^ ions. The target complex [PPh_3_Au-CS-BF_4_-CS-AuPPh_3_]^+^ was formed along both one-pot and cation–anion pathways (Fig. 12a). Formation was confirmed by ESI-MS with the one-pot producing a peak at 3122.1669 *m*/*z* (Fig. S25†) matching the product of cation–anion assembly (Fig. 12b). Consistent with gold(i) complexes being less stable than copper(i), parent ion peaks have lower intensity than daughter ions.

**Fig. 13:**
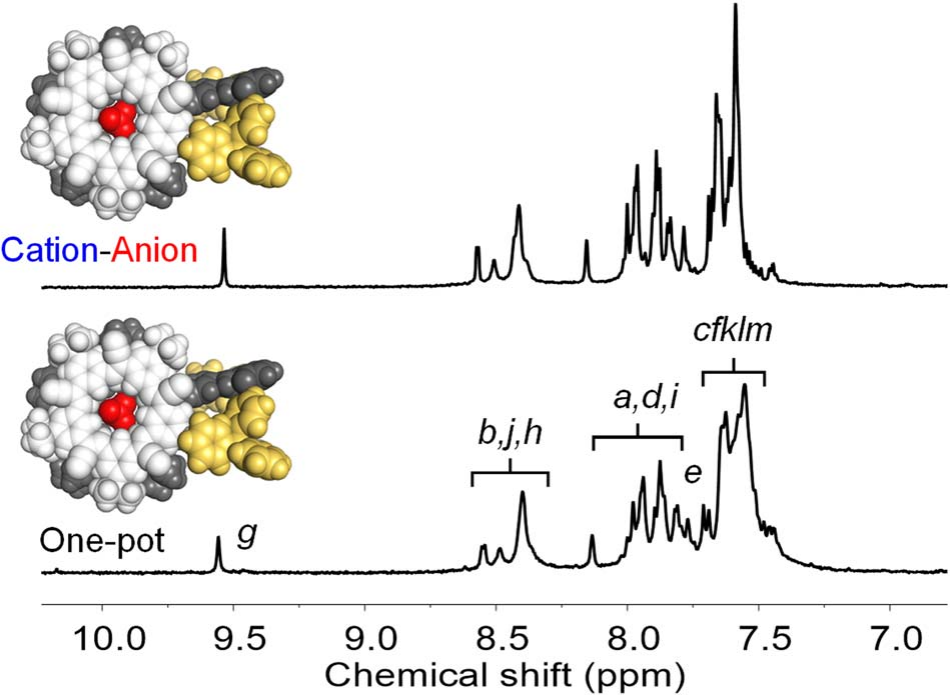
Diagnostic ^1^H NMR peaks (CD_2_Cl_2_, 0.5 mM) for [PPh_3_Au-CS-BF_4_-CS-AuPPh_3_]^+^ obtained by one-pot assembly and cation–anion assembly.

Formation of the target assembly [PPh_3_Au-CS-BF_4_-CS-AuPPh_3_]^+^, was verified by NMR. All resonances display the same diffusion coefficients (Fig. 12c) for the one-pot and cation–anion assembly (Fig. S69†) and the same diagnostic peaks by ^1^H-NMR (Fig. 13) and ^19^F-NMR spectroscopy (Fig. S27†). These similarities indicate that the same assembly is being produced along different pathways.

The anion–cation stepwise pathway was also investigated. Using the anion-linked intermediate described earlier, addition of the Au^+^ precursor produced an ^1^H NMR spectrum differing from the one-pot and cation–anion pathways (Fig. S56†). The ESI-MS did not show any peaks consistent with target assembly. Adding excess BF_4_^−^ to better match the one-pot pathway did not influence the outcome (Fig. S41†). Failure to access the target assembly by the alternative stepwise pathway is surprising and highlights the need to study the impacts of reaction order on multi-component supramolecular assemblies.

### Failure modes using competitive anions

Not every anion that binds tightly to cyanostar^29,57,60,61^ is also weakly coordinating to metal cations. We were hoping to leverage this idea to target assemblies that could only be produced by pre-complexation of the anion. To explore this idea, we studied how metal complexes survived in the presence of organophosphates, which are known to ligate metals. These phosphates are also known to form threaded pseudorotaxane complexes with cyanostar.^62^ This study was undertaken using gold and copper complexes, dibutyl phosphate, dibenzyl phosphate, and the parent cyanostar, pCS, as models. However, pre-complexation with cyanostar did not prevent the phosphates from decomposing the copper complexes in 24 hours (Fig. S71–S75†). A gold complex showed similar behavior (Fig. S76 and S77†) as did the cationic intermediate [PPh_3_Au-CS]·NTf_2_ (Fig. S77†). While we saw some evidence of a threaded pseudorotaxane, the ^1^H NMR spectra suggests that the phosphate binds to the gold ions in the complex as opposed to the cyanostar cavity (Fig. S71†). In addition, the bisulfate (HSO_4_^−^)^57,60^ and *n*-hexylphosphonate (C_6_H_13_PO_3_H^−^)^29^ anions that can drive cyanostar dimerization, behaved poorly and stripped copper ions off the complex (Fig. S71–S75†). An instantaneous loss of color was indicative of decomplexation.

Hence, testing the orthogonality of the ion interactions involved in the noncovalent bond forming reactions and verifying the compatibility of the ions with each other is a key step for successful implementation of ion-by-ion stepwise self-assembly. Use of cyanostar as a supramolecular element^41^ with its strong affinity for anions known to weakly coordinate with metals may be key to this demonstration suggesting other receptors, like bambusuril,^63–66^ may also be used in this way.

## Conclusion

We demonstrated that orthogonal cation–anion and anion–cation self-assembly can be used to build up the structures of multi-component assemblies in a modular manner by using pre-programmed binding sites for both cations and anions. This orthogonal ion-by-ion strategy of subcomponent self-assembly enables similar structures to be accessed in a modular manner using common intermediates in both a one-pot and stepwise way. The architectures involved Cu(i) and Au(i) complexes situated on the termini of the anion-driven cyanostar dimers form [POPCu-CS-X-CS-CuPOP]^+^ and [PPh_3_Au-CS-X-CS-AuPPh_3_]^+^, where the bridging anion (X) can be either BF_4_^−^ or ClO_4_^−^. We find that the noncovalent chemistries of anion and cation coordination can be readily paired with the covalent chemistry of sub-component self-assembly based on modular imine-based ligands. The combination of anion receptors with established ligands for metal complexation introduce a new approach to design architectures based on orthogonal cation–anion self-assembly.

## Data availability

The ESI is available free of charge on the journal website. General methods, NMR titrations, 2D NMR spectroscopy, X-ray diffraction analyses, and ESI-MS analyses.

## Author contributions

AD conceived the project under the supervision of AHF; in response to the Covid pandemic, REF assisted AHF with overseeing project completion. AD conducted experiments with assistance from LAK, YC, REF, DVC and VC; AD designed, created and characterized the assemblies with initial input from LAK; YC, REF and LAK made and characterized compounds and collected control data; DVC grew a single crystal and VC collected X-ray diffraction data, solved and refined the crystal structure; AD, REF and AHF analyzed the data, wrote and edited the manuscript with input from all co-authors.

## Conflicts of interest

The authors declare no competing financial interests.

## Supplementary Material

SC-014-D2SC05121D-s001

SC-014-D2SC05121D-s002
