# Supplementary material for: Orthogonal, modular anion–cation and cation–anion self-assembly using pre-programmed anion binding sites
Source: Chem Sci. 2023 Feb 3;14(10):2585–95. doi: 10.1039/d2sc05121d (PMC9993851; doi:10.1039/d2sc05121d)
Supplement: SC-014-D2SC05121D-s001 [file SC-014-D2SC05121D-s001.pdf]

Electronic Supplementary Material (ESI) for Chemical Science.
This journal is © The Royal Society of Chemistry 2023

Supporting Information

Chem. Sci.

Orthogonal, Modular Anion-Cation and Cation-Anion Self-assembly using Pre-programmed Anion Binding Sites

Ayan Dhara,[ab] Rachel E. Fadler,[a] Yusheng Chen,[a] Laura A. Köttner,[ac] David Van Craen,[ad] Veronica Carta,[a] and Amar H. Flood*[a]

[a] Department of Chemistry, Indiana University, 800 East Kirkwood Avenue, Bloomington, IN 47405, USA.

[b] *Current address:* Department of Chemistry and Biochemistry, University of Windsor, Windsor, Ontario, N9B 3P4, Canada.

[c] *Current address:* Department of Chemistry and Pharmacy, Friedrich-Alexander-Universität Erlangen-Nürnberg, Nikolaus-Fiebiger-Str. 10, 91058 Erlangen, Germany.

[d] *Current address:* Department of Chemistry and Chemical Biology, Technische Universität Dortmund, Otto-Hahn-Str. 6, 44227 Dortmund, Germany.

Table of Contents

1. **General Comments** 3
2. **One-pot Self-assembly of** [**POP**Cu-**CS**-BF_4-**CS**-Cu**POP**]·BF_4 3
3. **Self-assembly of** [**POP**Cu-**CS**]·BAr^F_4 6
4. **Titration of** [**POP**Cu-**CS**]·BAr^F_4 **with** $TBABF_4$ 8
5. **Self-assembly of** [**POP**Cu-**CS**-BF_4-**CS**-Cu**POP**]$^+$ **by Cation-Anion Pathway** 9
6. **Self-assembly of** [**CS**-BF_4-**CS**]$^-$ 11
7. **Self-assembly of** [**POP**Cu-**CS**-BF_4-**CS**-Cu**POP**]$^+$ **by Anion-Cation Pathway** 12
8. **Self-assembly of** [**Ph$_3$P**Au-**CS**]·NTf_2 14
9. **Self-assembly of** [**PPh$_3$**Au-**CS**-BF_4-**CS**-Au**PPh$_3$**]$^+$ **by One-pot** 17
10. **Titration of** [**Ph$_3$P**Au-**CS**]·NTf_2 **with** $TBABF_4$ 19
11. **Self-assembly of** [**PPh$_3$**Au-**CS**-BF_4-**CS**-Au**PPh$_3$**]$^+$ **by Cation-Anion Pathway** 20
12. **Self-assembly of Cyanostar-based Gold(I) Assembly by Anion-Cation Pathway** 23
13. **Self-assembly of** [**CS**-BF_4-**CS**]$^-$ **in Presence of Excess TBABF$_4$** 25
14. **Self-assembly of Cyanostar-based Gold(I) Assembly by Anion-Cation Pathway in Presence of Excess TBABF$_4$** 27
15. **Self-assembly of Gold(I) Model Complex** 30
16. **Synthesis of** [**POP**Cu]·BAr^F_4 31
17. **NOESY and ROESY Characterization** 33
18. **Diagnostic ^{1}H NMR Shifts upon Self-assembly** 35
19. **^{1}H NMR Spectra of Gold(I) Assemblies Obtained by Different Pathways** 37
20. **Titration of** [**POP**Cu-**CS**]·BAr^F_4 **with** $TBAClO_4$ 37
21. **^{1}H NMR Titration Controls with Cyanostar** 38
22. **Diffusion NMR: Pulse Gradient Spin Echo (PGSE) experiments** 39
23. **Orientation of the Macrocycles in the Dimer** 53
24. **Choice of Anions for the Dimerization of Cationic Intermediates and Follow-Up Studies to Investigate Viability of Sequence-Dependent Target Product** 53
25. **Molecular Mechanics** 61
26. **X-Ray Crystallography** 91
27. **References** 94

1. General Comments

All reagents were obtained from commercial suppliers and used as received unless otherwise noted. The aminocyanostar macrocycle (**NH_2-CS**) was synthesized from iodo-cyanostar (**CS-I**) according to the reported literature procedure.[1] Column chromatography was performed on silica gel (40-63 μm). Thin layer chromatography (TLC) was performed on pre-coated silica gel plates (250 μm thick, TLG-R10011B-323, Silicycle, Canada) and observed under UV light.

Trifluoromethyl toluene (Ph-CF_3) was used as an internal standard for the collection of ^{19}F NMR spectra. The ^{19}F NMR peak of Ph-CF_3 in CD_2Cl_2 was referenced at –62.93 ppm based on the $CFCl_3$-calibration studies for ^{19}F NMR references in various deuterated solvents.[2] All ^{19}F NMR spectra were collected below 10 mM to eliminate any concentration effects.[2]

For all ^1H NMR titrations a solution of the cyanostar derivative was placed in an NMR tube, sealed with a rubber septum and an initial ^1H NMR spectrum was recorded. Aliquots of a solution of the corresponding salt were then added with a microsyringe through the rubber septum. ^1H NMR spectra were recorded after each addition.

For ^{19}F NMR titrations, a CD_2Cl_2 solution of the cyanostar derivative was placed in an NMR tube, 10 μL of trfluorotoluene (Ph-CF_3) was added in the solution for standardization of chemical shifts of ^{19}F peaks. Afterwards, the NMR tube was sealed with a rubber septum and an initial ^{19}F NMR spectrum was recorded. Another ^1H NMR spectrum was also recorded to verify that the Ph-CF_3 had no interaction with the cyanostar derivative. Aliquots of a solution of the corresponding salt were added with a microsyringe through the rubber septum and ^{19}F NMR spectra were recorded after each addition.

Nuclear magnetic resonance (NMR) spectra were recorded on a Varian Inova (600 MHz, 500 MHz, 400 MHz) and Varian VXR (400 MHz) spectrometers at room temperature (298 K). Chemical shifts were referenced to residual solvent peaks. The pulse gradient spin echo (PGSE) experiments provided the diffusion coefficients. The NMR peaks were analyzed to determine diffusion coefficients using Vnmrj's analysis software. Average diffusion coefficients and errors were generated from multiple ^1H NMR peaks. High-resolution electrospray ionization (ESI) mass spectrometry was performed on a Thermo Electron Corporation MAT 95XP-Trap mass spectrometer.

2. One-pot Self-assembly of [**POP**Cu-**CS**-BF_4-**CS**-Cu**POP**]·BF_4

Amino-cyanostar macrocycle **NH_2-CS** (4.0 mg, 0.0042 mmol, 1 equivalent), fluorinated pyridyl-2-aldehyde **F-PyCHO** (1.0 mg, 0.0084 mmol, 2 equivalent) and the phosphine-chelated copper salt **POP**Cu-**BF_4** (3.2 mg, 0.0042 mmol, 1 equivalent) were taken in a 20-dram glass vial and dichloromethane (0.6 mL) was added in the vial. A red solution was obtained immediately. To the solution, 10 μL acetonitrile was added. Solvents were evaporated by blowing argon. The ^1H NMR spectrum (CD_2Cl_2) of the resulting solid precipitate showed the clean formation of [**POP**Cu-**CS**-BF_4-**CS**-Cu**POP**]·BF_4 along with the presence of excess aldehyde, which was removed by washing the crude precipitate with diethyl ether (3 × 2 mL). While washing with Et_2O, some of the

precipitate was washed away, which resulted in isolated yields less than quantitative. The final precipitate (8.7 mg, 0.0025 mmol, 60 %) was dried under vacuum. The integration of ^{1}H NMR peaks are normalized to one cyanostar in assembly.

^{1}H NMR (CD_2Cl_2, 500 MHz, rt): δ 9.00 (s, 1H), 8.41 (br s, 5H), 8.16-8.08 (s, 2H), 7.99-7.82 (m, 10H), 7.78 (d, *J* = 8,8 Hz, 1H), 7.73-7.70 (m, 2H), 7.69-7.59 (m, 5H), 7.44-7.41 (m, 2H), 7.40-7.35 (m, 4H), 7.30 (q, *J* = 7.8 Hz, 8H), 7.24 (d, *J* = 8.0 Hz, 2H), 7.17-7.08 (m, 6H), 7.05 (t, *J* = 7.5 Hz, 2H), 7.02-6.95 (m, 4H), 6.89-6.82 (m, 2H), 1.57-1.53 (m, 36H, overlapping with residual H_2O peak). ^{19}F NMR (CD_2Cl_2, 500 MHz, rt): δ −122.42, −149.88. HRMS (ESI) *m/z*: [**POP**Cu-**CS**-BF_4-**CS**-Cu**POP**]$^+$ Calcd for $C_{218}H_{184}BCu_2F_6N_{14}O_2P_4^+$ 3407.2328; Found 3407.2455

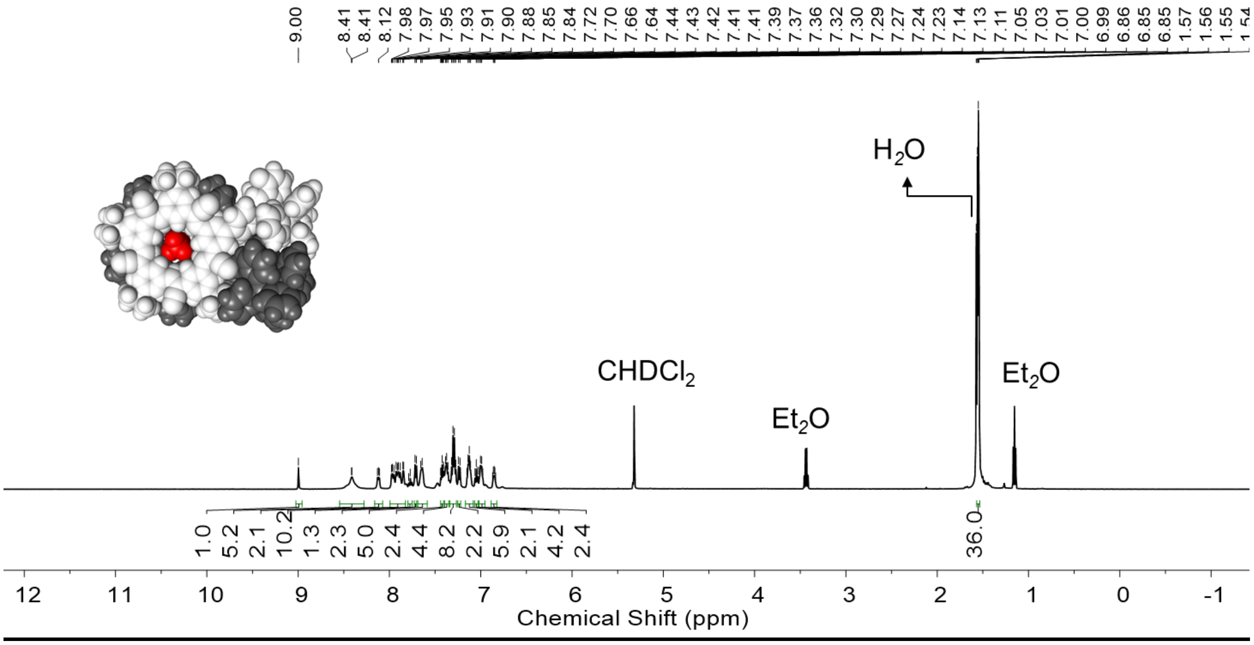

Figure S1. ^{1}H NMR of [**POP**Cu-**CS**-BF_4-**CS**-Cu**POP**]·BF_4 by one-pot (500 MHz, CD_2Cl_2, rt).

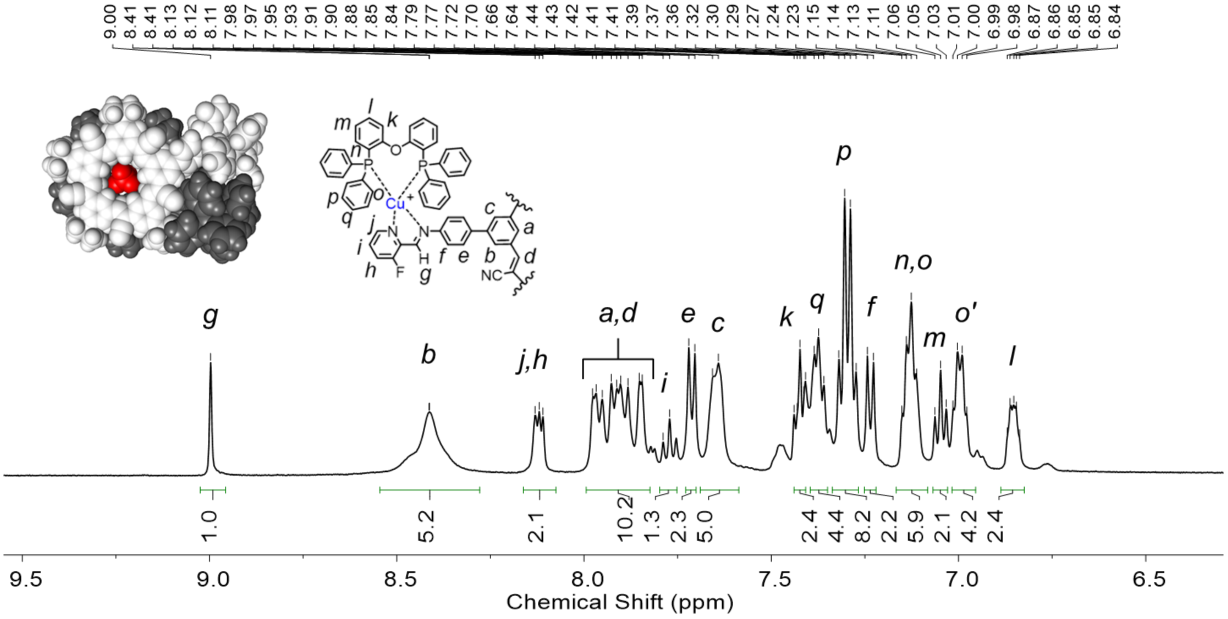

Figure S2. The aromatic region of ^{1}H NMR (Figure S1) of [**POP**Cu-**CS**-BF_4-**CS**-Cu**POP**]·BF_4 by one-pot (500 MHz, CD_2Cl_2, rt).

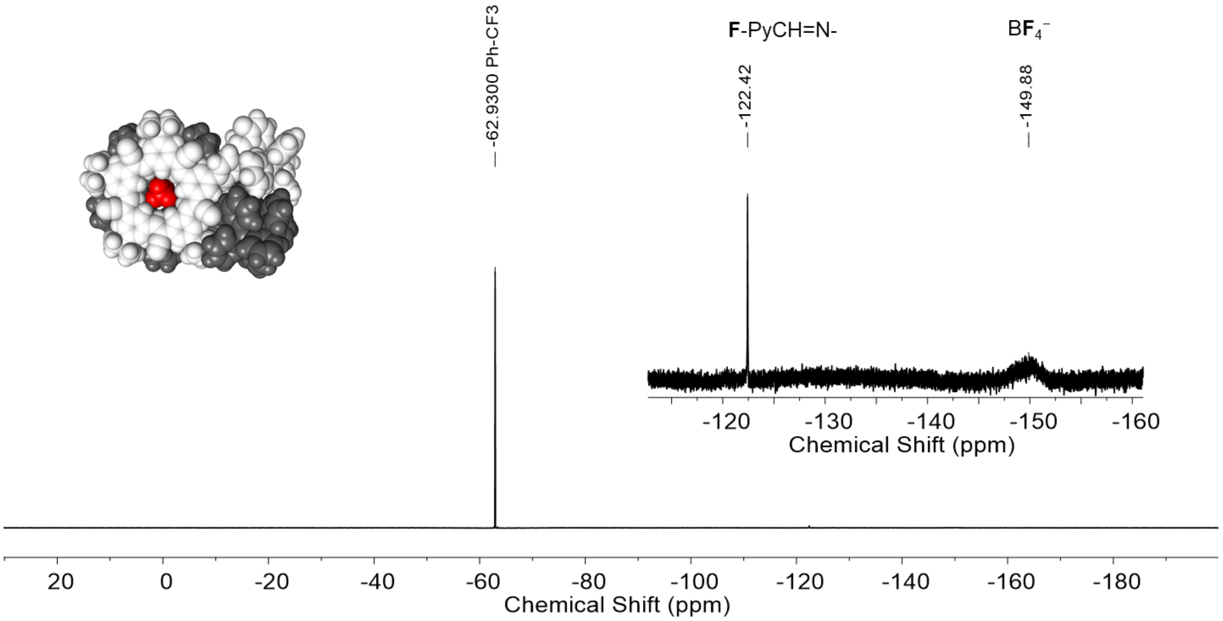

Figure S3. ^{19}F NMR of [**POP**Cu-**CS**-BF_4-**CS**-Cu**POP**]·BF_4 by one-pot (376 MHz, CD_2Cl_2, rt).

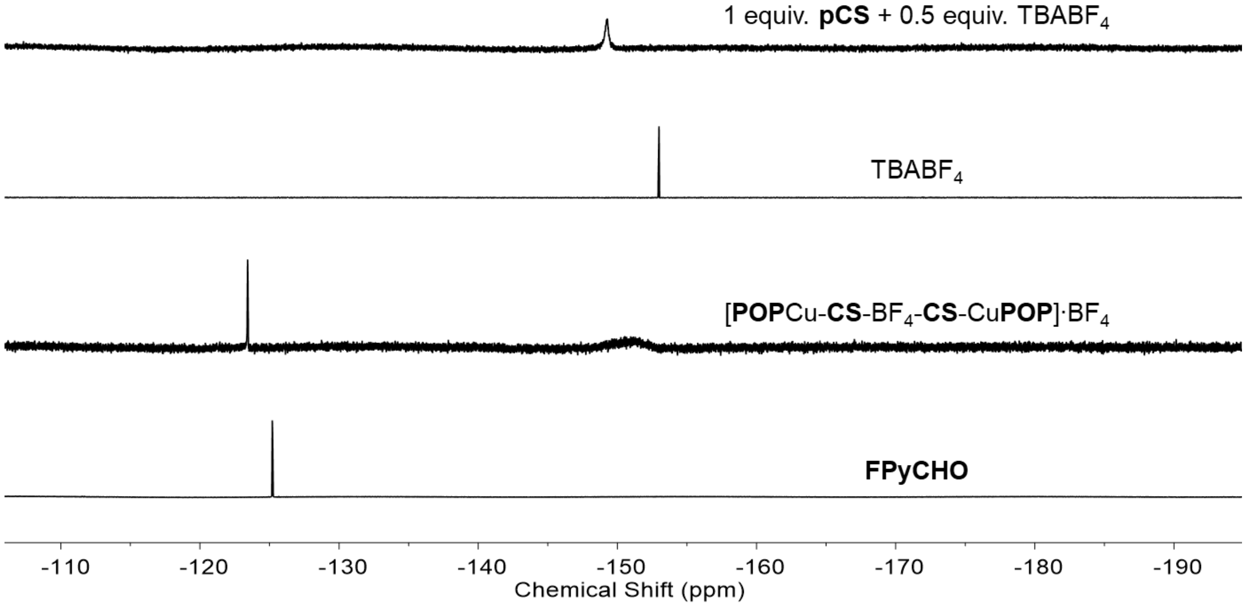

Figure S4. Comparison of ^{19}F NMR spectra of [**POP**Cu-**CS**-BF_4-**CS**-Cu**POP**]·BF_4 by one-pot with other relevant fluorinated moieties (376 MHz, CD_2Cl_2, rt).

3. Self-assembly of [POPCu-CS]·BAr^F_4

NH_2-CS (2.4 mg, 0.0025 mmol, 1 equivalent), **F-PyCHO** (0.9 mg, 0.0075 mmol, 3 equivalent) and the phosphine-chelated copper salt [**POP**Cu-**CS**]·BAr^F_4 (3.9 mg, 0.0025 mmol, 1 equivalent) were taken in a 20-dram glass vial and 1.0 mL dichloromethane was added in the vial. A red solution was obtained immediately. To the solution, 10 µL acetonitrile was added. The solvents were then evaporated by blowing argon. The orange-red precipitate was dissolved in minimum amount of dichloromethane and the addition of *n*-hexane yielded [**POP**Cu-**CS**]·BAr^F_4 (4.1 mg, 0.0016 mmol, 65 %) as a red precipitate at room temperature.

^1H NMR (CD_2Cl_2, 500 MHz, rt): δ 8.96 (s, 1H), 8.83 (s, 1H), 8.78-8.71 (m, 4H), 8.15 (d, *J* = 4.8 Hz, 2H), 8.03-7.86 (m, 10H), 7.83-7.80 (m, 3H), 7.76-7.73 (m, 3H), 7.71 (br s, 8H), 7.61 (d, *J* =8.4 Hz, 2H), 7.55 (s, 4H), 7.39 (dd, *J* = 8.2, 5.8 Hz, 4H), 7.36-7.34 (m, 2H), 7.28 (t, *J* = 7.5 Hz, 5H), 7.21 (t, *J* = 7.7 Hz, 5H), 7.09 (d, *J* = 8.3 Hz, 2H), 7.06-7.00 (m, 10H), 6.87-6.83 (m, 2H), 1.52-1.48 (m, 36H). ^{19}F NMR (CD_2Cl_2, 376 MHz, rt): δ − 122.45. HRMS (ESI) *m/z*: [**POP**Cu-**CS**]$^+$ Calcd for $C_{109}H_{92}CuFN_7OP_2^+$ 1659.6147; Found 1659.6127

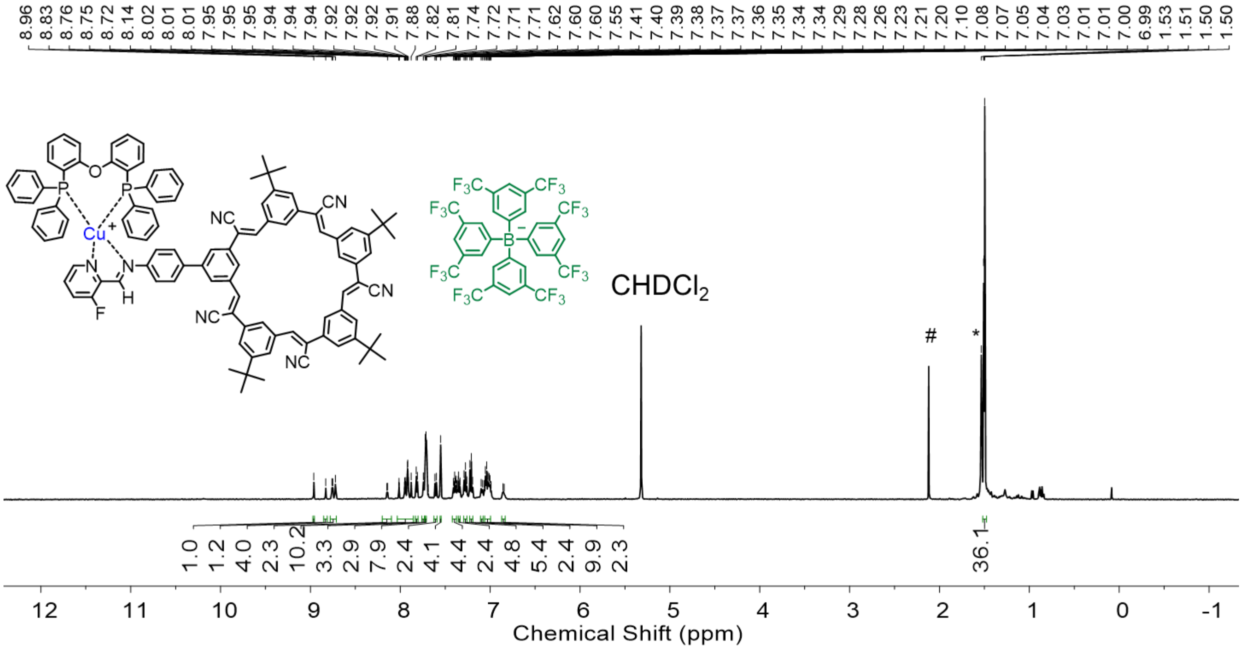

Figure S5. ^{1}H NMR of [**POP**Cu-**CS**]·BAr^{F}_{4} (500 MHz, CD_2Cl_2, rt). * = H_2O, # = acetone

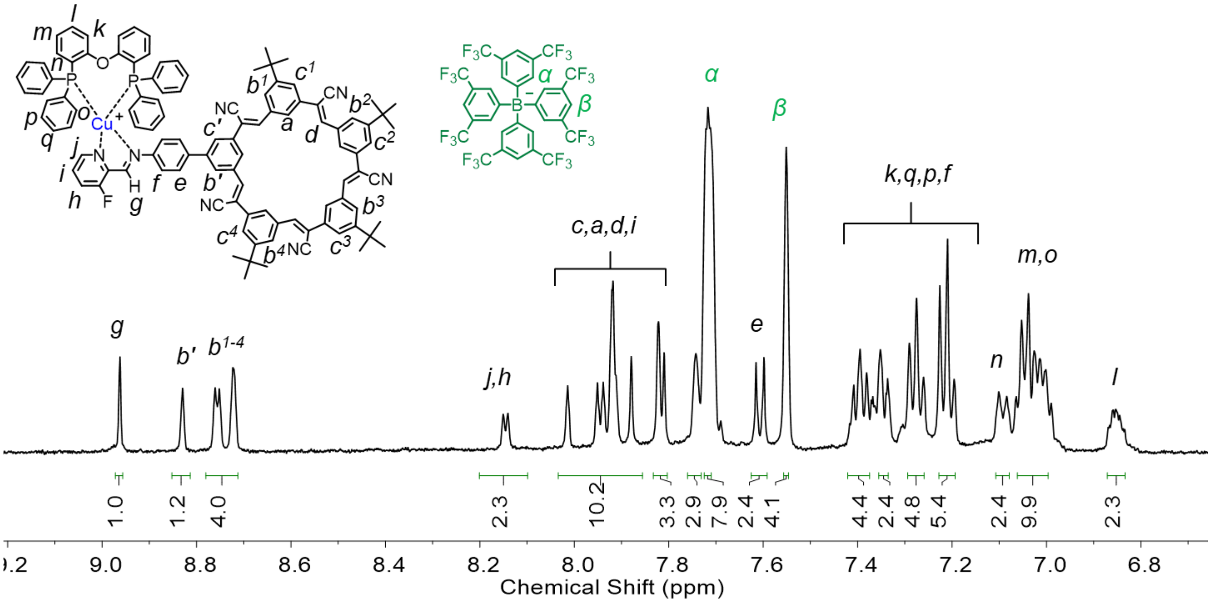

Figure S6. The aromatic region of ^{1}H NMR (Figure S5) of cationic intermediate [**POP**Cu-**CS**]·BAr^{F}_{4} (500 MHz, CD_2Cl_2, rt)

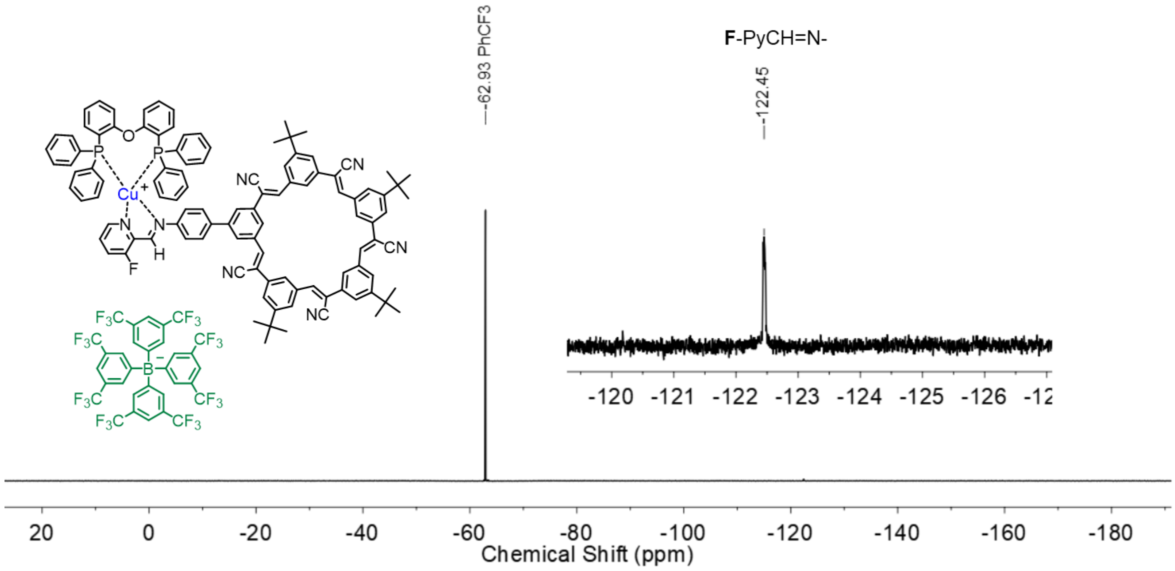

Figure S7. ^{19}F NMR of [**POP**Cu-**CS**]·BAr$^F{}_4$ (376 MHz, CD_2Cl_2, rt). The peaks corresponding to the BAr$^F{}_4$ anion overlaps with the reference peak of Ph-CF_3. The ^{19}F NMR peaks of different alkali and ammonium BAr$^F{}_4$ salts appear between 62.5 ppm and 63.3 ppm.[3, 4]

4. Titration of [POPCu-CS]·BAr$^F{}_4$ with TBABF$_4$

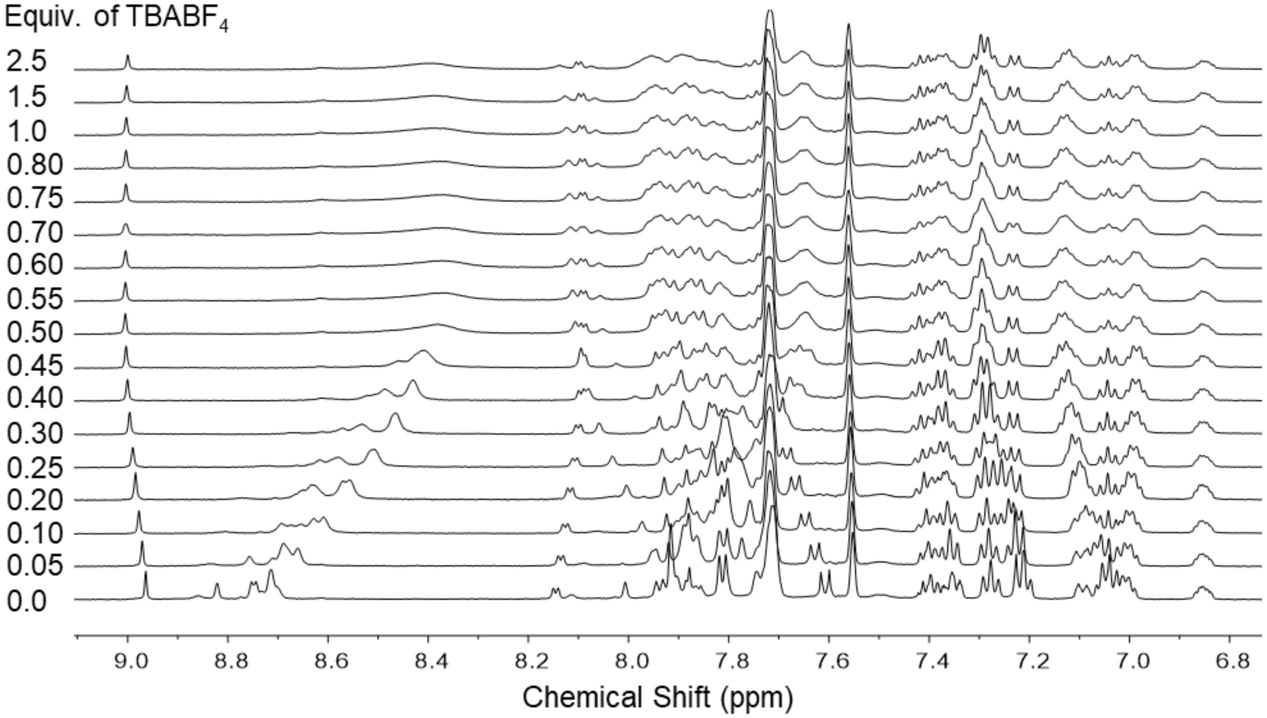

Figure S8. ^{1}H NMR spectra for the titration of [**POP**Cu-**CS**]·BAr$^F{}_4$ (the bottom spectrum, 1.8 mM) with TBABF$_4$ (with increasing equivalents from bottom to top: 0, 0.05, 0.10, 0.20, 0.25, 0.30, 0.40, 0.45, 0.50, 0.55, 0.60, 0.70, 0.75, 0.80, 1.0, 1.5, 2.5) (500 MHz, CD_2Cl_2, rt).

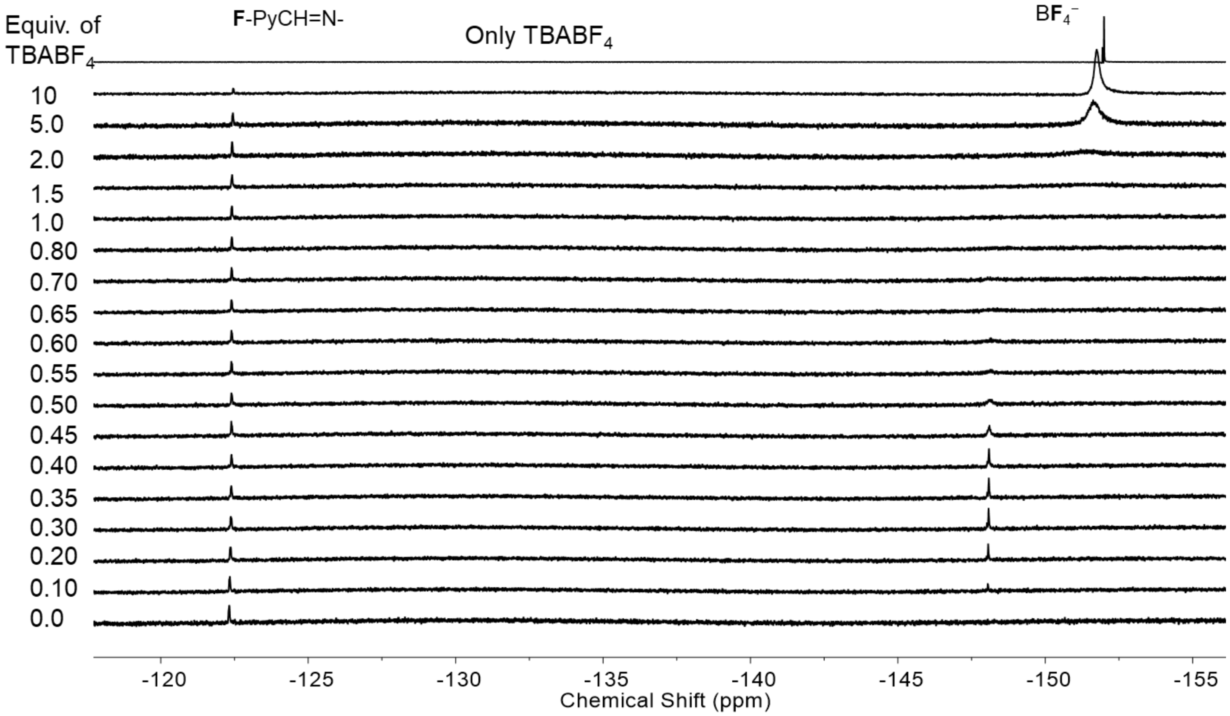

Figure S9. ^{19}F NMR spectra for the titration of [**POP**Cu-**CS**]·BAr^F_4 (the bottom spectrum, 1.8 mM) with $TBABF_4$ (with increasing equivalents from bottom to top: 0, 0.1, 0.2, 0.3, 0.35, 0.4, 0.45, 0.5, 0.55, 0.6, 0.65, 0.7, 0.8, 1.0, 1.5, 2.0, 5.0, 10.0) and the top ^{19}F NMR spectrum for only $TBABF_4$ (376 MHz, CD_2Cl_2, rt).

5. Self-assembly of [POPCu-CS-BF_4-CS-CuPOP]$^+$ by Cation-Anion Pathway

In a 20-dram glass vial, [**POP**Cu-**CS**]·BAr^F_4 (6.2 mg, 0.0042 mmol, 1 equivalent) and $TBABF_4$ (0.4 mg, 0.0013 mmol, 0.5 equivalent) were taken and 1 mL dichloromethane was added to the vial. A red solution was obtained. The solvents were evaporated by blowing argon to obtain [**POP**Cu-**CS**-BF_4-**CS**-Cu**POP**]·BAr^F_4 as a red solid, which was then dissolved in CD_2Cl_2 and subjected to characterization. NMR spectroscopy showed the quantitative formation of [**POP**Cu-**CS**-BF_4-**CS**-Cu**POP**]$^+$ when compared to the ^{1}H NMR of the assembly obtained in one-pot (Figure S1, S2). The integration of ^{1}H NMR peaks are normalized to one cyanostar in assembly.

^{1}H NMR (CD_2Cl_2, 500 MHz, rt): δ 9.03-8.97 (s, 1H), 8.50-8.29 (br s, 5H), 8.15-8.06 (m, 2H), 8.03-7.74 (m, 13H), 7.72 (s, 8H), 7.69-7.59 (br s, 5H), 7.56 (s, 4H), 7.45-7.26 (m, 14H), 7.24 (d, J = 8.1 Hz, 2H), 7.18-6.93 (m, 12H), 6.89-6.83 (m, 2H), 1.60-1.54 (m, 36H). HRMS (ESI) *m/z*: [**POP**Cu-**CS**-BF_4-**CS**-Cu**POP**]$^+$ Calcd for $C_{218}H_{184}BCu_2F_6N_{14}O_2P_4^+$ 3407.2328; Found 3407.2258 (Note: A peak at 2533.9194 *m/z* indicates formation of a higher-order trimer [(**POP**Cu-**CS**)$_3BF_4$]$^{2+}$ only under the ESI-MS conditions.[5-9])

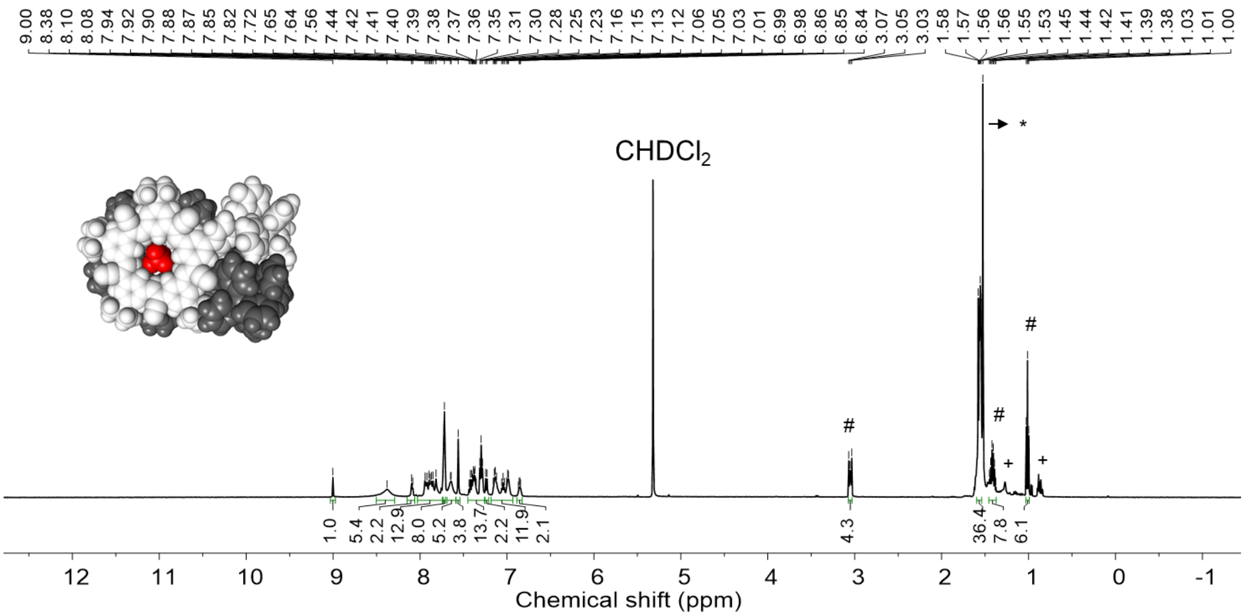

Figure S10. ^{1}H NMR of [**POP**Cu-**CS**-BF$_4$-**CS**-Cu**POP**]$^+$ (500 MHz, CD_2Cl_2, rt). # = TBA^+, * = H_2O, + = H-grease.

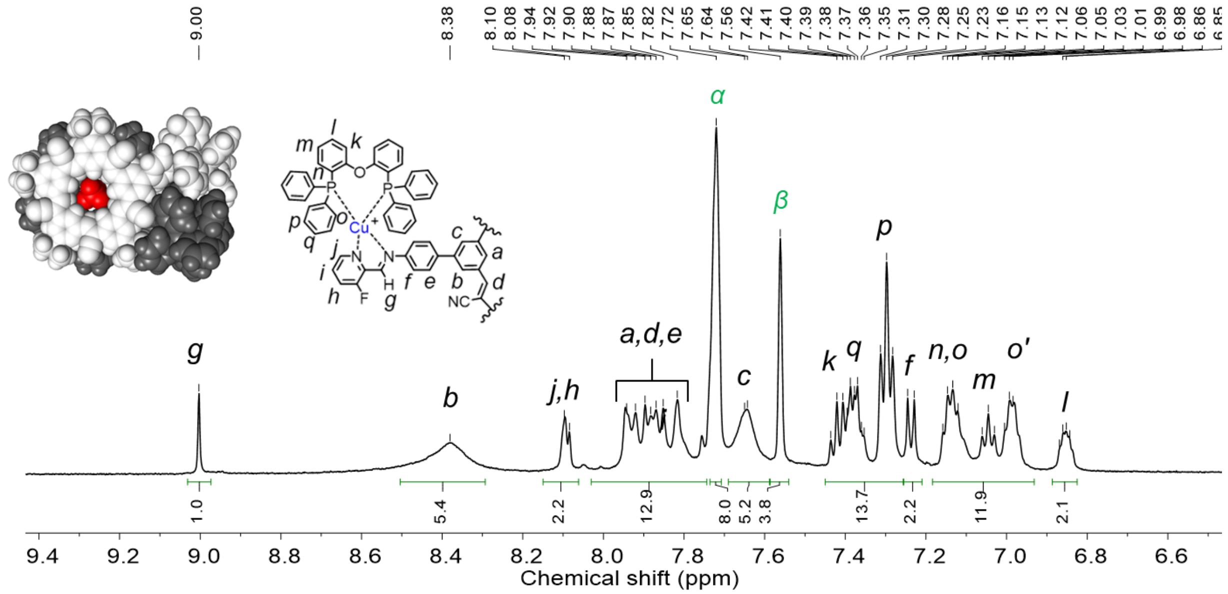

Figure S11. The aromatic region of ^{1}H NMR (Figure S10) of [**POP**Cu-**CS**-BF$_4$-**CS**-Cu**POP**]$^+$ (500 MHz, CD_2Cl_2, rt). α and β: $BAr^F{}_4{}^-$

6. Self-assembly of [**CS**-BF_4-**CS**]$^-$

NH_2-CS (3.5 mg, 0.004 mmol, 1 equivalent), **F-PyCHO** (0.92 mg, 0.008 mmol, 2 equivalent) and $TBABF_4$ (3.9 mg, 0.012 mmol, 3 equivalent) were taken in a 20-dram glass vial and 1.0 mL dichloromethane was added in the vial. Excess $TBABF_4$ was used in the reaction to ensure that all macrocycles form 2:1 cyanostar:BF_4^- complexes in solution. A yellow solution was obtained. The solvents were evaporated by blowing argon. The solid precipitate was washed with Et_2O (3×1 mL) and dried to obtain the final assembly (5.4 mg, 0.0022 mmol) under vacuum. The precipitate was dissolved in CD_2Cl_2 and the resulting solution was characterized by ^{1}H NMR spectroscopy, ESI-MS spectrometry and PGSE diffusion experiments. The intermediate was directly used in the next step without further purification. The integration of ^{1}H NMR peaks are normalized to one cyanostar in assembly. ^{1}H NMR (CD_2Cl_2, 500 MHz, rt): δ 8.94 (s, 1H), 8.64 (d, *J* = 4.4 Hz, 1H), 8.49-8.34 (m, 5H), 8.05 (s, 1H), 7.95-7.78 (m, 12H), 7.68-7.58 (m, 6H), 7.56 (d, *J* = 8.3 Hz, 2H), 7.51-7.46 (m, 1H), 6.92 (d, *J* = 8.3 Hz, 1H), 1.60-1.56 (m, 34H, overlaps with H_2O). HRMS (ESI) *m/z*: [**CS-**BF_4**-CS**]$^-$ Calcd for [$C_{146}H_{128}BF_6N_{14}$ + MeOH]$^-$ 2235.0745; Found 2235.0863

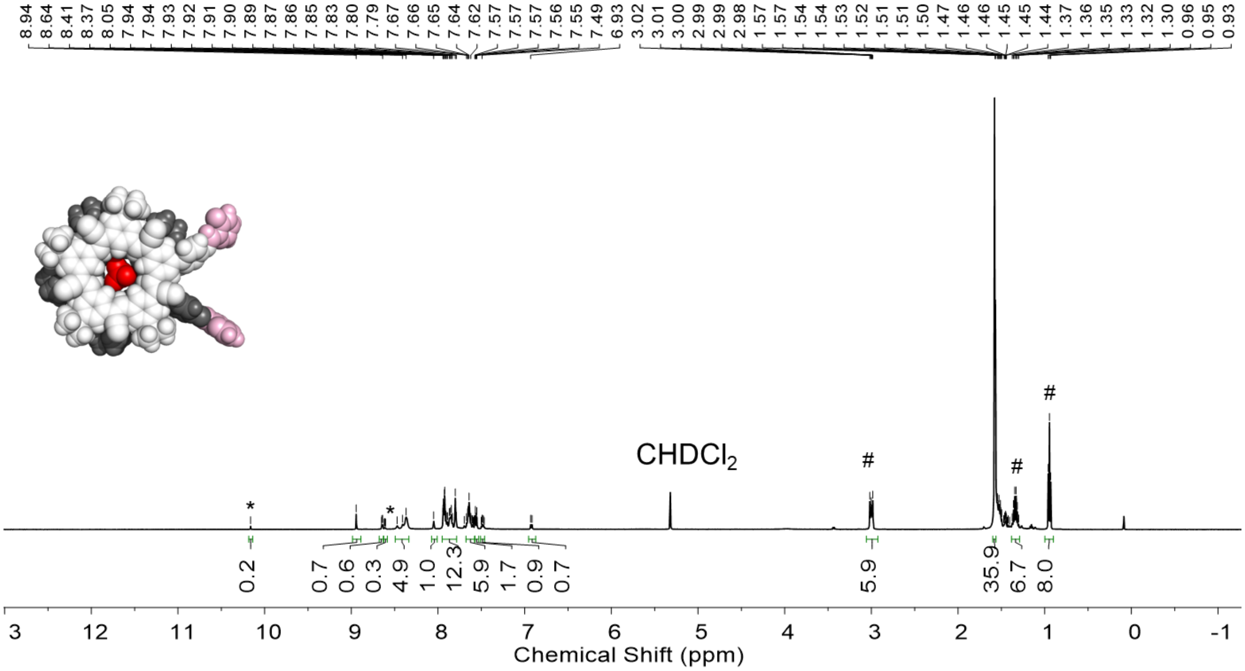

Figure S12. ^{1}H NMR of [**CS**-BF_4-**CS**]$^-$ (500 MHz, CD_2Cl_2, rt). # = TBA^+, ∗ = **F-PyCHO**

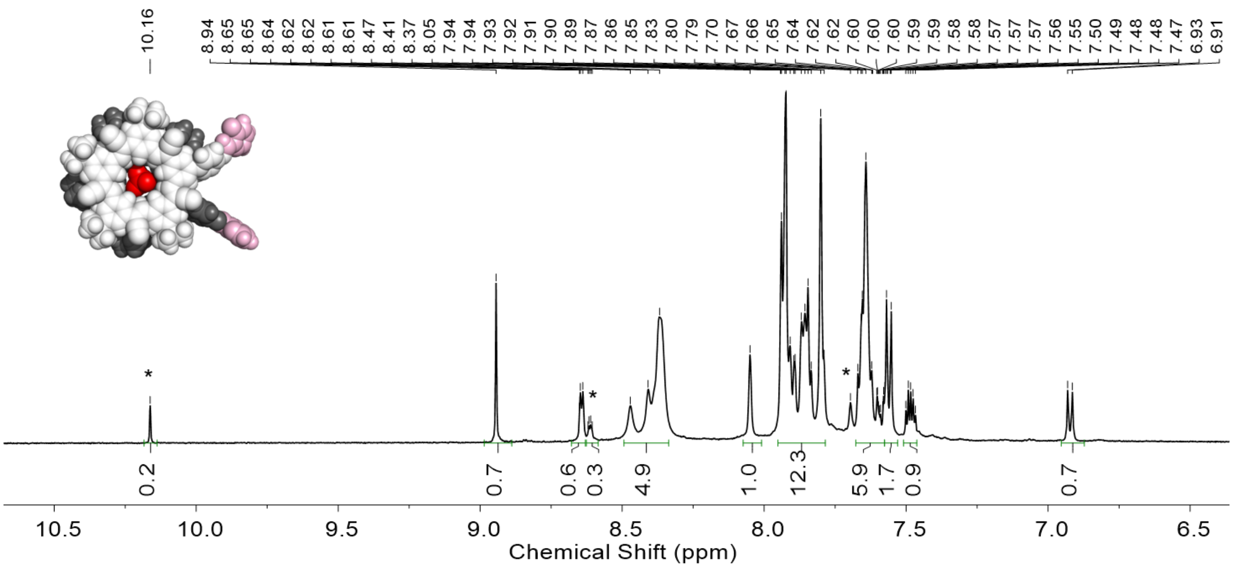

Figure S13. The aromatic region of ^{1}H NMR (Figure S12) of [**CS**-BF_4-**CS**]$^{-}$ (500 MHz, CD_2Cl_2, rt). ∗ = **F-PyCHO**

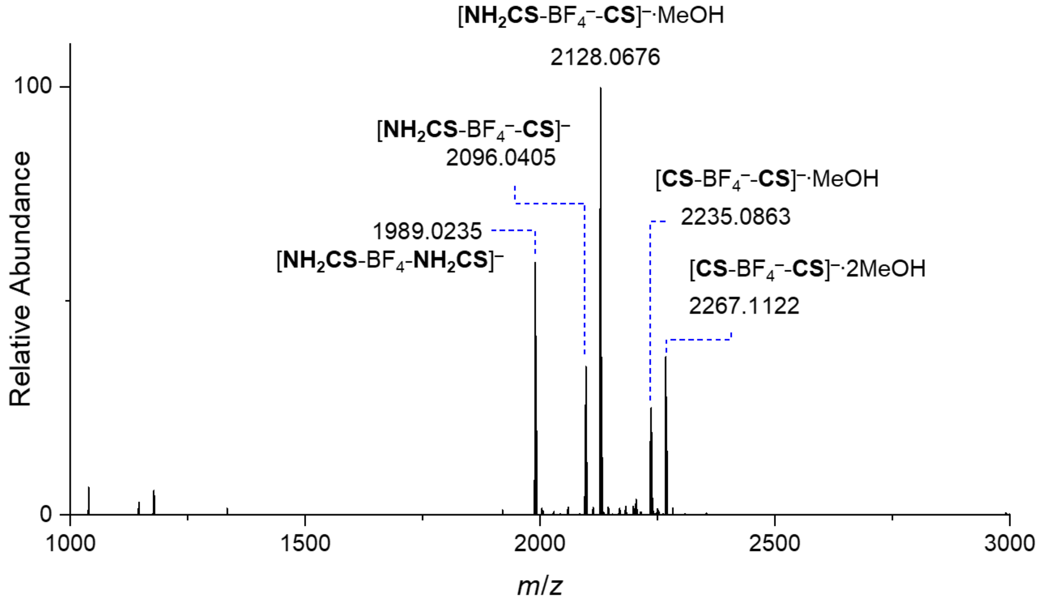

Figure S14. ESI-MS data of [**CS**-BF_4-**CS**]$^{-}$ (dichloromethane, 2 mM).

7. Self-assembly of [**POP**Cu-**CS**-BF_4-**CS**-Cu**POP**]$^{+}$ by Anion-Cation Pathway

In a 20-dram glass vial, TBA [**CS**-BF_4-**CS**] (3.2 mg, 0.0013 mmol, 1 equivalent) and the phosphine-chelated copper salt **POP**Cu·BAr^{F}_4 (2.0 mg, 0.0026 mmol, 2 equivalent) were taken and 1 mL dichloromethane and 10 μL acetonitrile was added in the vial. A red solution was obtained. The solvents were evaporated by blowing argon, and the resulting red solid washed with

Et_2O to obtain [**POP**Cu-**CS**-BF_4-**CS**-Cu**POP**]·BAr^F_4 (5.0 mg, 0.0012 mmol, 92 %). The integration of ^1H NMR peaks are normalized to one cyanostar in assembly.

^1H NMR (400 MHz, CD_2Cl_2, rt): δ 9.01 (s, 1H), 8.36 (br s, 5H), 8.13-8.08 (m, 2H), 8.03-7.58 (m, 24H), 7.58-6.92 (m, 28H), 6.88-6.83 (m, 2H), 1.62-1.48 (m, 42H, overlapping with H_2O peak at 1.53 ppm). ^{19}F NMR (376 MHz, CD_2Cl_2, rt): −122.50, −148.30. HRMS (ESI) *m/z*: [**POP**Cu-**CS**-BF_4-**CS**-Cu**POP**]$^+$ Calcd for $C_{218}H_{184}BCu_2F_6N_{14}O_2P_4^+$ 3407.2328; Found 3407.2260.

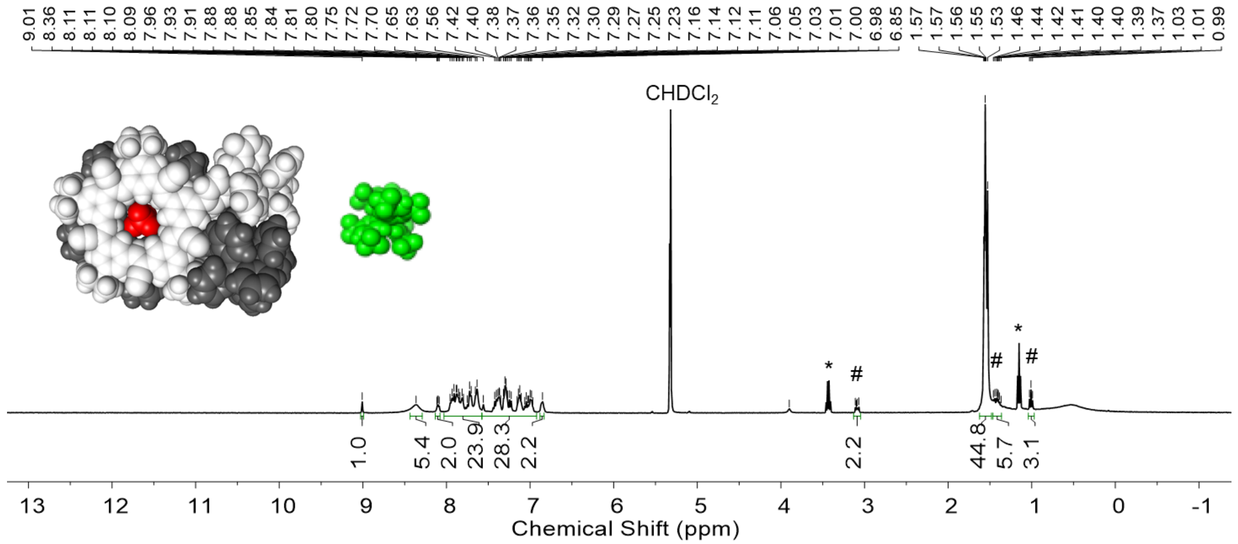

Figure S15. ^1H NMR of [**POP**Cu-**CS**-BF_4-**CS**-Cu**POP**]·BAr^F_4 (400 MHz, CD_2Cl_2, rt). * = Et_2O, # = TBA^+.

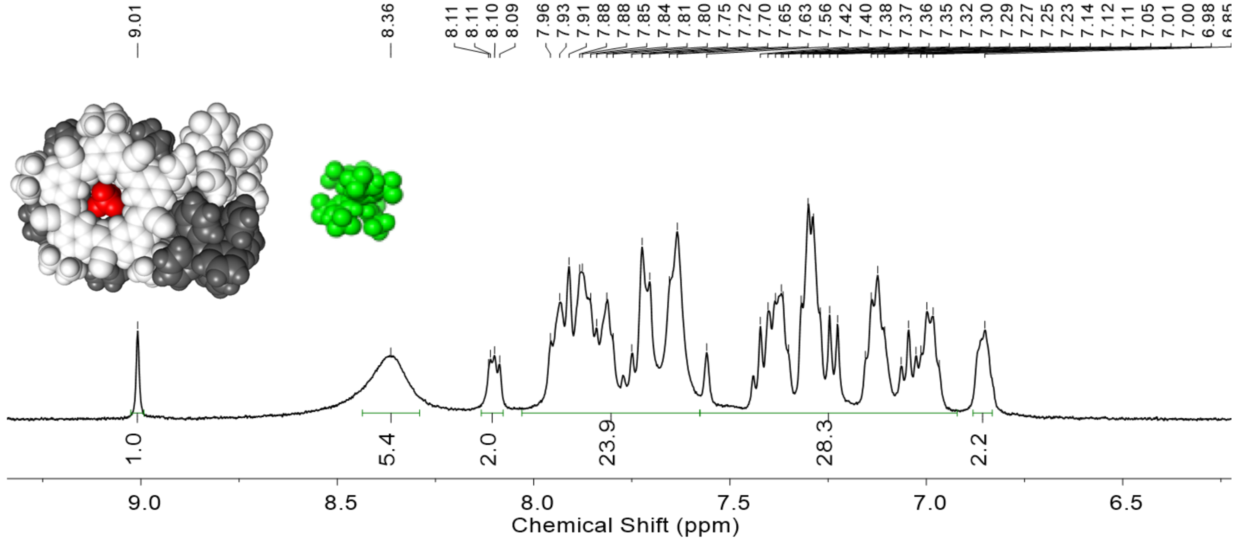

Figure S16. The aromatic region of ^1H NMR (Figure S15) of [**POP**Cu-**CS**-BF_4-**CS**-Cu**POP**]·BAr^F_4 (400 MHz, CD_2Cl_2, rt).

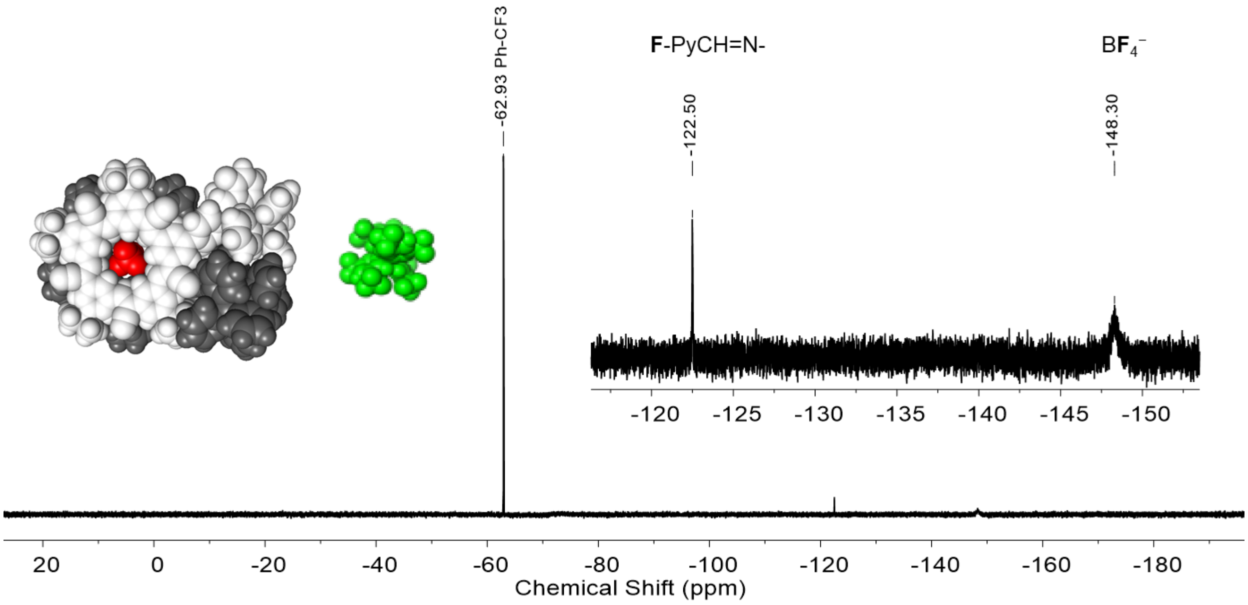

Figure S17. ^{19}F NMR of [**POP**Cu-**CS**-BF_4-**CS**-Cu**POP**]·BAr^F_4 (376 MHz, CD_2Cl_2, rt). The peaks corresponding to the BAr^F_4 anion overlaps with the reference peak of Ph-CF_3.

8. Self-assembly of [Ph$_3$PAu-CS]·NTf_2

NH_2-CS (7.2 mg, 0.0076 mmol, 1 equivalent), **F-PyCHO** (1.5 mg, 0.0114 mmoL, 1.5 equivalent) and the triphenylphosphine gold triflimide salt [**Ph$_3$P**Au]·**NTf_2** (5.6 mg, 0.0076 mmol, 1 equivalent) were taken in a 20-dram glass vial and 1.0 mL dichloromethane was added to the vial. To the yellow solution, 10 µL acetonitrile was added. The solvents were evaporated by blowing argon. The resulting yellow precipitate was washed with Et_2O (3×15 mL) to get pure [**Ph$_3$P**Au-**CS**]·NTf_2 (12.3 mg, 0.0068 mmol, 89 %).
^{1}H NMR (500 MHz, CD_2Cl_2, rt): δ 9.53 (s, 1H), 8.63 (s, 1H), 8.58-8.48 (m, 4H), 8.07-7.52 (m, 37H), 1.58-1.44 (m, 36H, overlapping with H_2O peak at 1.53 ppm). ^{19}F NMR (376 MHz, CD_2Cl_2, rt): −78.74, −121.22. HRMS (ESI) *m/z*: [**Ph$_3$P**Au-**CS**]$^+$ Calcd for $C_{91}H_{79}AuFN_7P^+$ 1516.5779; Found 1517.5831.

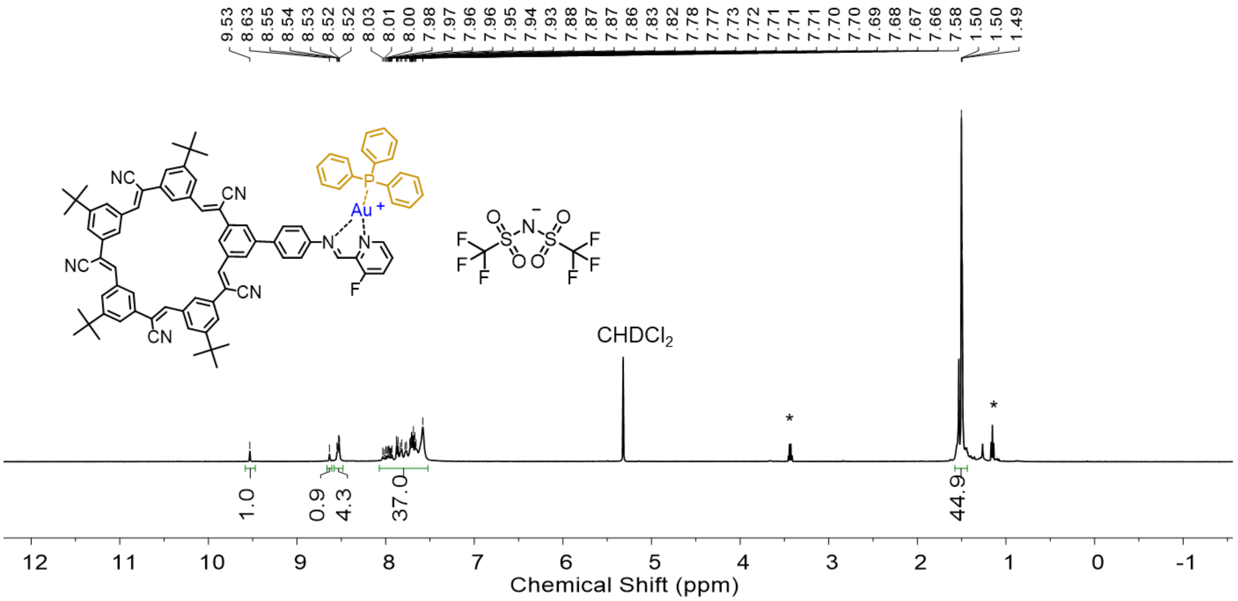

Figure S18. ^{1}H NMR of [**Ph_3P**Au-**CS**]·NTf_2 (500 MHz, CD_2Cl_2, rt). * = Et_2O

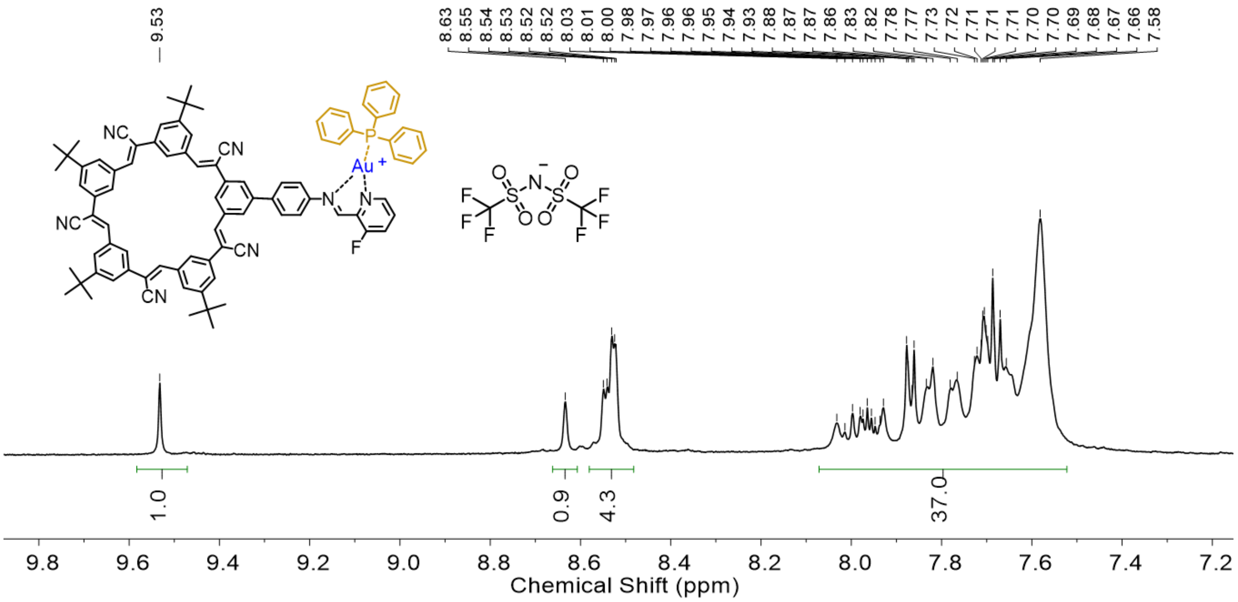

Figure S19. The aromatic region of ^{1}H NMR (Figure S18) of [**Ph_3P**Au-**CS**]·NTf_2 (500 MHz, CD_2Cl_2, rt).

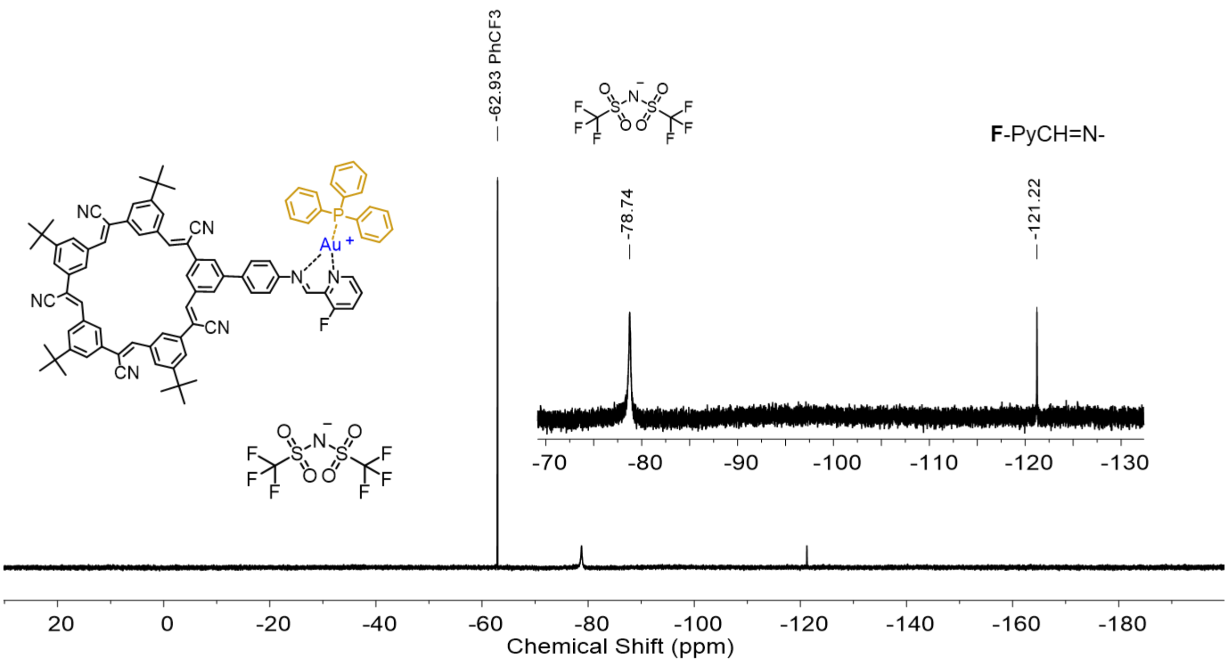

Figure S20. ^{19}F NMR of [**Ph$_3$P**Au-**CS**]·NTf$_2$ (376 MHz, CD_2Cl_2, rt).

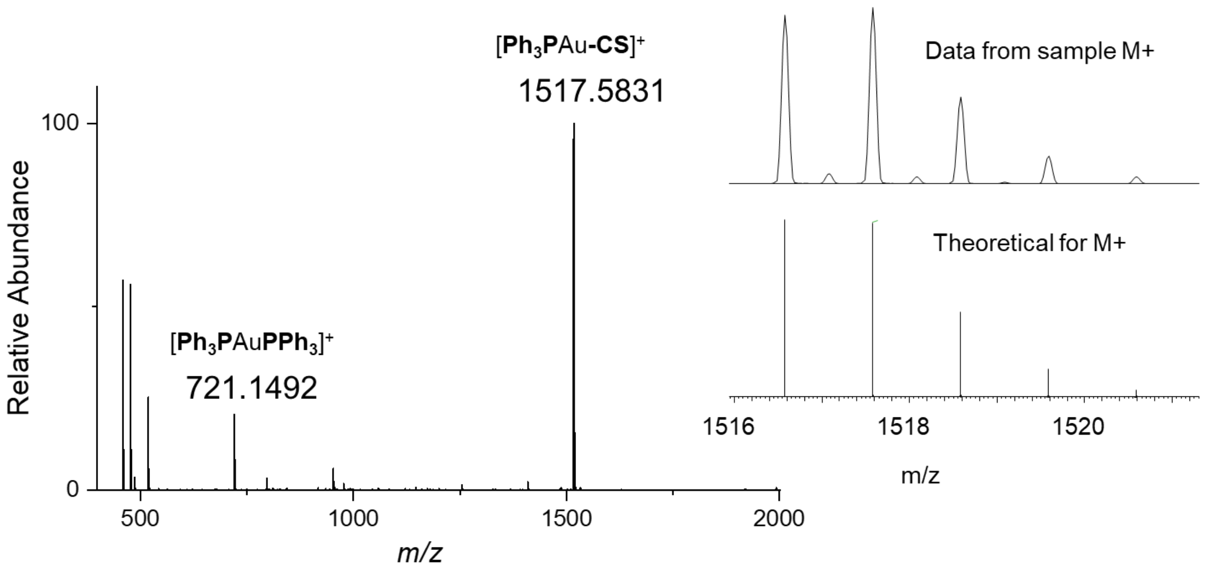

Figure S21. ESI-MS spectrum of [**Ph$_3$P**Au-**CS**]·NTf$_2$

9. Self-assembly of [**PPh$_3$**Au-**CS**-BF$_4$-**CS**-Au**PPh$_3$**]$^+$ by One-pot

NH$_2$-CS (6.4 mg, 0.0067 mmol, 1 equivalent), **F-PyCHO** (1.6 mg, 0.0134 mmol, 2 equivalents) and [**PPh$_3$**Au]·BF$_4$ (3.7 mg, 0.0067 mmol, 1 equivalent) were taken in a 20-dram glass vial and 1.0 mL dichloromethane was added to the vial. To the yellow solution, 10 µL acetonitrile was added. The solvents were evaporated by blowing argon. The resulting yellow precipitate was washed with Et$_2$O (3×10 mL) to get pure [**PPh$_3$**Au-**CS**-BF$_4$-**CS**-Au**PPh$_3$**]·BF$_4$ (14 mg, 0.0043 mmol, 79 %). The integration of ^{1}H NMR peaks are normalized to one cyanostar in assembly. ^{1}H NMR (500 MHz, CD$_2$Cl$_2$, rt): δ 9.56 (s, 1H), 8.65-8.21 (m, 5H), 8.15-7.44 (m, 40H), 1.64-1.50 (m, 33H, overlapping with H$_2$O peak at 1.53 ppm). ^{19}F NMR (376 MHz, CD$_2$Cl$_2$, rt): −121.16, −149.73. HRMS (ESI) *m/z*: [**PPh$_3$**Au-**CS**-BF$_4$-**CS**-Au**PPh$_3$**]$^+$ Calcd for $C_{182}H_{158}N_{14}Au_2BF_6P_2^+$ 3120.1592; Found 3120.1632.

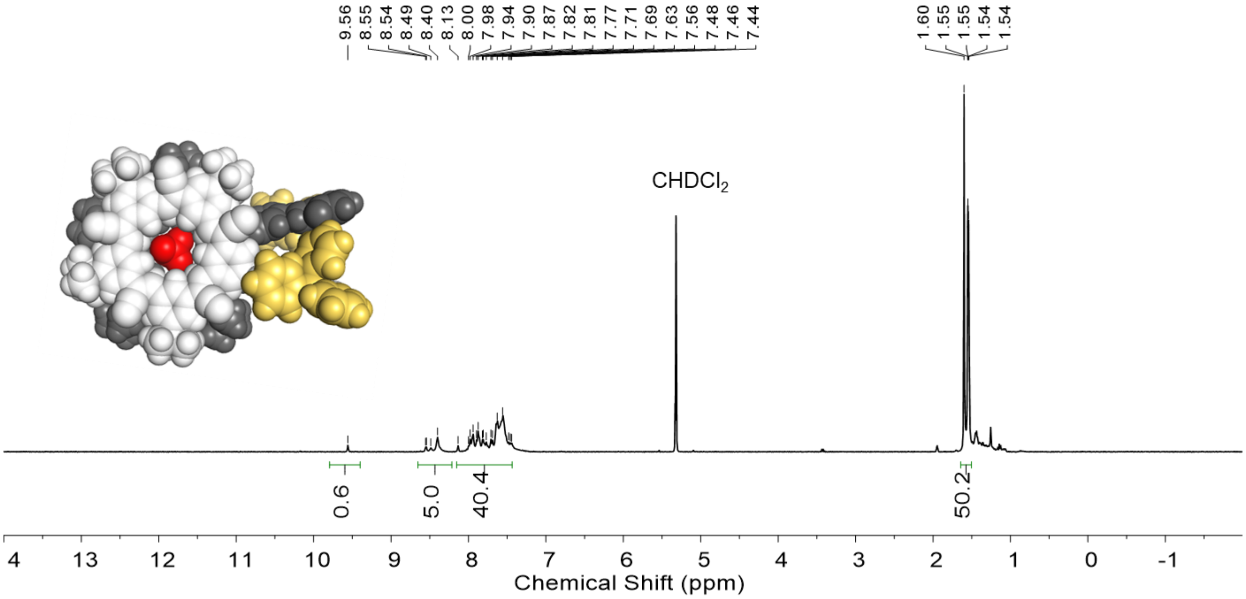

Figure S22. ^{1}H NMR of [**PPh$_3$**Au-**CS**-BF$_4$-**CS**-Au**PPh$_3$**]·BF$_4$ (500 MHz, CD$_2$Cl$_2$, rt).

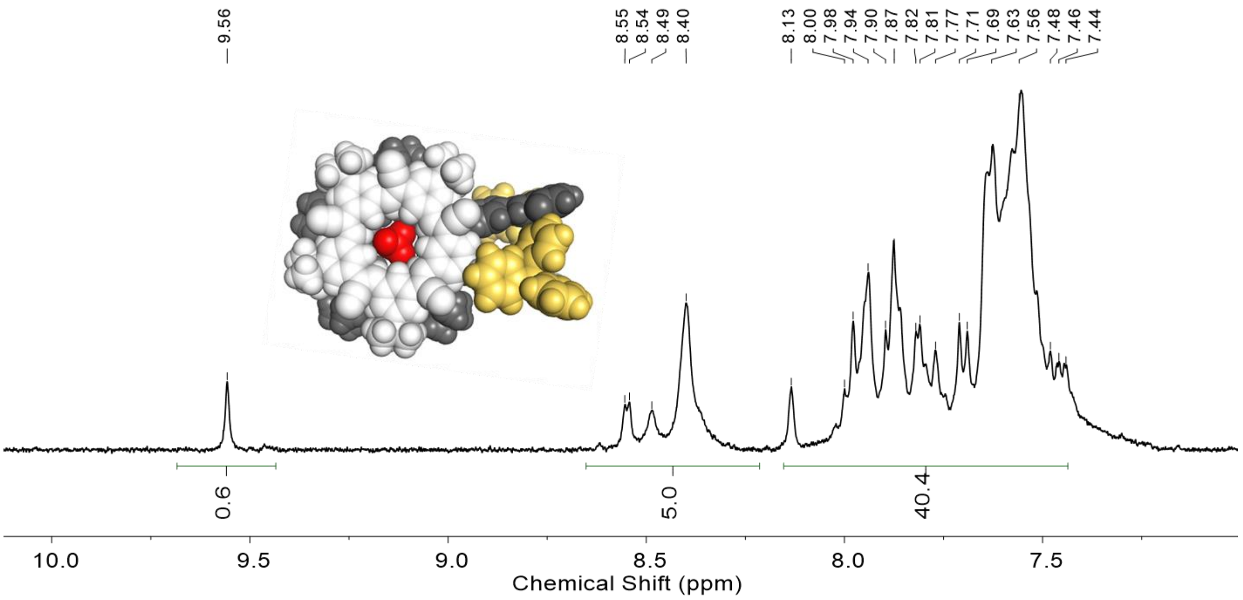

Figure S23. The aromatic region of ^{1}H NMR (Figure S22) of [**PPh**$_3$Au-**CS**-BF$_4$-**CS**-Au**PPh**$_3$]·BF$_4$ (500 MHz, CD_2Cl_2, rt).

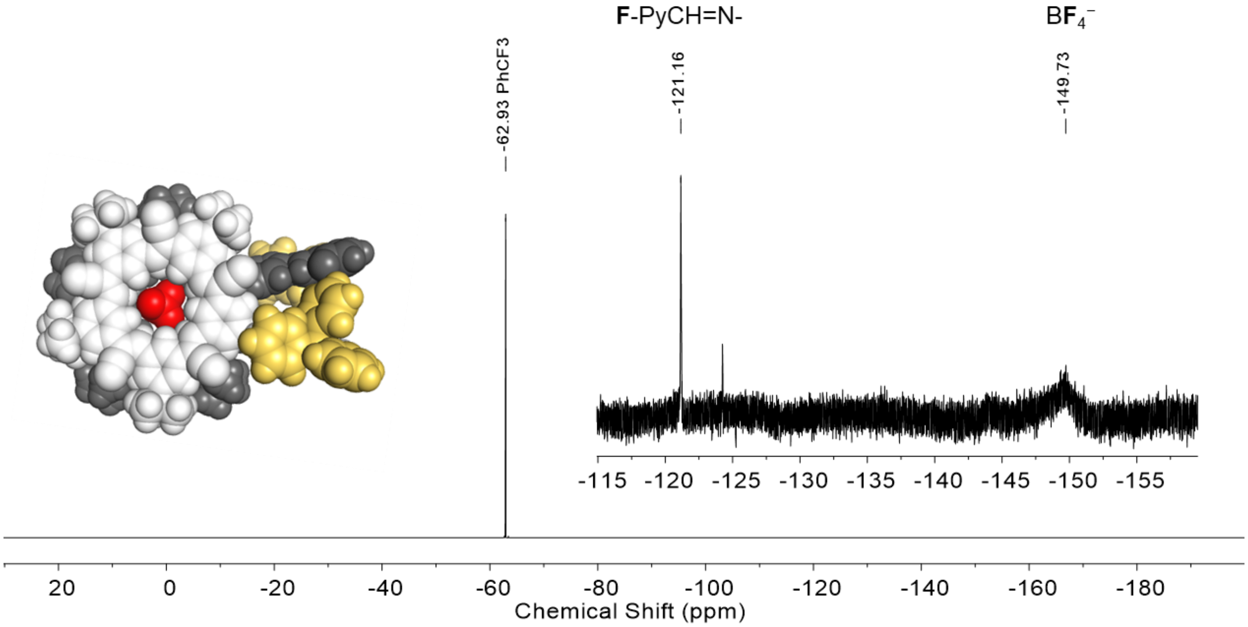

Figure S24. ^{19}F NMR of [**PPh**$_3$Au-**CS**-BF$_4$-**CS**-Au**PPh**$_3$]·BF$_4$ (376 MHz, CD_2Cl_2, rt).

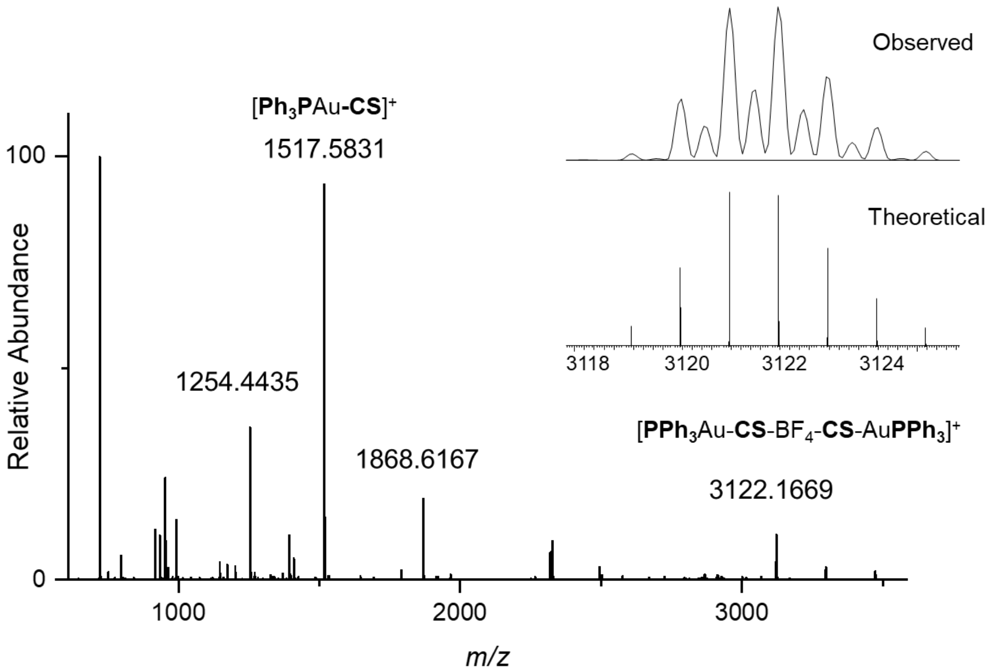

Figure S25. ESI-MS spectrum of [**PPh**$_3$Au-**CS**-BF_4-**CS**-Au**PPh**$_3$]·BF_4.

10. Titration of [**Ph**$_3$**P**Au-**CS**]·NTf_2 with TBABF$_4$

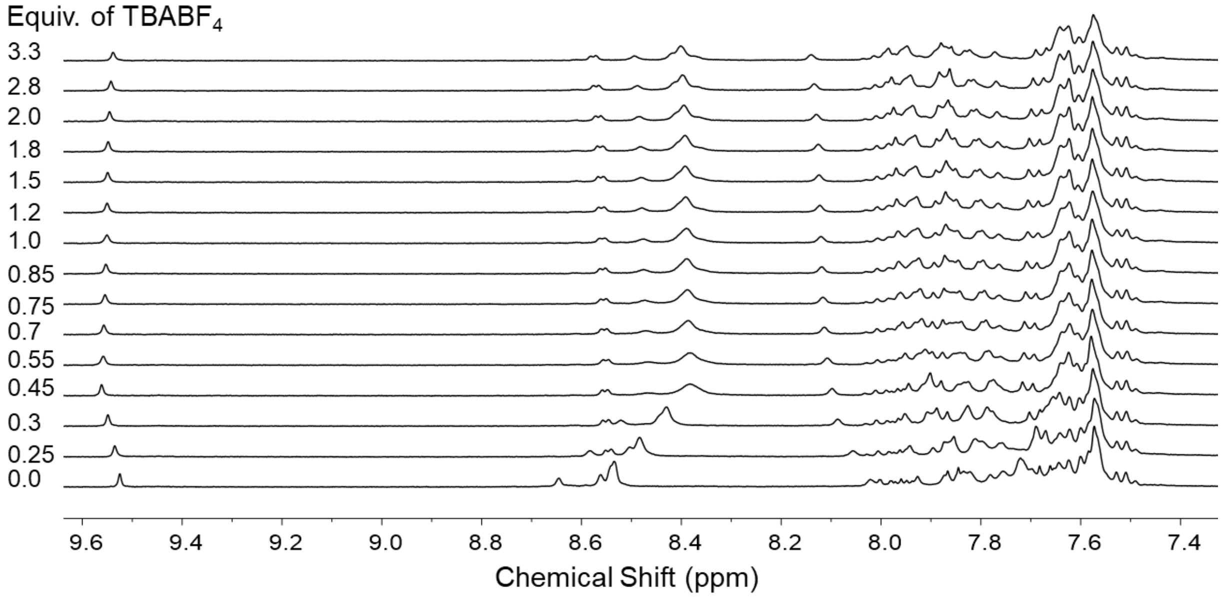

Figure S26. ^{1}H NMR spectra for the titration of [**Ph**$_3$**P**Au-**CS**]·NTf_2 (the bottom spectrum, 3.0 mM) (the bottom spectrum) with TBABF$_4$ (with increasing equivalents from bottom to top: 0.0, 0.25, 0.3, 0.45, 0.55, 0.7, 0.75, 0.85, 1.0, 1.2, 1.5, 1.8, 2.0, 2.8, 3.3) (500 MHz, CD_2Cl_2, rt). [Note: The equivalents of TBABF$_4$ added was verified by the integration ratio of imine proton with H_α of TBA^+ of TBABF$_4$ salt]

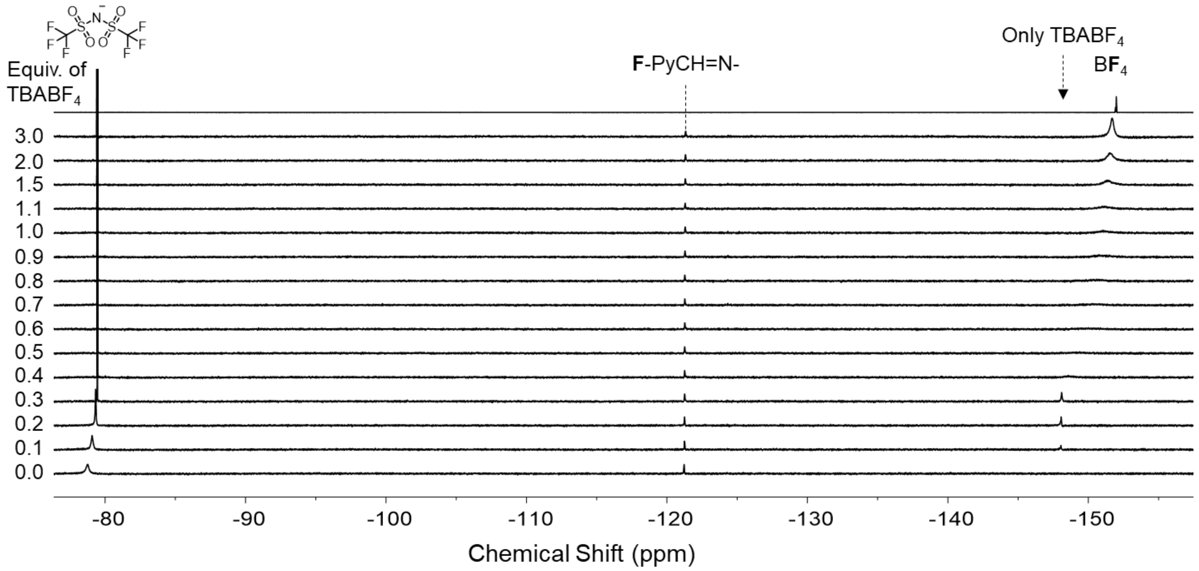

Figure S27. ^{19}F NMR spectra (376 MHz, CD_2Cl_2, rt) for the titration of [**Ph$_3$P**Au-**CS**]·NTf$_2$ (the bottom spectrum, 3.0 mM) with TBABF$_4$ (with increasing equivalents from bottom to top: 0, 0.1, 0.2, 0.3, 0.4, 0.5, 0.6, 0.7, 0.8, 0.9, 1.0, 1.1, 1.5, 2.0, 3.0) and the top spectrum for only TBABF$_4$.

11. Self-assembly of [PPh$_3$Au-CS-BF$_4$-CS-AuPPh$_3$]$^+$ by Cation-Anion Pathway

After the above titration, the final solution, which contained the [**Ph$_3$P**Au-**CS**]·NTf$_2$ (3.3 mg, 0.0018 mmol, 1 equivalent) and TBABF$_4$ (0.6 mg, 0.0018 mmol, 3.3 equivalent), was transferred in a 20-dram glass vial from the NMR tube. The solvents were then evaporated by blowing argon, and a yellow precipitate was obtained. The precipitate was then washed with Et_2O (3×10 mL) to obtain [**PPh$_3$**Au-**CS**-BF$_4$-**CS**-Au**PPh$_3$**]·X$^-$ (X$^-$ = BF$_4$ / NTf$_2^-$) (3.7 mg, 0.0011 mmol, 61 %). The integration of ^{1}H NMR peaks are normalized to one cyanostar in assembly.
^{1}H NMR (600 MHz, CD_2Cl_2, rt): δ 9.54 (s, 1H), 8.61-8.34 (m, 8H), 8.16 (s, 1H), 8.04-7.42 (m, 56H), 1.57-1.53 (m, 25H, overlaps with H_2O). ^{19}F NMR (376 MHz, CD_2Cl_2, rt): −79.43, −121.29, −151.54. HRMS (ESI) *m/z*: [**PPh$_3$**Au-**CS**-BF$_4$-**CS**-Au**PPh$_3$**]$^+$ Calcd for $C_{182}H_{158}N_{14}Au_2BF_6P_2^+$ 3120.1592; Found 3120.1543.

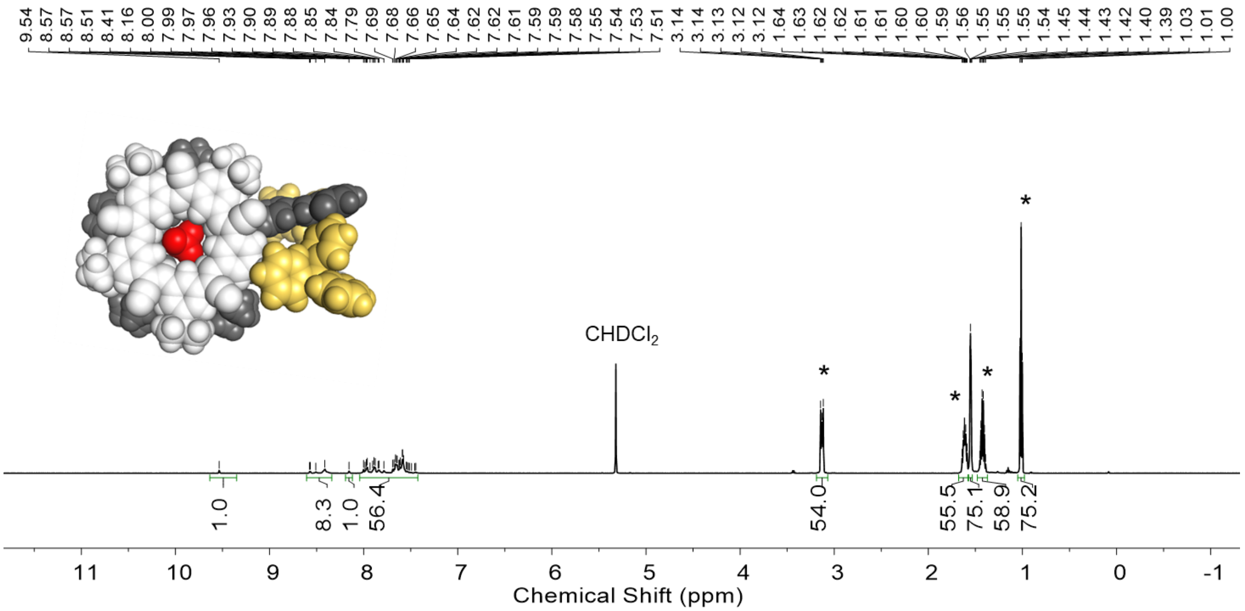

Figure S28. ^{1}H NMR (600 MHz, CD_2Cl_2, rt) of [**PPh$_3$**Au-**CS**-BF_4-**CS**-Au**PPh$_3$**]$^{+}$ formed by cation-anion pathway.* = TBA^{+}

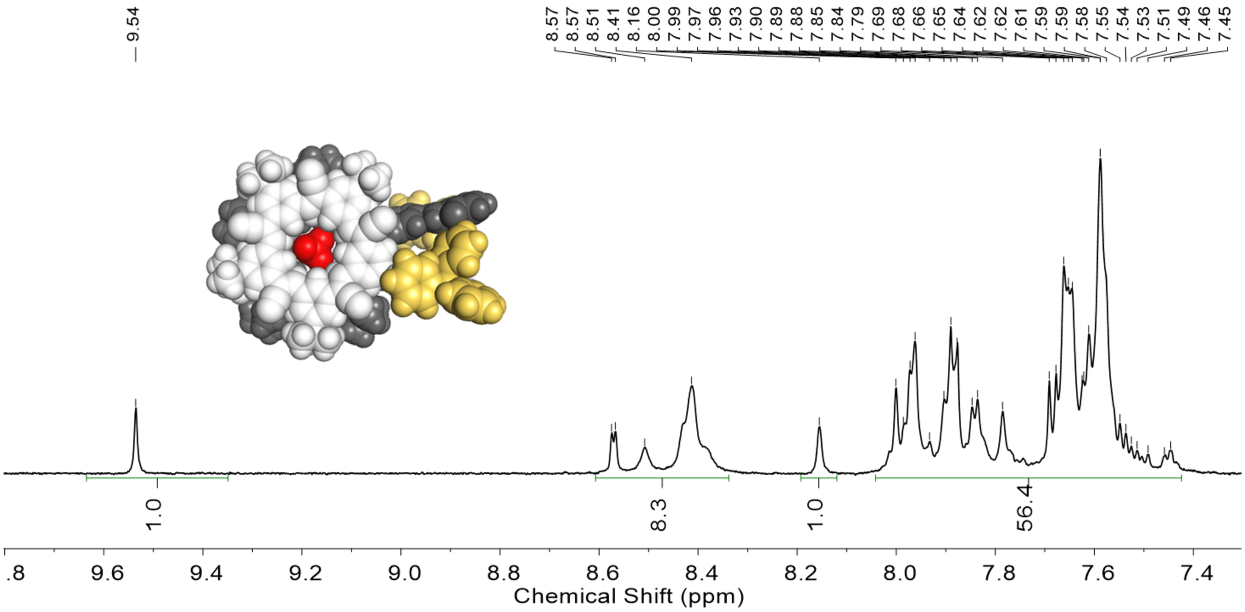

Figure S29. The aromatic region of ^{1}H NMR (Figure S28) of [**PPh$_3$**Au-**CS**-BF_4-**CS**-Au**PPh$_3$**]$^{+}$ formed by cation-anion pathway (600 MHz, CD_2Cl_2, rt).

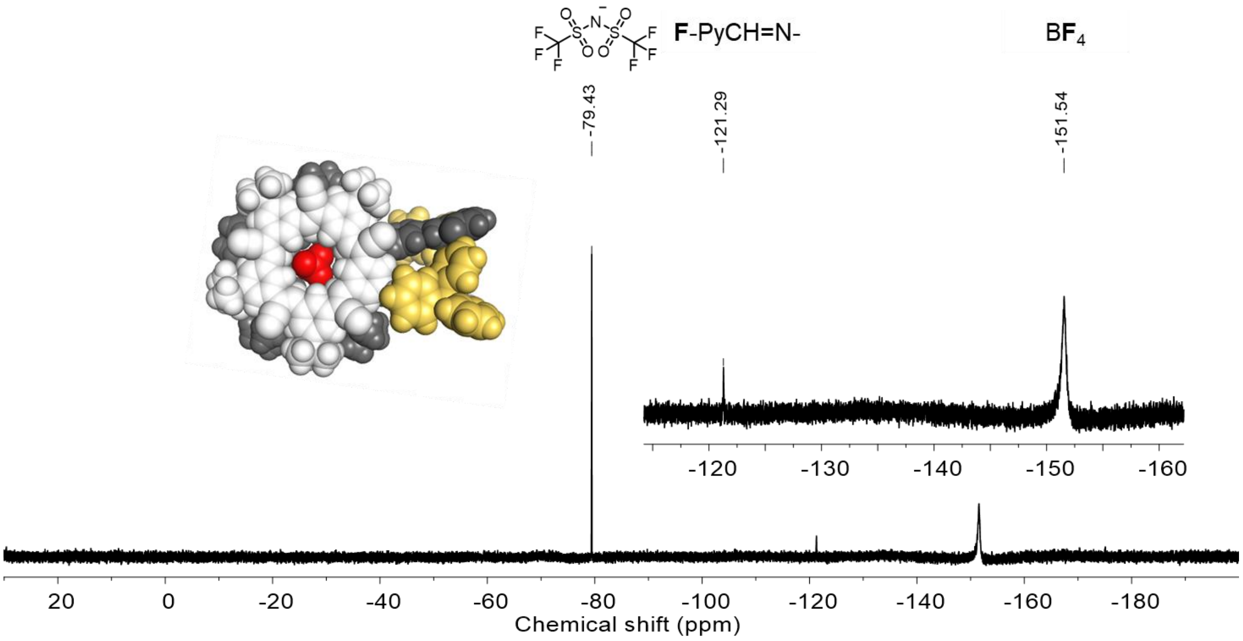

Figure S30. ^{19}F NMR (376 MHz, CD_2Cl_2, rt) of of [**PPh₃**Au-**CS**-BF_4-**CS**-Au**PPh₃**]$^+$ formed by cation-anion pathway. No additional reference like Ph-CF_3 was added in the NMR tube for collecting this spectrum.

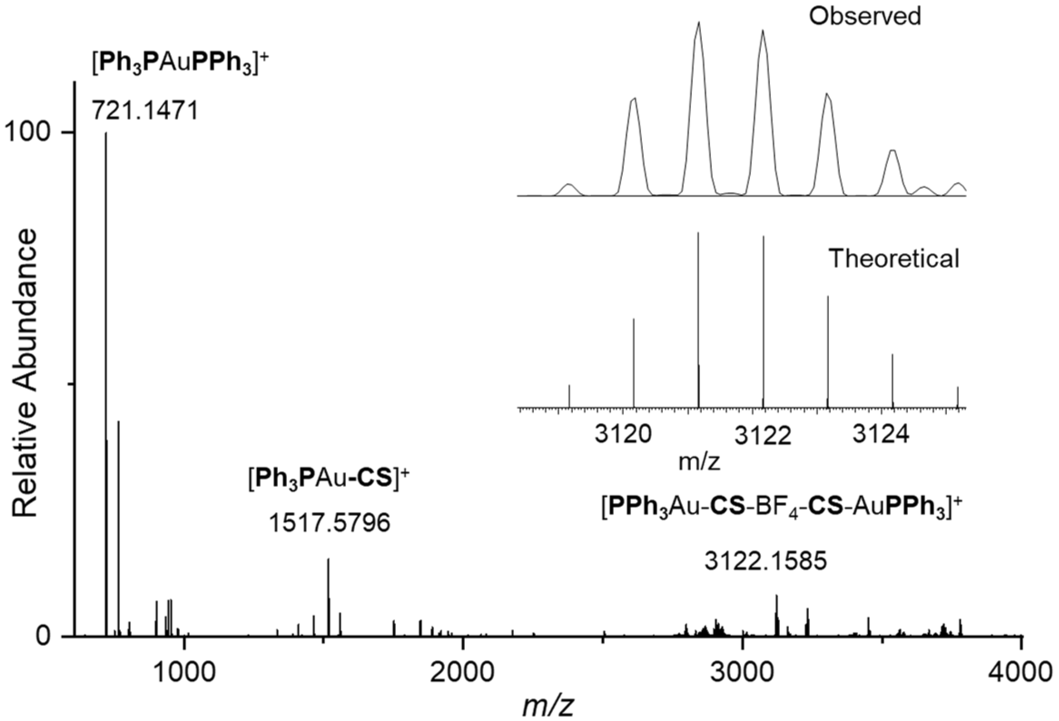

Figure S31. ESI-MS spectrum of [**PPh₃**Au-**CS**-BF_4-**CS**-Au**PPh₃**]$^+$ formed by cation-anion pathway.

12. Self-assembly of Cyanostar-based Gold(I) Assembly by Anion-Cation Pathway

In a 20-dram glass vial, the TBA^+[**CS**-BF_4-**CS**]$^-$ (3.0 mg, 0.0012 mmol, 1 equivalent), and the triphenylphosphine gold triflimide salt the [**PPh$_3$**Au]·NTf_2 (1.8 mg, 0.0024 mmol, 2 equivalents) were taken and 1 mL dichloromethane and 10 µL acetonitrile was added in the vial. A yellow solution was obtained. The solvents were then evaporated by blowing argon, and the resulting solid was washed with Et_2O and subjected to characterization. [**PPh$_3$**Au-**CS**-BF_4-**CS**-Au**PPh$_3$**]·NTf_2 was not successfully formed.

We considered that the Au^+ ions may form a bridged complex based on similar work by Berry, Olmsted, and Balch,[10] but the high-resolution mass spectrometry (Figure S35) was not consistent. The HR-ES shows three peaks at 721.1489 *m/z*, 1,147.0542 *m/z* and 1,409.1545 *m/z* as singly positive ions. No major peaks are observed beyond 2000 *m/z*. A daughter assembly [**PPh$_3$**Au-**CS]$^+$** has a theoretical *m/z* of 1516.5779 *m/z*, the target assembly, [**PPh$_3$**Au-**CS**-BF_4-**CS**-Au**PPh$_3$**]$^+$, has a theoretical *m/z* of 3120.1592, and a gold bridged assembly has a theoretical a *m/z* of 2858.0686. As a result, we likely have a mixture of assemblies in solution and cannot make any conclusive statements as to the nature of the complex formed by the anion-cation pathway only other than to say that it is different than the one-pot and cation-anion assemblies.

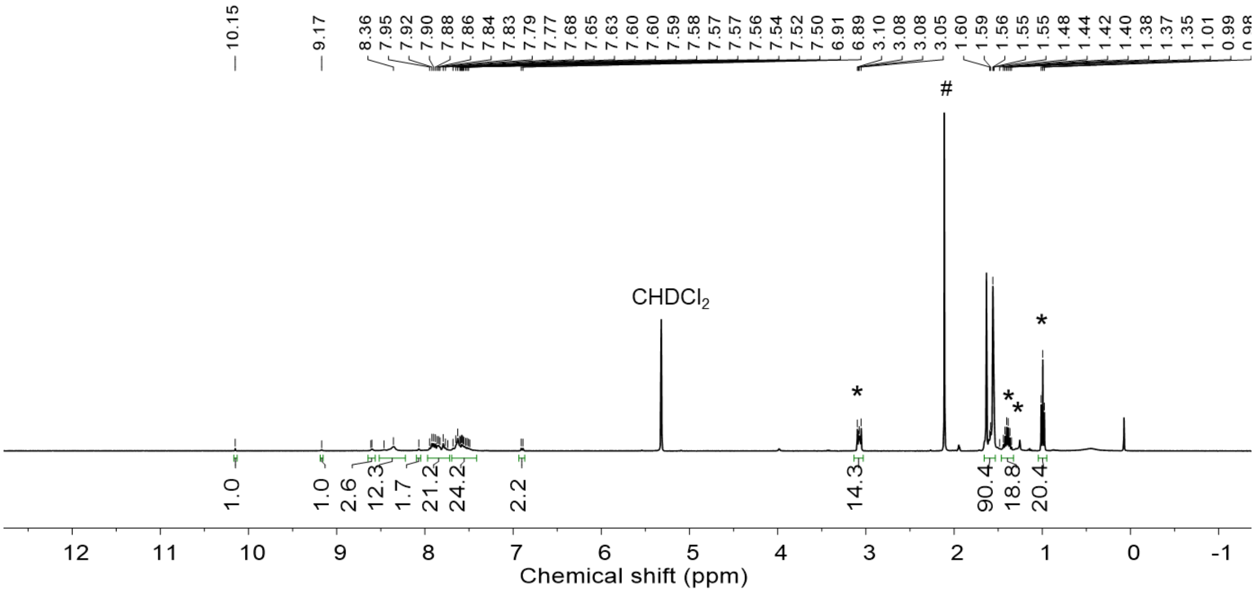

Figure S32. ^1H NMR (500 MHz, CD_2Cl_2, rt) of the gold assembly formed by anion-cation pathway. * = TBA^+, # = acetone

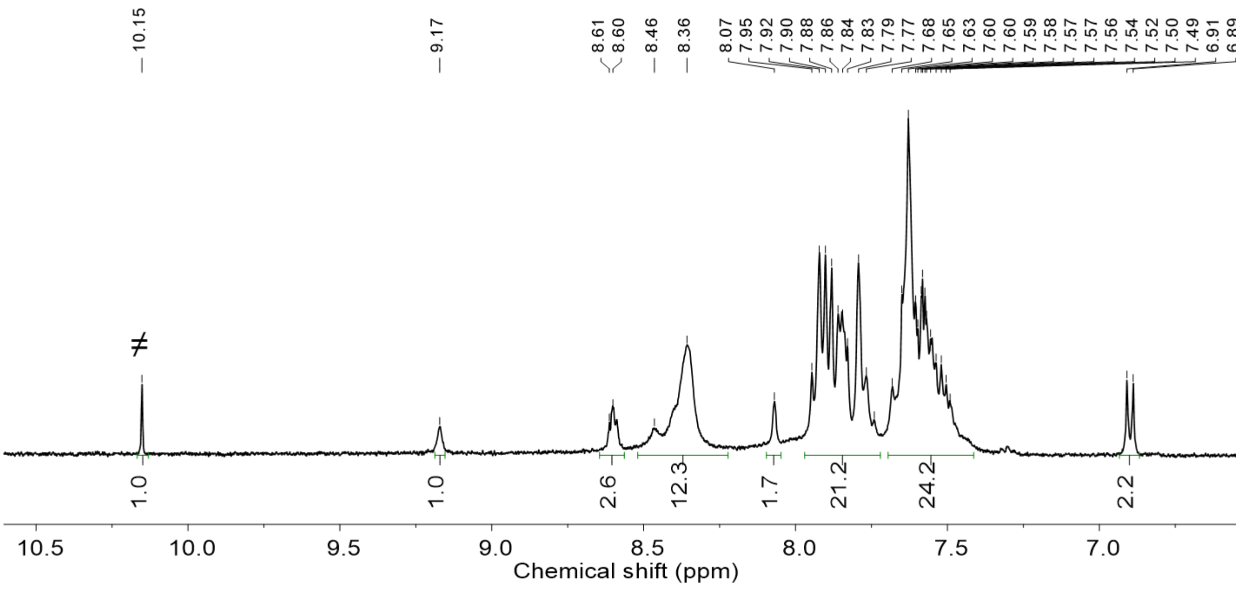

Figure S33. The aromatic region of ^{1}H NMR (Figure S32) of the gold assembly (500 MHz, CD_2Cl_2, rt). ≠ = **F-PyCHO**

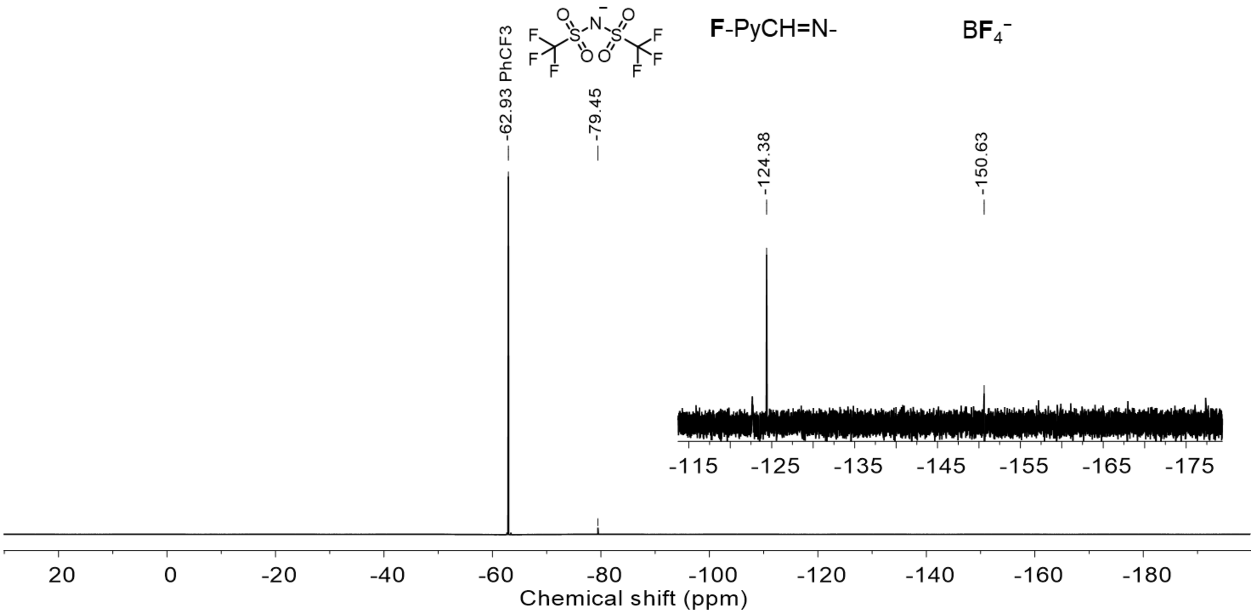

Figure S34. ^{19}F NMR (376 MHz, CD_2Cl_2, rt) of the gold assembly formed by anion-cation pathway.

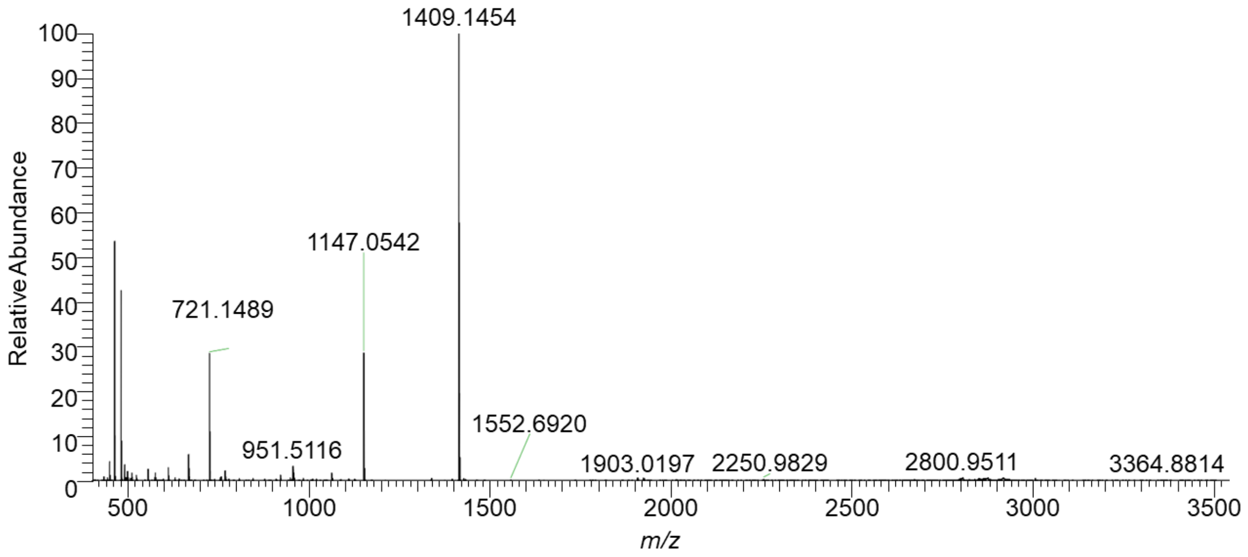

Figure S35. ESI-MS spectrum of the solution at the end of the anion-cation pathway formed using BF_4^- and [**PPh$_3$**Au]·NTf_2.

13. Self-assembly of [**CS**-BF_4-**CS**]$^-$ in Presence of Excess TBABF$_4$

NH$_2$-CS (3.5 mg, 0.004 mmol, 1 equivalent), **F-PyCHO** (0.46 mg, 0.004 mmol, 1 equivalent) and TBABF$_4$ (6.6 mg, 0.020 mmol, 5 equivalent) were taken in a 20-dram glass vial and 1.0 mL dichloromethane was added in the vial. Excess TBABF$_4$ was used in the reaction to ensure that all macrocycles form 2:1 cyanostar:BF_4^- complexes in solution. A yellow solution was obtained. The solvents were evaporated by blowing argon, and the crude oil was dried under vacuum overnight. The crude product (8.6 mg) was directly dissolved in CD_2Cl_2 to take ^{1}H NMR of the assembly.

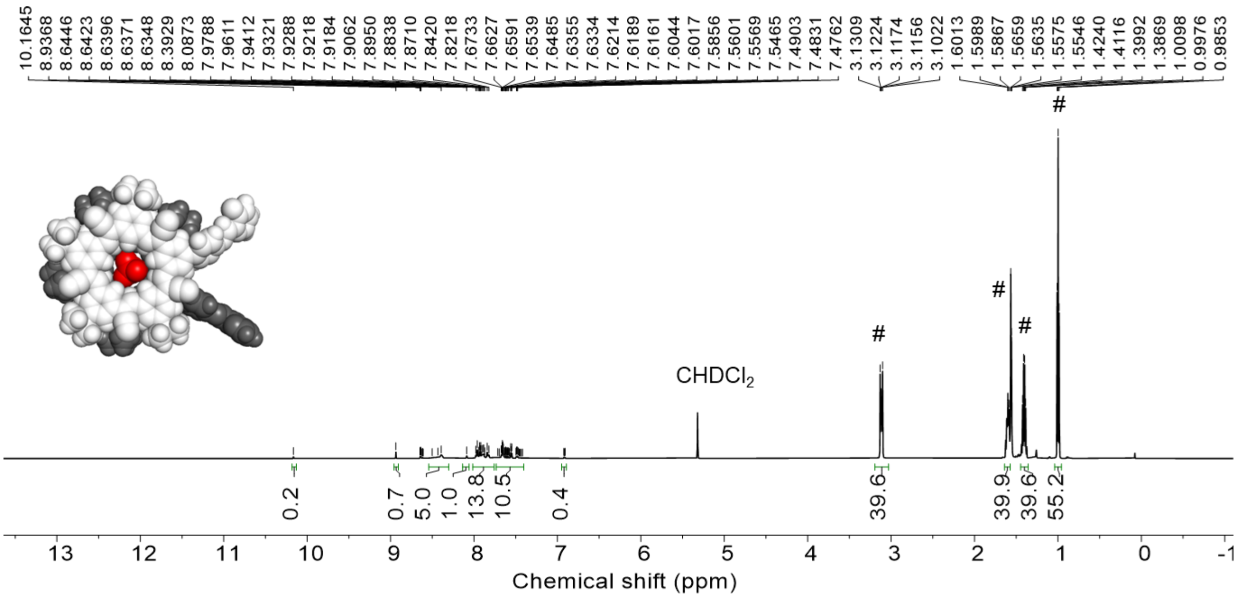

Figure S36. ^{1}H NMR of [**CS**-BF_4-**CS**]$^-$ with excess TBABF$_4$ (500 MHz, CD_2Cl_2, rt). # = TBA^+

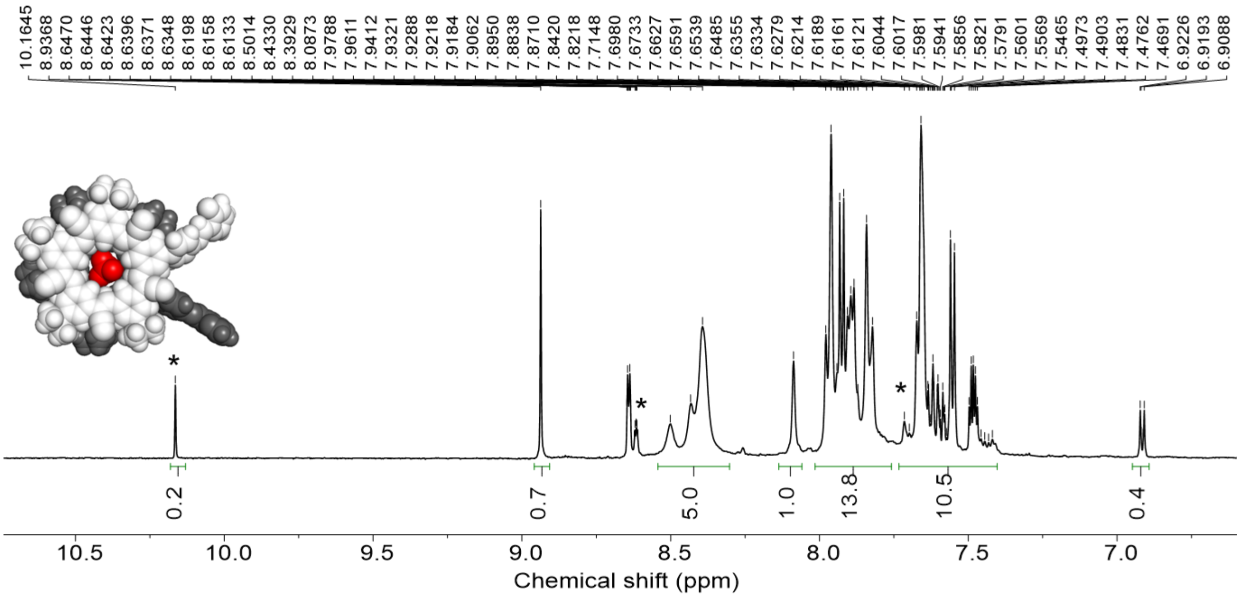

Figure S37. The aromatic region of ^{1}H NMR (Figure S36) of [**CS**-BF_4-**CS**]$^{-}$ (500 MHz, CD_2Cl_2, rt). ∗ = **F-PyCHO**

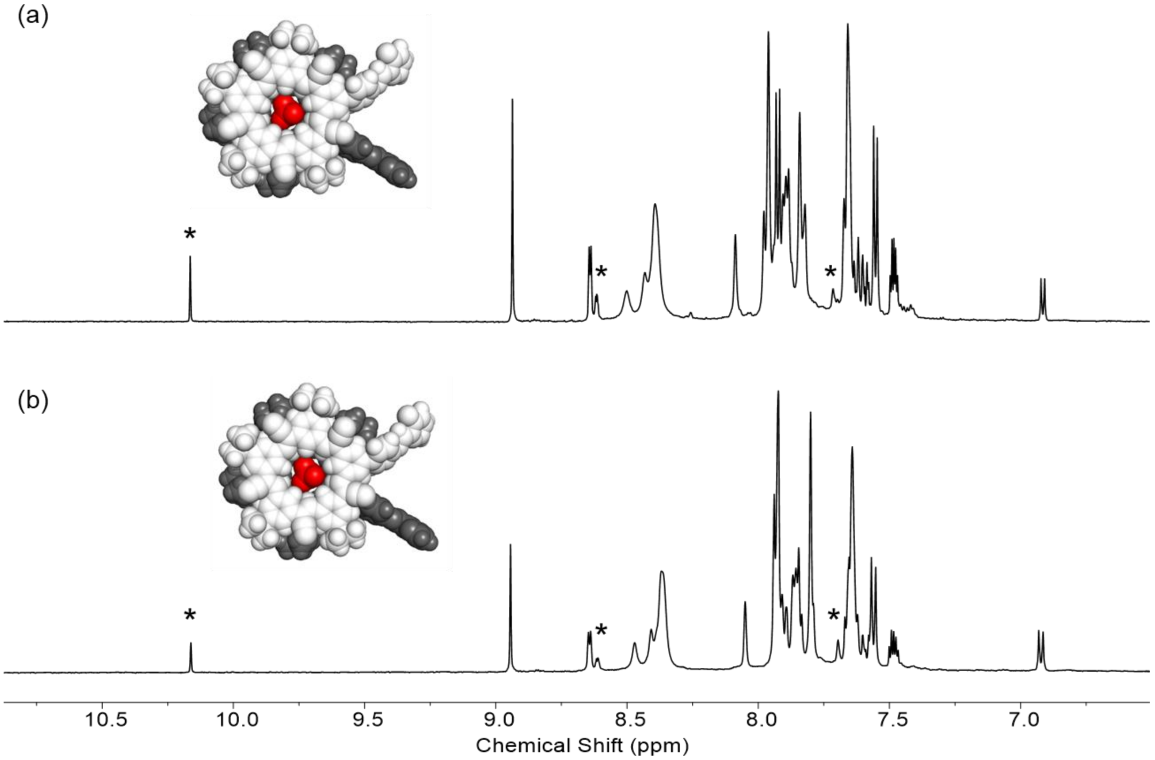

Figure S38. The comparison of the aromatic regions of ^{1}H NMR of [**CS**-BF_4-**CS**]$^{-}$ with (a) five equivalents of $TBABF_4$ and (b) one equivalent of $TBABF_4$ (500 MHz, CD_2Cl_2, rt). ∗ = **F-PyCHO**

14. Self-assembly of Cyanostar-based Gold(I) Assembly by Anion-Cation Pathway in Presence of Excess $TBABF_4$

In a 20-dram glass vial, TBA^+[**CS**-BF_4-**CS**]$^-$ (8.6 mg, 0.0004 mmol based on **CS-NH_2** moles, 1 equivalent), which had excess $TBABF_4$ present, and [**PPh_3**Au]·NTf_2 (0.6 mg, 0.0008 mmol, 2 equivalents) were taken and 1 mL dichloromethane and 10 µL acetonitrile was added in the vial. A yellow solution was obtained. The solvents were then evaporated by blowing argon and the crude oily precipitate was washed with Et_2O (2 mL × 3) and subjected to characterization.

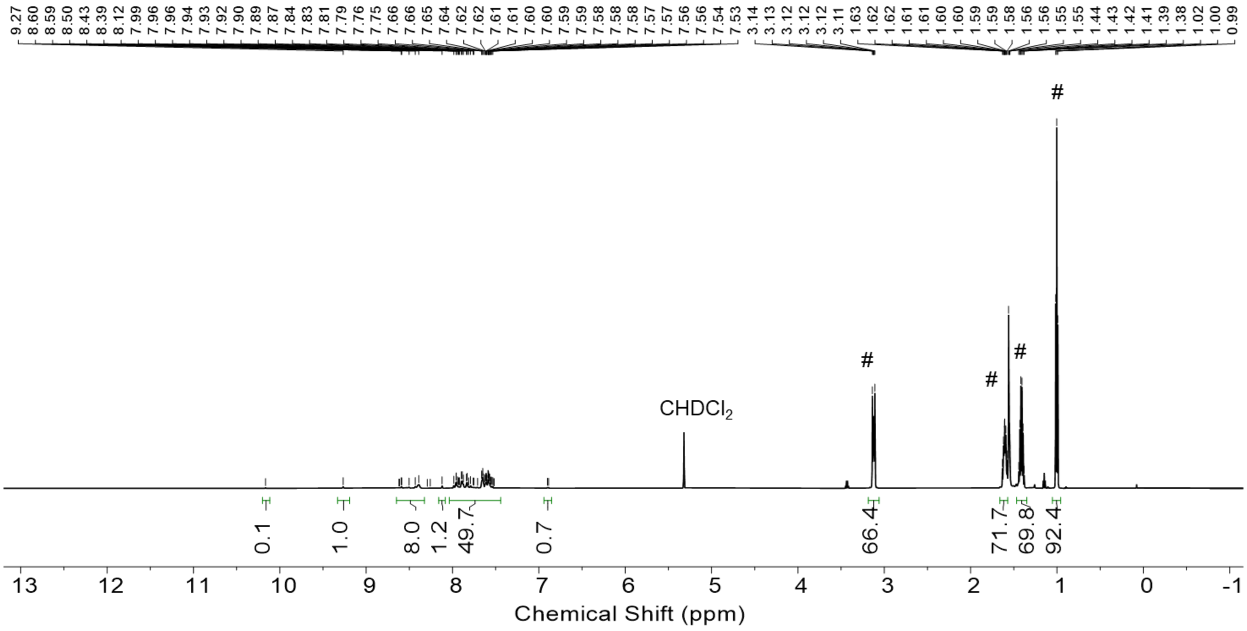

Figure S39. ^1H NMR (500 MHz, CD_2Cl_2, rt) of the gold assembly formed by anion-cation pathway in presence of excess $TBABF_4$. # = TBA^+.

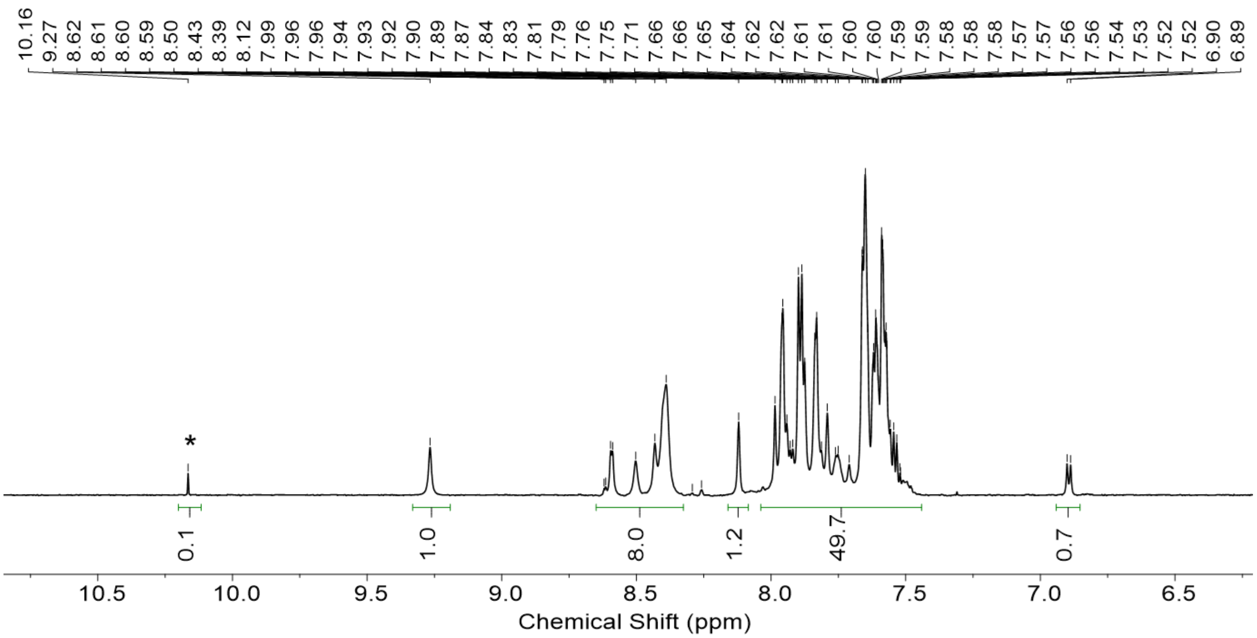

Figure S40. The aromatic region of ^1H NMR (Figure S39) of the gold assembly (500 MHz, CD_2Cl_2, rt) formed by anion-cation pathway in presence of excess $TBABF_4$. * = **F-PyCHO**

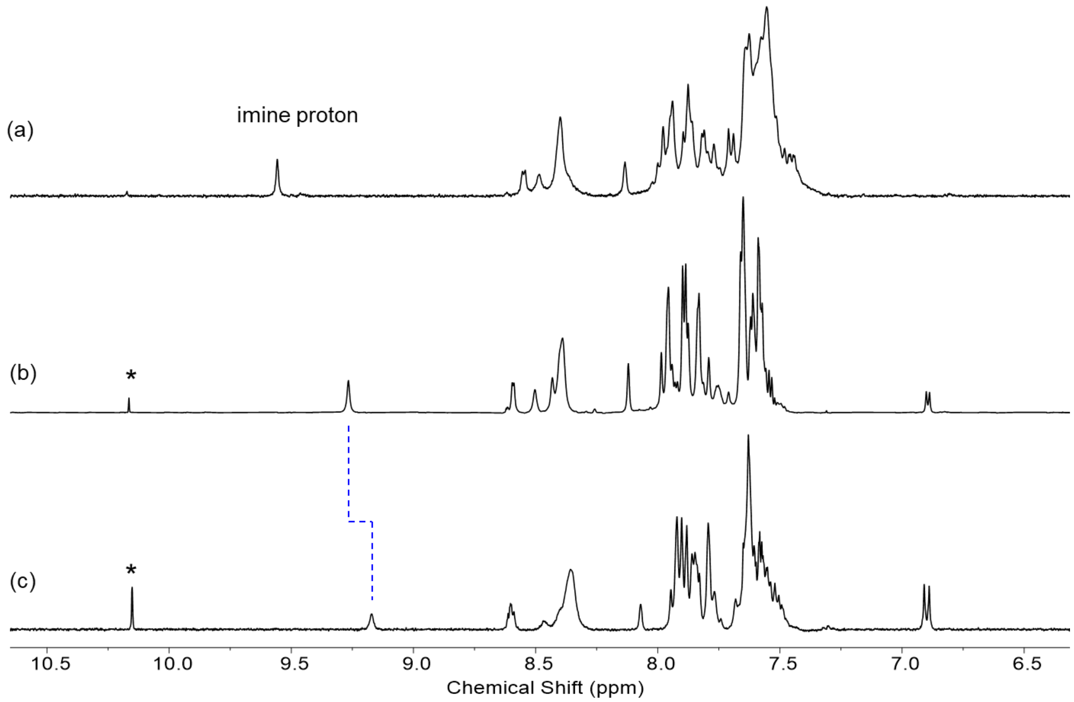

Figure S41. The comparison of the aromatic regions of ^1H NMR of gold assemblies formed in (a) one-pot self-assembly of all building blocks and by the anion-cation pathway using (b) one equivalent of $TBABF_4$ and (c) five equivalents of $TBABF_4$ (500 MHz, CD_2Cl_2, rt). Apart from modest changes in chemical shifts, for example, the change of chemical shift observed for imine protons, the signature of both NMR spectra (b and c) was similar indicating the formation of the same gold assembly but different from the assembly (a) obtained by the one pot pathway. ∗ = **F-PyCHO**

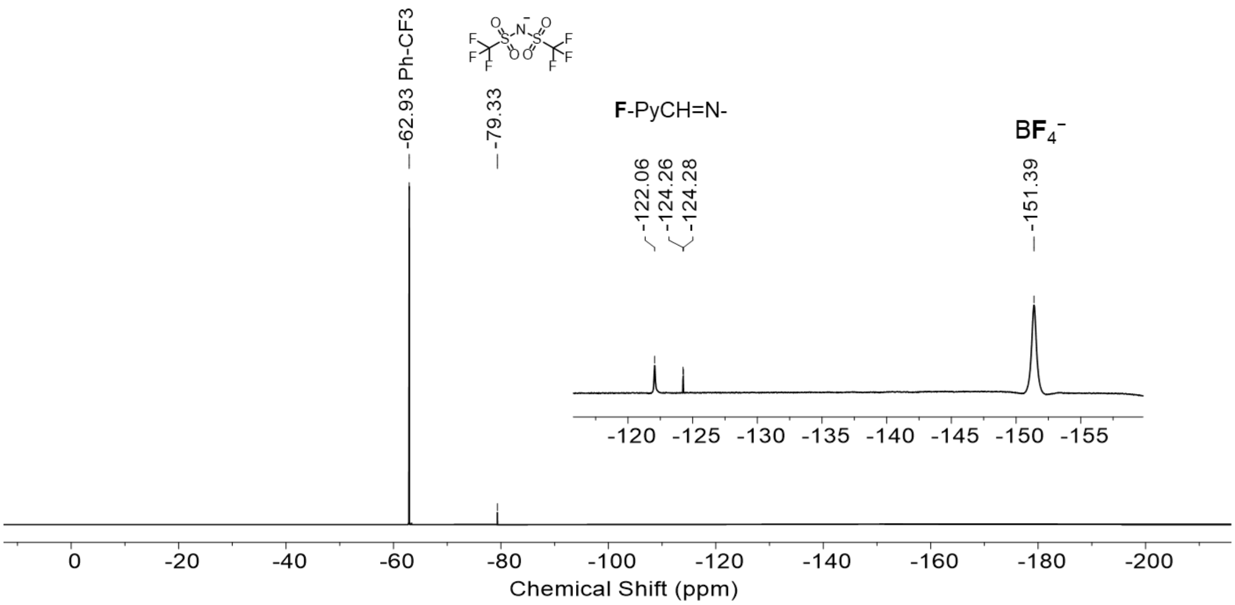

Figure S42. ^{19}F NMR (376 MHz, CD_2Cl_2, rt) of the gold assembly formed by anion-cation pathway formed by anion-cation pathway in presence of excess $TBABF_4$.

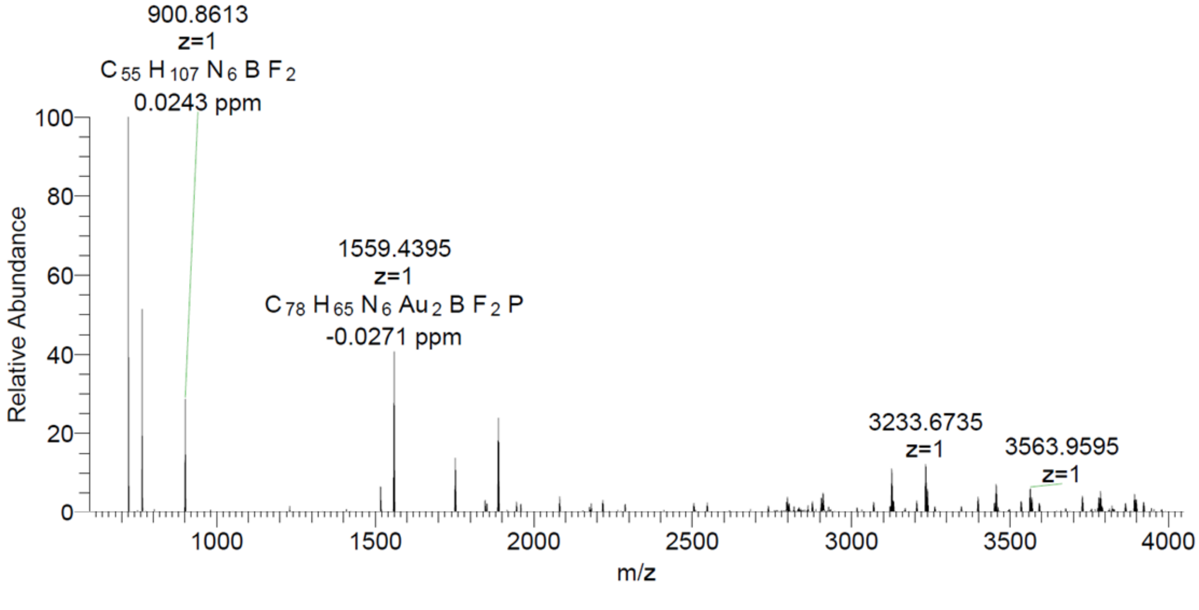

Figure S43. ESI-MS spectrum of the solution at the end of the anion-cation pathway formed using excess BF_4^- (five equivalents of $TBABF_4$) and [**PPh_3**Au]·NTf_2.

15. Self-assembly of Gold(I) Model Complex

Para-toluidine (13.1 mg, 0.122 mmol, 1 equivalent), **F-PyCHO** (15.3 mg, 0.122 mmol, 1 equivalent) and [**PPh**$_3$Au]·NTf$_2$ (90.3 mg, 0.122 mmol, 1 equivalent) were dissolved in 12 mL dichloromethane in a 50 mL round-bottom flask and stirred overnight for 16 h at room temperature. Afterwards, all solvents were evaporated. The resulting yellow precipitate was dissolved in a minimum amount of dichloromethane, 20 mL Et_2O was added in the solution, and kept in a freezer (−32 °C) until yellow single crystals of the model complex was obtained. The yellow crystals were separated and washed with Et_2O (3×5 mL) to obtain the pure model complex (114 mg, 0.120 mmol, 98 %). Some of the crystals were used for the structural determination of the complex in the solid-state by single crystal X-ray diffraction studies.
^{1}H NMR (500 MHz, CD_2Cl_2, rt): δ 9.41 (s, 1H), 8.43-8.36 (m, 1H), 7.96-7.86 (m, 2H), 7.67-7.49 (m, 15H), 7.49-7.43 (m, 2H), 7.28-7.22 (m, 2H), 2.42 (s, 3H). ^{19}F NMR (376 MHz, CD_2Cl_2, rt): −79.43, −121.87. HRMS (ESI) *m/z*: [**PPh**$_3$Au]$^+$ Calcd for $C_{31}H_{26}N_2AuFP^+$ 673.1478; Found 673.1486.

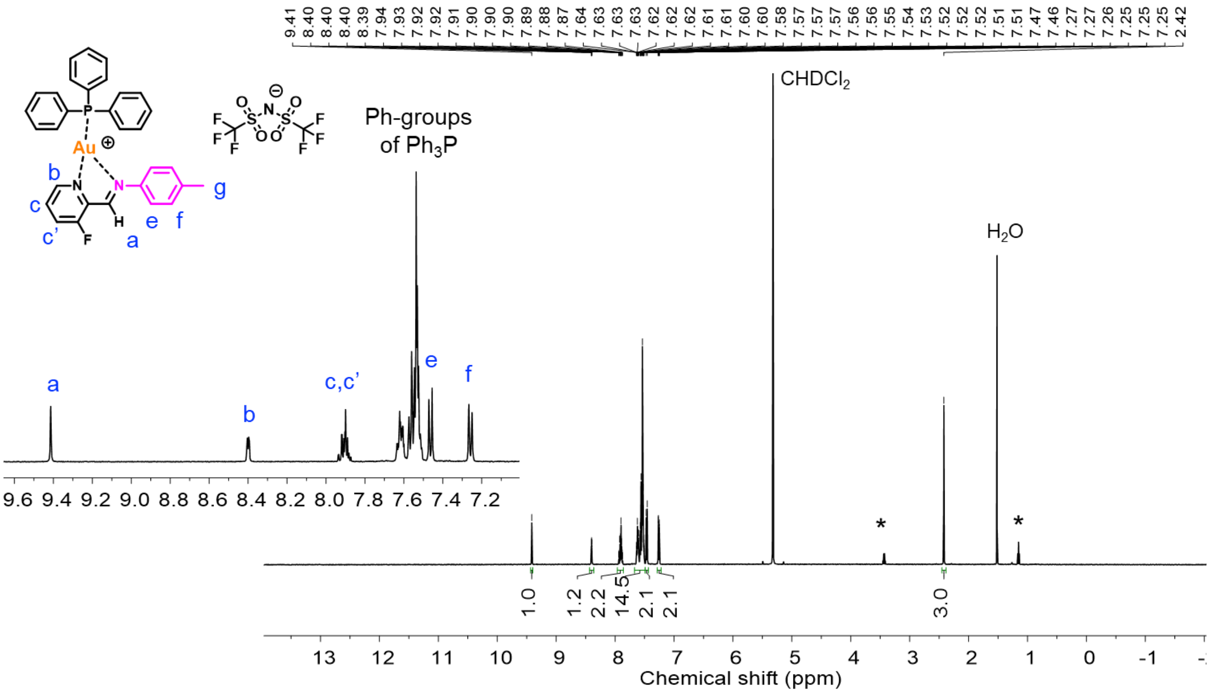

Figure S44. ^{1}H NMR of gold(I) model complex (500 MHz, CD_2Cl_2, rt). * = Et_2O

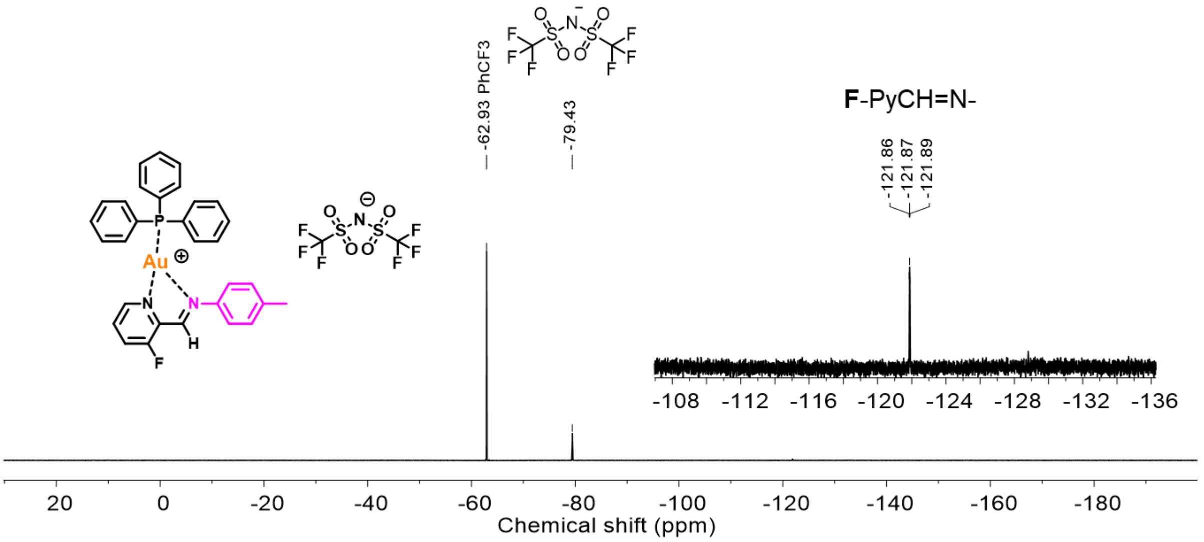

Figure S45. ^{19}F NMR of gold(I) model complex (376 MHz, CD_2Cl_2, rt).

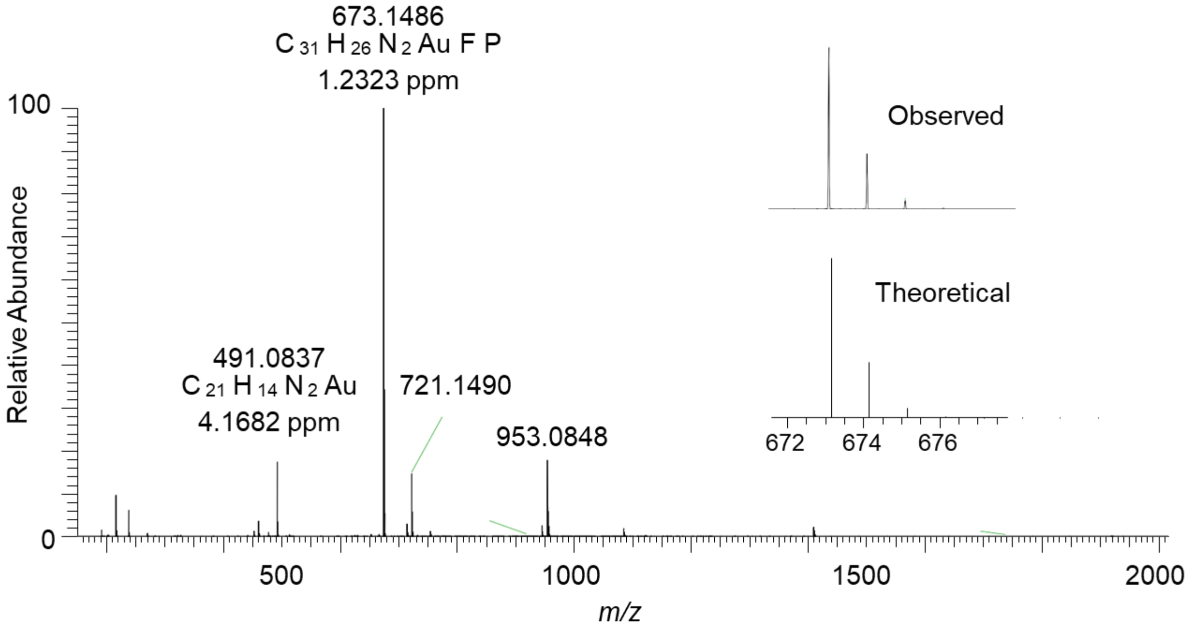

Figure S46. ESI-MS spectrum of gold(I) model complex.

16. Synthesis of [**POP**Cu]·BAr$^F_4$

The chelating phosphine ligand **POP** (7.4 mg, 0.014 mmol, 1 equivalent) and $(CH_3CN)_4CuBAr^F_4$ (15.0 mg, 0.014 mmol, 1 equivalent)[11] were dissolved in 1 mL dichloromethane in a 20 mL dram glass vial. Afterwards, all the solvents were evaporated by blowing argon over the solution and dried under vacuum to obtain [**POP**Cu]·BAr$^F_4$ as a colorless oil (19 mg, 0.013 mmol). ^1H NMR (500 MHz, CD_2Cl_2, rt): δ 7.71 (br s, 8H), 7.55 (s, 4H), 7.50-7.25 (m, 20H), 7.08-6.90 (m, 6H), 6.82-6.76 (m, 2H), 1.53 (s, 6H, overlaps with H_2O). HRMS (ESI) *m/z*: [**POP**Cu]$^+$ Calcd for

$C_{36}H_{28}OCuP_2^+$ 601.0906; Found 601.0899

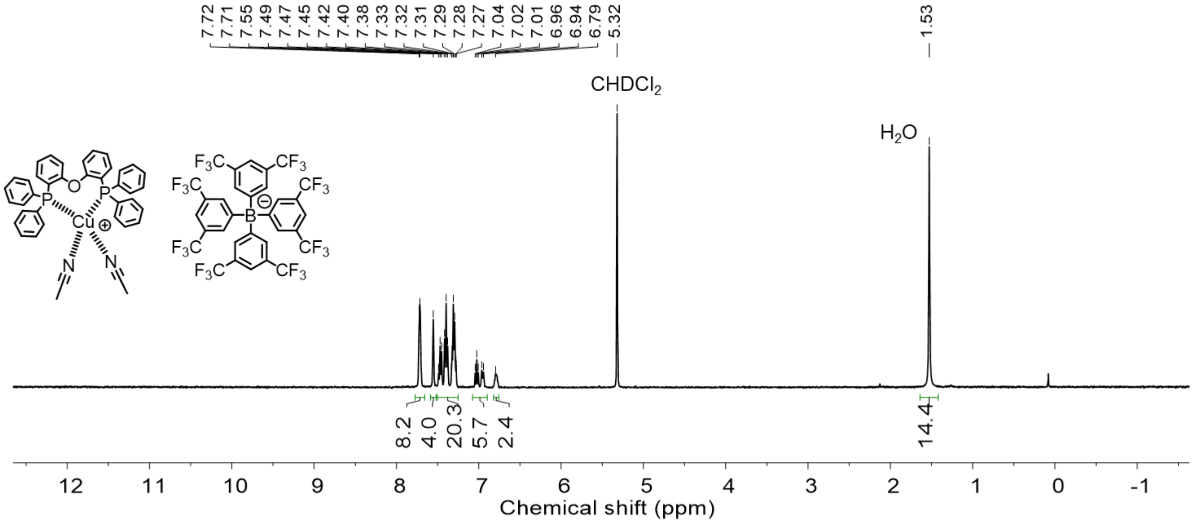

Figure S47. ^{1}H NMR of [**POP**-Cu]·BAr$^F{}_4$ (500 MHz, CD_2Cl_2, rt).

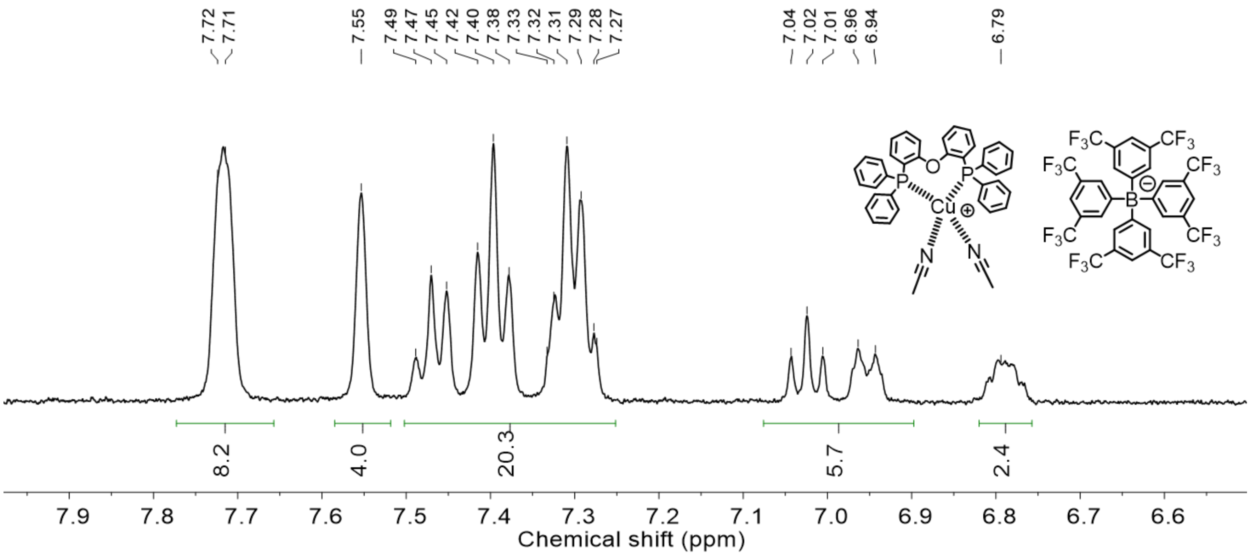

Figure S48. The aromatic region of ^{1}H NMR (Figure S47) of ^{1}H NMR of [**POP-**Cu]·BAr$^F{}_4$ (500 MHz, CD_2Cl_2, rt).

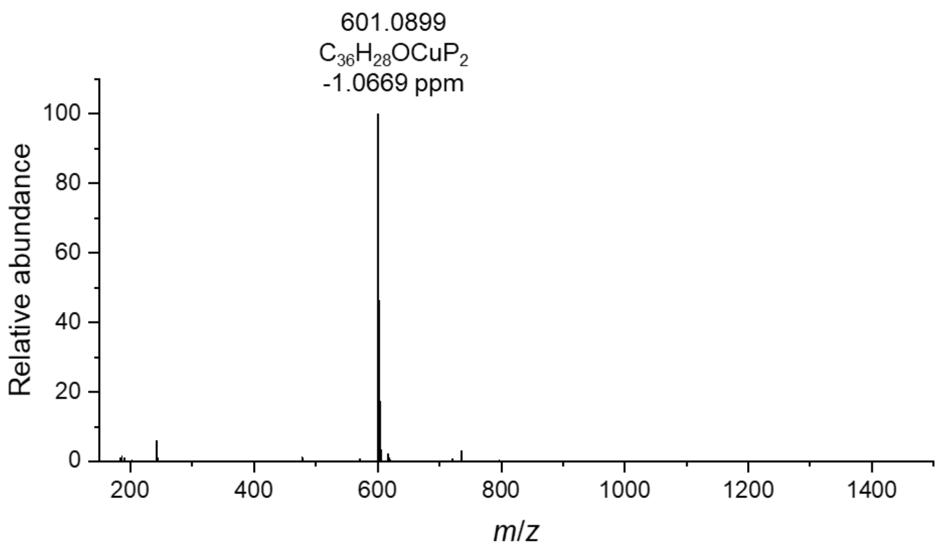

Figure S49. ESI-MS (+ve mode) spectrum of [**POP**-Cu]·BAr^F.

17. NOESY and ROESY Characterization

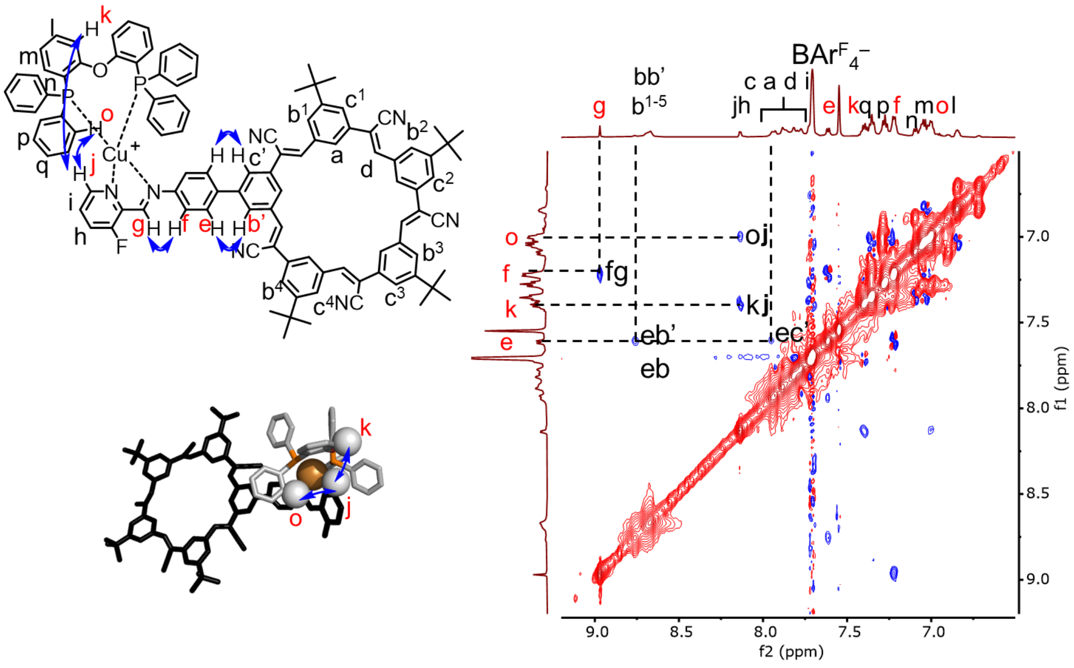

Figure S50. ^{1}H-^{1}H ROESY NMR of [**POP**Cu-**CS**]·BAr$^F{}_4$

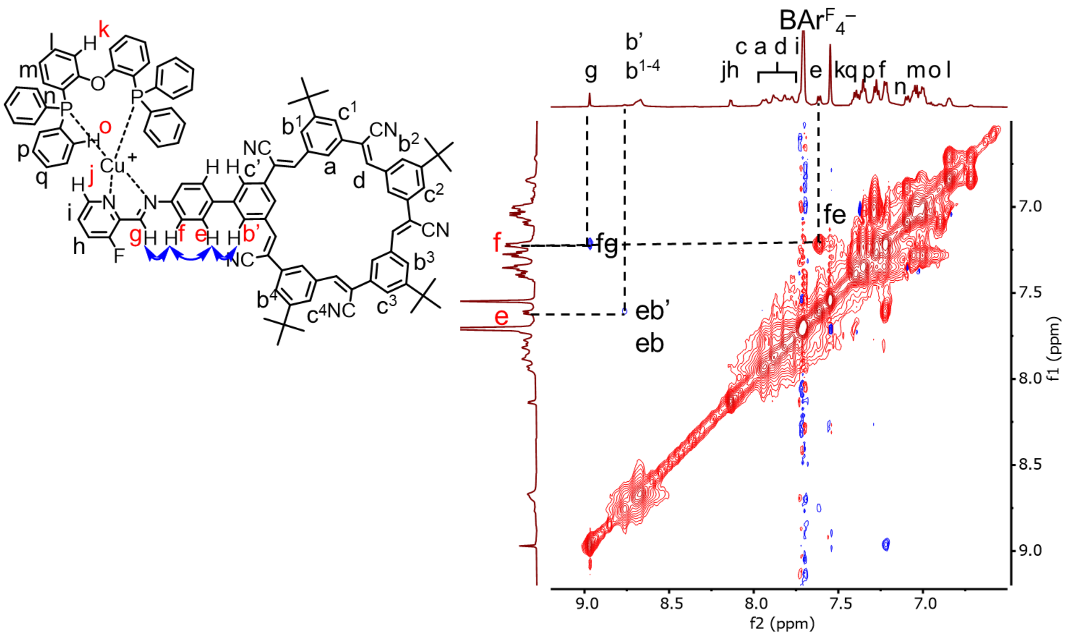

Figure S51. ^{1}H-^{1}H NOESY NMR of [**POP**Cu-**CS**]·BAr$^F{}_4$

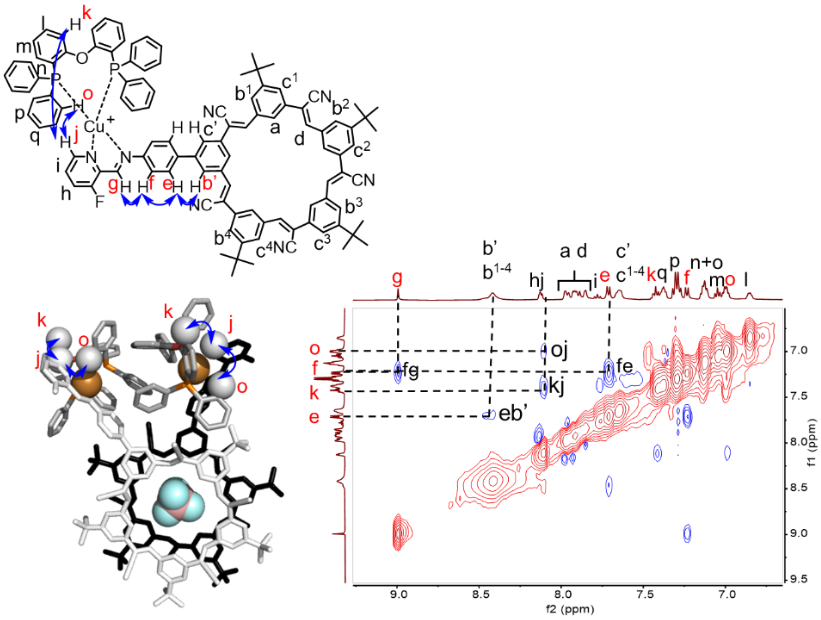

Figure S52. ^{1}H-^{1}H ROESY NMR of [**POP**Cu-**CS**-BF$_4$-**CS**-Cu**POP**]·BF$_4$

Only intramolecular cross-peaks are observed in the dimer (Figure S52). The chemical shifts of ^1H_e and ^1H_c are more similar in dimer (Figure S52) than they are in the monomer complex (Figure S50, S51), so no cross peaks are observed in dimer complex.

18. Diagnostic ^{1}H NMR Shifts upon Self-assembly

Imine formation and metal coordination show characteristic changes in the chemical shifts of key protons upon formation of the target assembly, [**POP**Cu-**CS**-BF_4-**CS**-Cu**POP**]$^+$ (Figure S53). The aldehyde proton at 10.16 ppm (Figure S53c) is consumed to form the imine with its proton (H_g) at the diagnostic position of 9.01 ppm (Figure S53a).[12] Other proton resonances close to the metal binding site are characteristic of imine bond formation and copper complexation.[13] For example, the aniline's ring hydrogen (H_f, 6.84 ppm, Figure S53b) shifts downfield (7.23 ppm, Figure S53a) upon conversion to the imino phenylene, and the phosphine protons (H_k) shift modestly from 7.02 to 7.09 ppm (Figures S53a,d). The cluster of peaks at 8.76 ppm in **NH$_2$CS** are assigned to the outer H_b protons of the macrocycle. After one-pot assembly, these shift upfield to 8.41 ppm matching the parent cyanostar.[14]

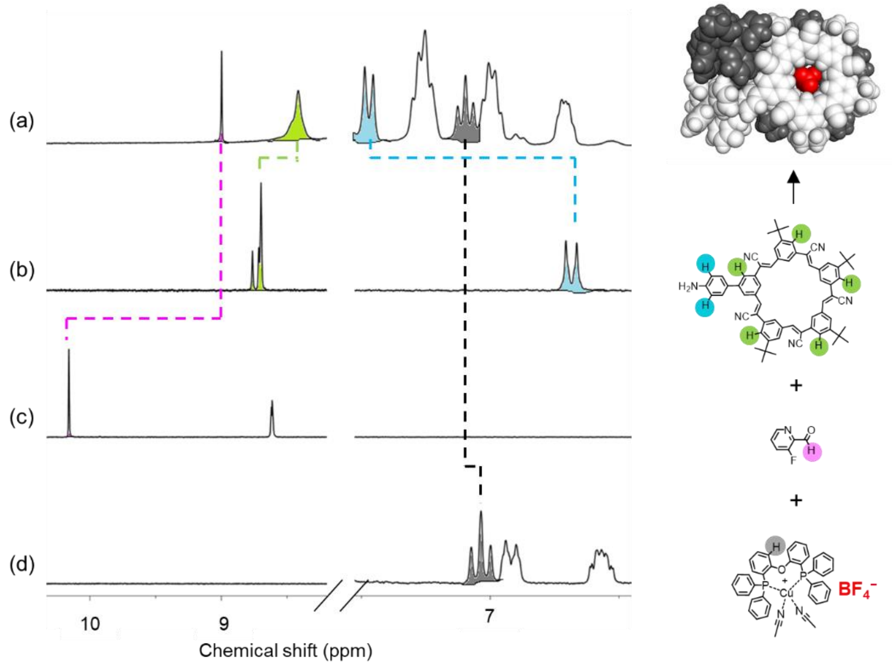

Figure S53. Diagnostic ^{1}H NMR peaks (CD_2Cl_2, 2 mM) of (a) [**POP**Cu-**CS**-BF_4-**CS**-Cu**POP**]$^+$ relative to its components: (b) **NH$_2$-CS**, (c) **F-PyCHO** and (d) [**POP**Cu$(MeCN)_2$]BF_4.

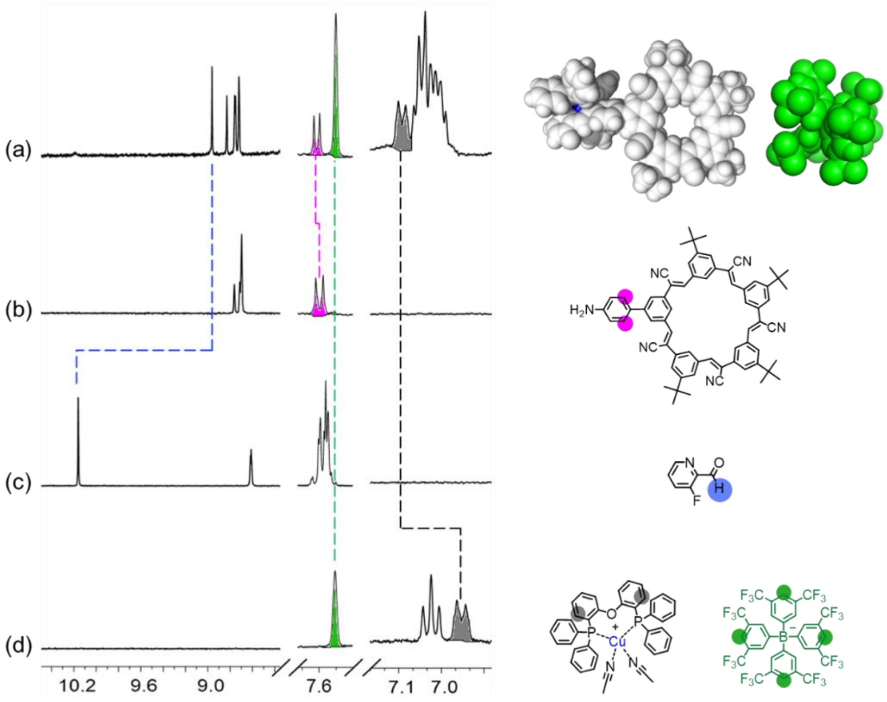

Figure S54. Diagnostic ^{1}H NMR peaks (CD_2Cl_2, 3 mM) of (b) [**POP**Cu-**CS**]$^+$ and its components (c) **NH$_2$-CS**, (d) **F-PyCHO** and (e) [**POP**Cu(MeCN)$_2$]BAr$^F{}_4$.

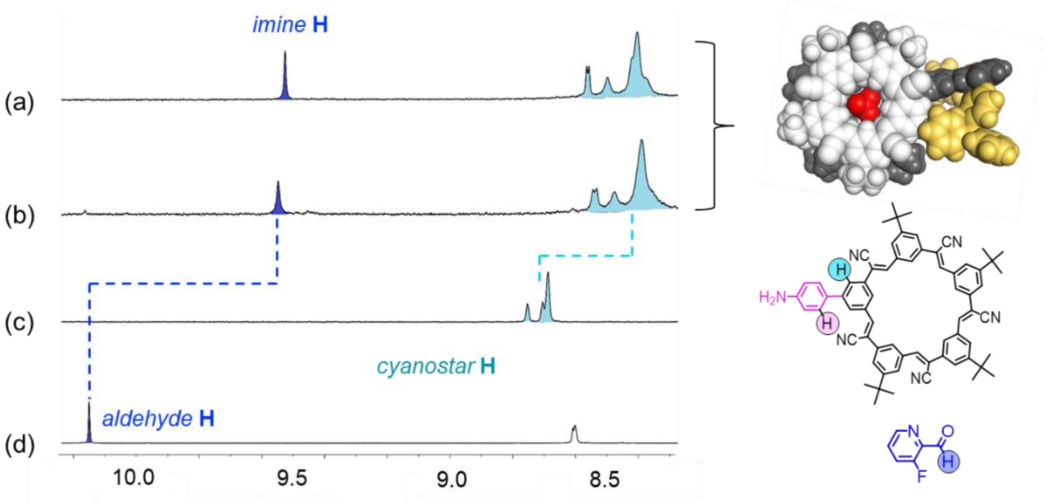

Figure S55. Diagnostic ^{1}H NMR peaks (CD_2Cl_2, 0.5 mM) for [**PPh$_3$**Au-**CS**-BF_4-**CS**-Au**PPh$_3$**]$^+$ obtained by (a) cation-anion pathway, and (b) one-pot assembly as well as key components (c) **NH$_2$-CS**, and (d) **F-PyCHO**.

19. ^{1}H NMR Spectra of Gold(I) Assemblies Obtained by Different Pathways

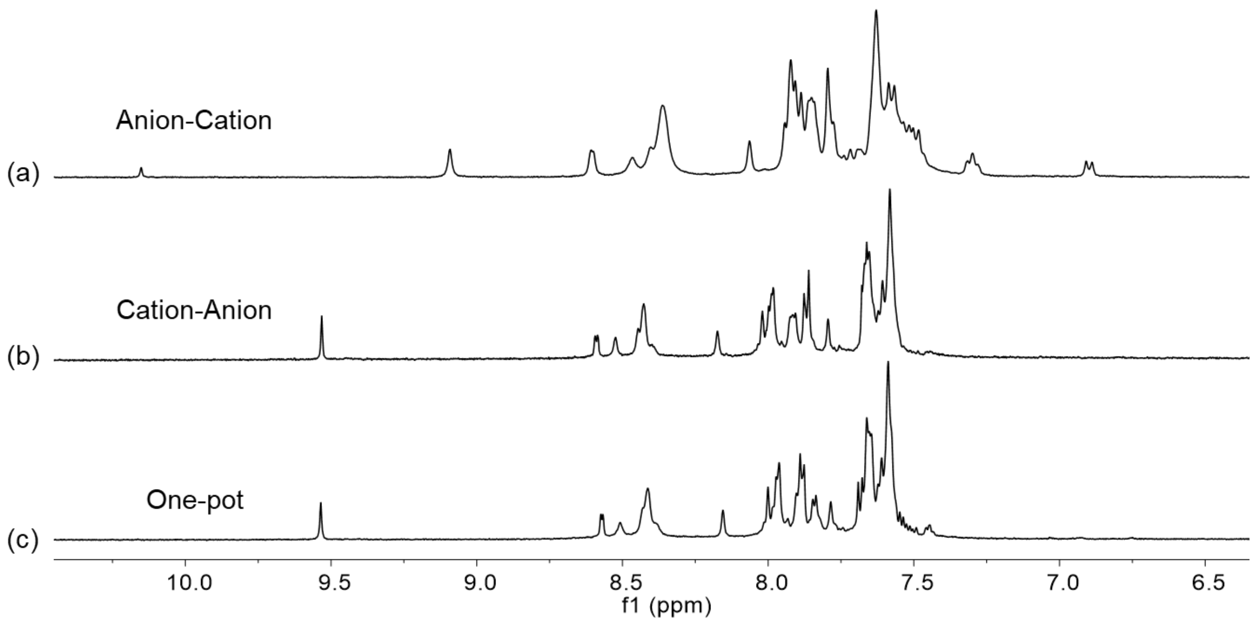

Figure S56. Diagnostic ^{1}H NMR peaks (CD_2Cl_2, 0.5 mM) of gold(I) assemblies obtained by (a) anion-cation, (b) cation-anion, (c) one-pot self-assembly.

20. Titration of [**POP**Cu-**CS**]·BAr^F_4 with $TBAClO_4$

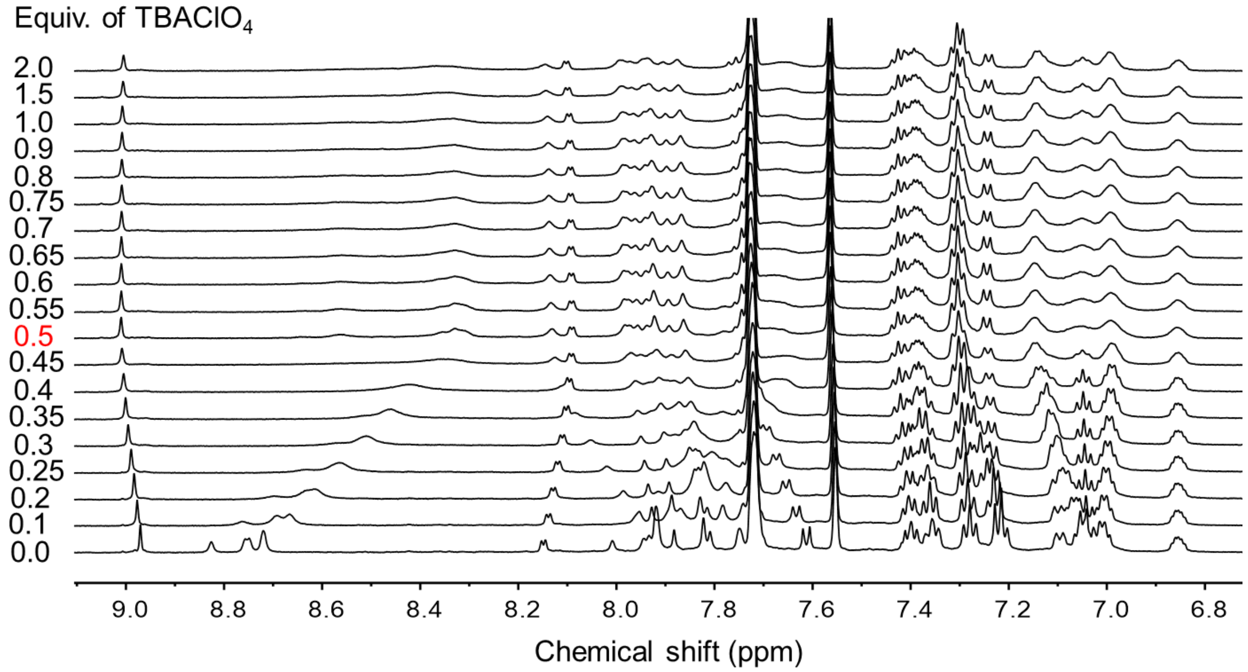

Figure S57. ^{1}H NMR titrations of [**POP**Cu-**CS**]·BAr^F_4 (2 mM, 400 MHz, rt) in CD_2Cl_2 with $TBAClO_4$.

21. ^{1}H NMR Titration Controls with Cyanostar

Parent cyanostar forms a 2:1 [**pCS**-BF_4-**pCS**]$^{-}$ assembly.[14] A ^{1}H NMR titration monitoring the addition of $TBABF_4$ to cyanostar (Figure S50a, CD_2Cl_2) shows fast exchange peaks. Based on the H_b on cyanostar, we see conversion at 0.5 equivalents (Figure 50b). The inner cavity protons, H_a and H_d, shift downfield, and the outer cavity protons, H_b and H_c shift upfield (Figure 50a), which is consistent with BF_4^- binding inside of the cyanostar cavity to form a 2:1 complex.

Ion pairing is known to play a role in CD_2Cl_2, and the inflection points of the H_α on TBA are consistent with this expectation (Figure S50c). Between 0 and 0.5 equivalents, H_α on TBA^+ shifts upfield, which is consistent with the cyanostar binding BF_4^- and disrupting ion pairing with the free TBA^+. Upon the addition of excess $TBABF_4$, H_α on TBA shifts downfield, which is consistent with the formation of ion pairs between TBA^+ and excess BF_4^-. TBA^+ likely also form ion pairs with the 2:1 assembly which is shown by a slight downfield shift of H_b at higher concentrations of $TBABF_4$ (Figure S50a-b).

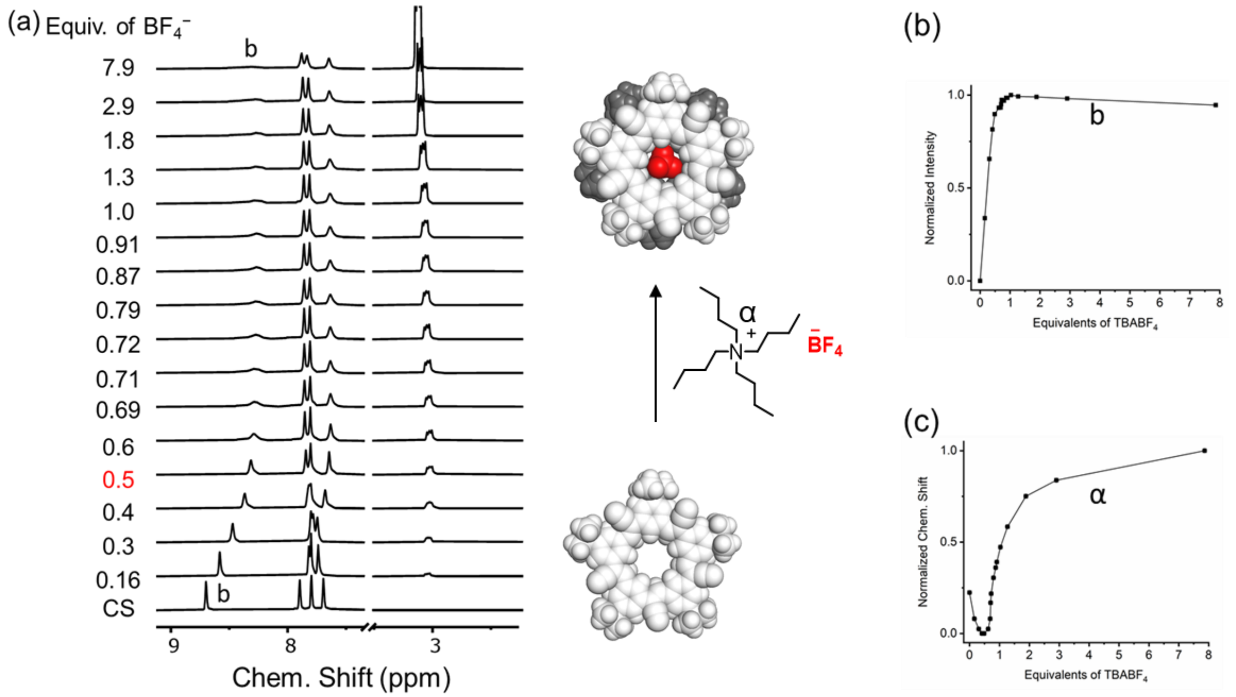

Figure S58. (a) ^{1}H NMR titration of $TBABF_4$ into **pCS** (4 mM) in CD_2Cl_2 (298 K, 400 MHz). Normalized peak intensity of (b) cyanostar H_b and (c) TBA^+ H_α during titration.

To verify that BF_4^- binds inside the cyanostar cavity, a ^{19}F NMR titration monitoring the addition of $TBABF_4$ to **pCS** was examined. The ^{19}F NMR titration shows medium exchange peaks (Figure S50a), which differs from the fast exchange ^{1}H NMR peaks (Figure S50a). Early in the titration, a single ^{19}F peak is observed at –148 ppm (Figure S51a), which shows an inflection point at 0.5 equivalents $TBABF_4$ (Figure S51b). This peak is shifted 3 ppm downfield from uncomplexed

TBABF$_4$, which is consistent with the binding of BF_4^- inside cyanostar. The inflection point at 0.5 equivalents is consistent with the formation of a 2:1 complex with **pCS**. Upon further addition of TBABF$_4$, an additional peak appears at 152 ppm (Figure S51a) that grows in intensity (Figure S51c). This peak is similar to uncomplexed TBABF$_4$ at 151 ppm, which is consistent with excess TBABF$_4$ present is solution.

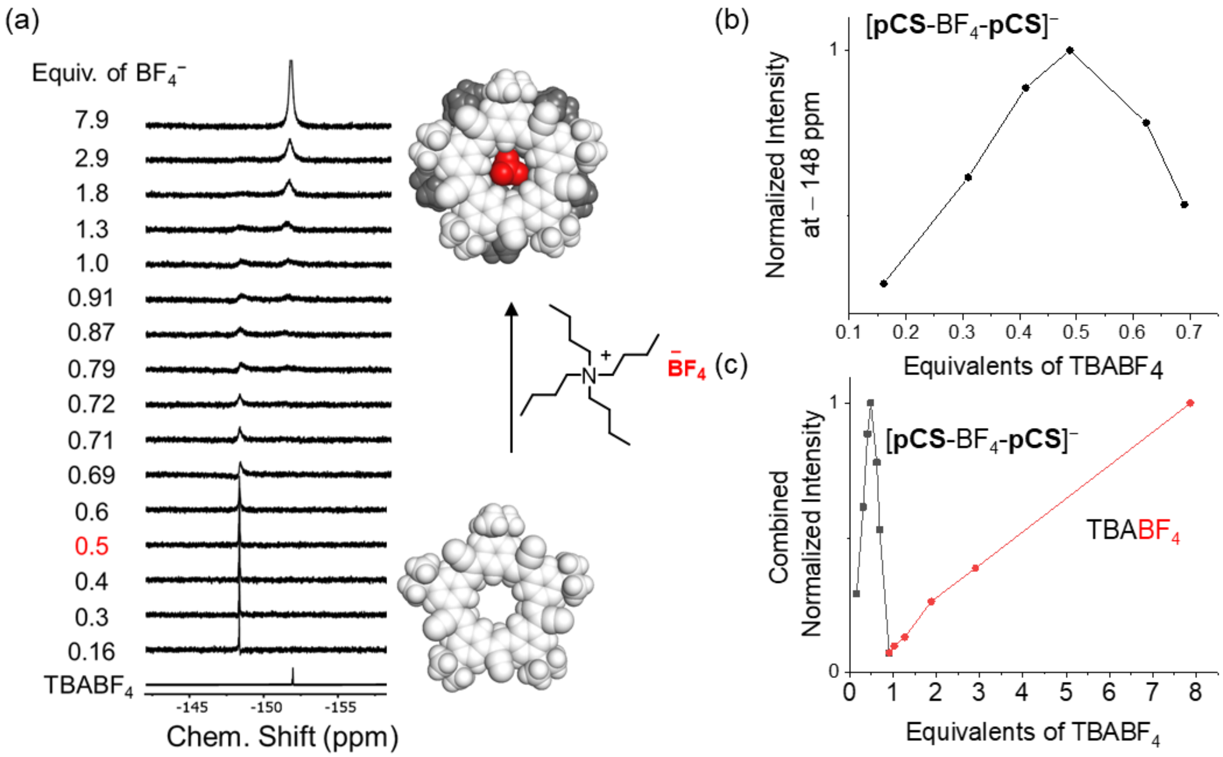

Figure S59. (a) ^{19}F NMR titration of TBABF$_4$ into **pCS** (4 mM) in CD_2Cl_2 (298 K, 400 MHz). Normalized peak intensity of (b) [**pCS**-BF$_4$-**pCS**]$^-$ and at –148 ppm only, (c) [**pCS**-BF$_4$-**pCS**]$^-$ (–148 ppm) combined with TBABF$_4$ (–152 ppm).

22. Diffusion NMR: Pulse Gradient Spin Echo (PGSE) experiments

Table S1. Diffusion coefficients (D) for all peak resonances of [**POP**Cu-**CS**-BF$_4$-**CS**-Cu**POP**]$^+$ made by one-pot.

ppm	D (m^2 / s) ×10^{-10}
9	4.83
8.41	4.46
8.12	4.45
7.96	4.18

7.9	4.65
7.86	4.83
7.81	4.95
7.77	4.46
7.71	4.70
7.64	4.92
7.42	4.41
7.38	5.30
7.34	5.32
7.3	5.05
7.24	5.26
7.13	4.74
7.05	4.79
7	4.99
6.86	5.23

Mean: $4.8 \pm 0.6 \times 10^{-10}$ m^2 / s

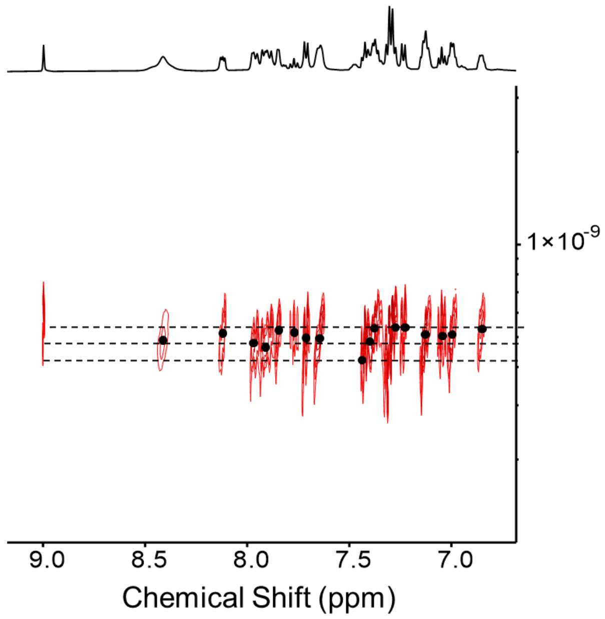

Figure S60. DOSY for [**POP**Cu-**CS**-BF_4-**CS**-Cu**POP**]$^+$ made by one-pot.

Table S2. Diffusion coefficients (*D*) for all peak resonances of [**POP**Cu-**CS**]$^+$

ppm	*D* (m^2 / s) × 10^{-10}
8.96	5.41
8.83	5.47
8.76	5.38
8.72	5.39
8.14	5.39
8.02	5.56
7.95	5.26
7.92	5.39
7.88	5.56
7.82	5.48
7.74	5.48
7.61	5.61

7.4	5.49
7.35	5.39
7.27	5.53
7.21	5.40
7.1	5.32
7.07-6.98	5.40
6.85	5.55
1.5	5.54

Mean: $5.5 \pm 0.2 \times 10^{-10}$ m^2 / s

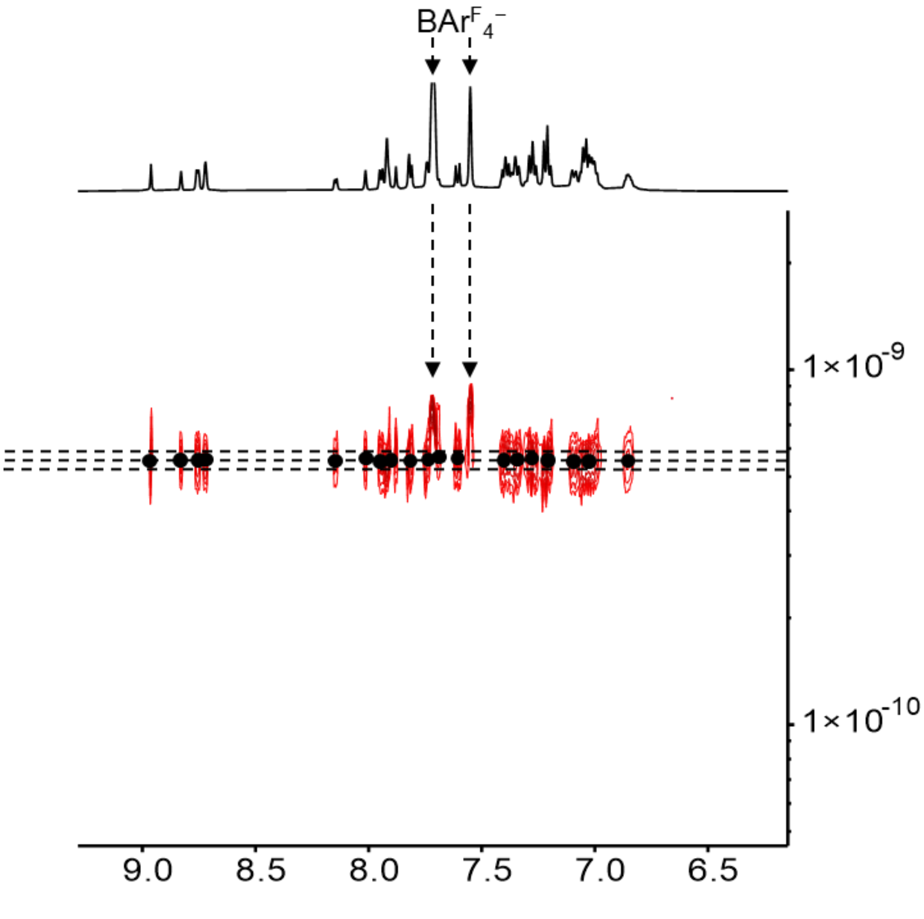

Figure S61. DOSY for [**POP**Cu-**CS**]$^+$

Table S3. Diffusion coefficients (*D*) for all peak resonances of [**POP**Cu-**CS**-BF_4-**CS**-Cu**POP**]$^+$ made by cation-anion pathway

ppm	D (m^2 / s) × 10^{-10}
9.02	4.9
8.39	4.9
8.1	4.8
7.93	4.7
7.88	4.7
7.85	4.9
7.82	4.9
7.65	4.4
7.4	4.5
7.3	4.5
7.24	4.7
7.14	4.5
7.04	4.4
6.99	4.5
6.86	4.3

Mean: $4.6 \pm 0.4 \times 10^{-10}$ m^2 / s

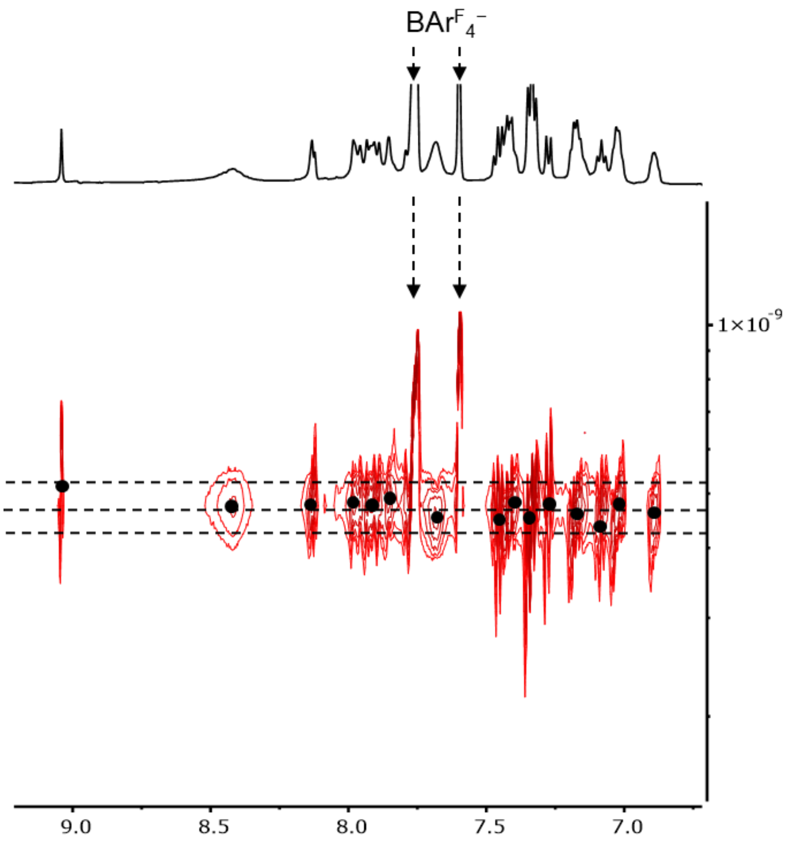

Figure S62. DOSY for [**POP**Cu-**CS**-BF_4-**CS**-Cu**POP**]$^+$ made by cation-anion pathway

Table S4. Diffusion coefficients (*D*) for all peak resonances of [**POP**Cu-**CS**-ClO_4-**CS**-Cu**POP**]$^+$ made by cation-anion pathway

ppm	D (m^2 / s) × 10^{-10}
9	4.47
8.35	4.73
8.14	4.41
8.1	4.51
7.97	4.34
7.93	4.43
7.9	4.51
7.87	4.72

7.75	4.71
7.64	4.96
7.39	4.57
7.29	4.50
7.24	4.76
7.13	4.61
7.04	4.59
6.99	4.50
6.84	4.56

Mean: $4.6 \pm 0.2 \times 10^{-10}$ m^2 / s

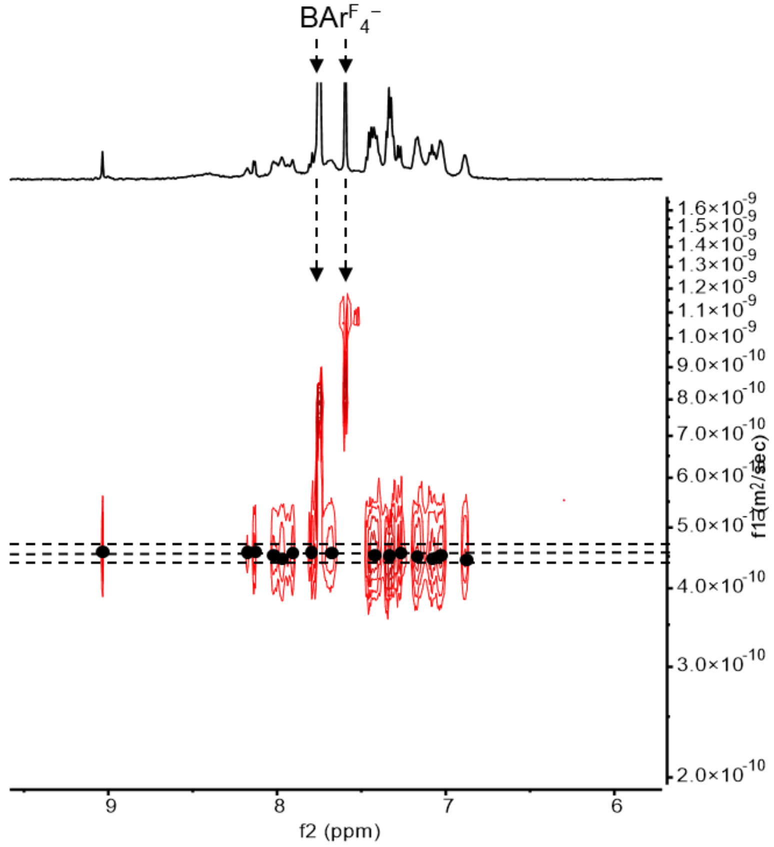

Figure S63. DOSY for [**POP**Cu-**CS**-ClO_4-**CS**-Cu**POP**]$^+$ made by cation-anion pathway

Table S5. Diffusion coefficients (*D*) for all peak resonances corresponding to [**CS**-BF_4-**CS**]$^{-}$

ppm	*D* (m^2 / s) × 10^{-10}
8.95	5.5
8.65	5.5
8.46	5.1
8.36	5.4
8.05	5.4
7.92	5.3
7.85	5.5
7.8	5.6
7.7	5.1
7.64	5.4
7.56	6.0
7.48	6.0
6.92	5.7
1.61	5.7

***Not a monodisperse species, so diffusion coefficient of imine peak at 8.95 ppm was used to determine diffusion coefficient for [**CS**-BF_4-**CS**]$^{-}$.

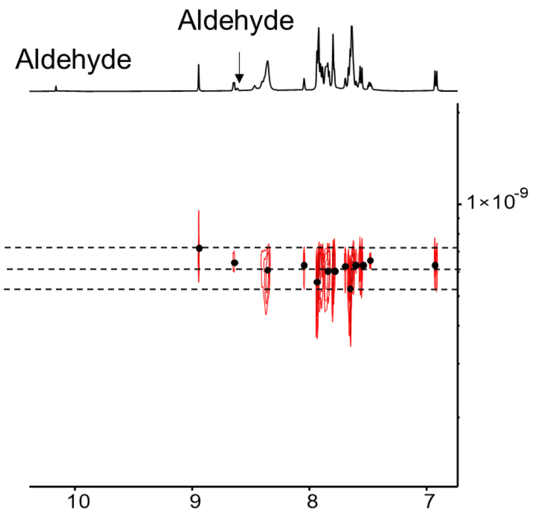

Figure S64. DOSY for [**CS**-BF_4-**CS**]$^-$ with unreacted amine and reacted imines.

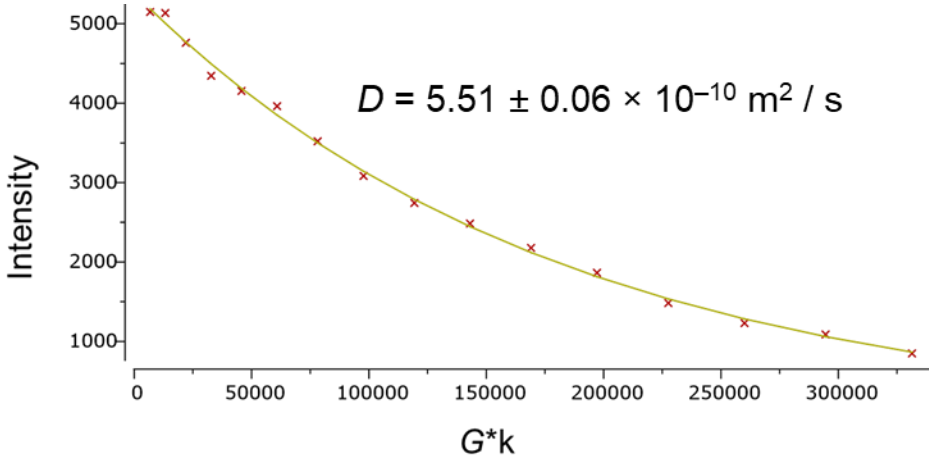

Figure S65. Fitting of pulse field gradient to determine diffusion coefficient of imine resonances of [**CS**-BF_4-**CS**]$^-$

Table S6. Diffusion coefficients (*D*) for [**POP**Cu-**CS**-BF_4-**CS**-Cu**POP**]$^+$ made by anion-cation pathway

ppm	D (m^2 / s) × 10^{-10}
9.01	4.7
8.1	4.8
7.88	4.9
7.76	4.9

7.64	4.9
7.42	4.8
7.38	4.9
7.3	4.8
7.24	5.1
7.13	4.8
7.05	4.8
6.99	4.8
6.86	4.8
1.56	5.0

Mean: $4.9 \pm 0.2 \times 10^{-10}$ m^2 / s

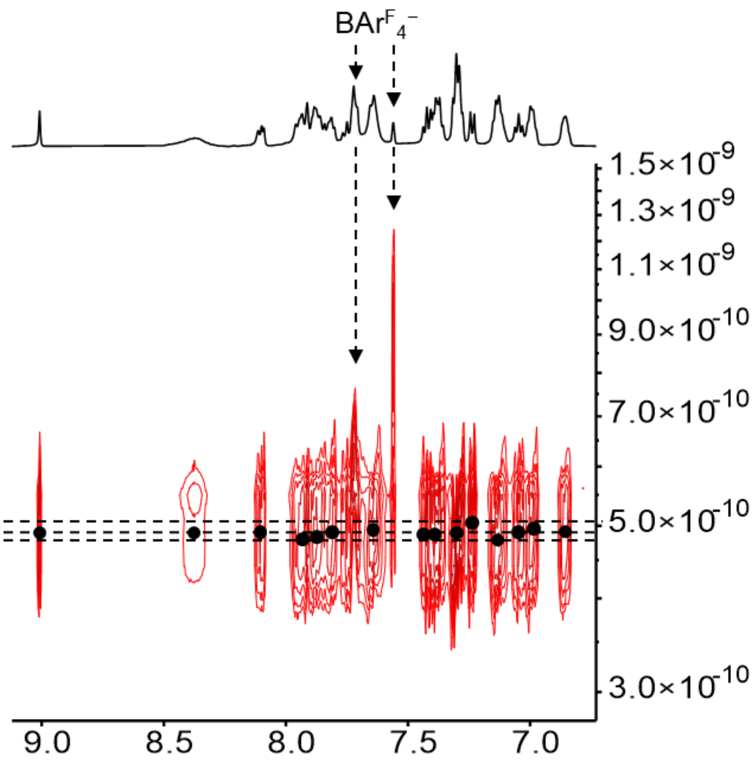

Figure S66. DOSY for [**POP**Cu-**CS**-BF_4-**CS**-Cu**POP**]$^+$ made by anion-cation pathway

Table S7. Diffusion coefficients (*D*) for [**PPh**$_3$Au-**CS**-BF$_4$-**CS**-Au**PPh**$_3$]$^+$ made by one-pot

ppm	D (m^2 / s) × 10^{-10}
9.56	4.5
8.61	4.1
8.55	4.0
8.54	3.8
8.41	4.5
8.14	4.1
7.98	3.7
7.95	4.5
7.88	4.7
7.79	4.7
7.7	4.3
7.63	4.9
7.57	6.0
7.51	6.3
7.45	6.3
1.55	5.0

Mean: $5 \pm 2 \times 10^{-10}$ m^2 / s

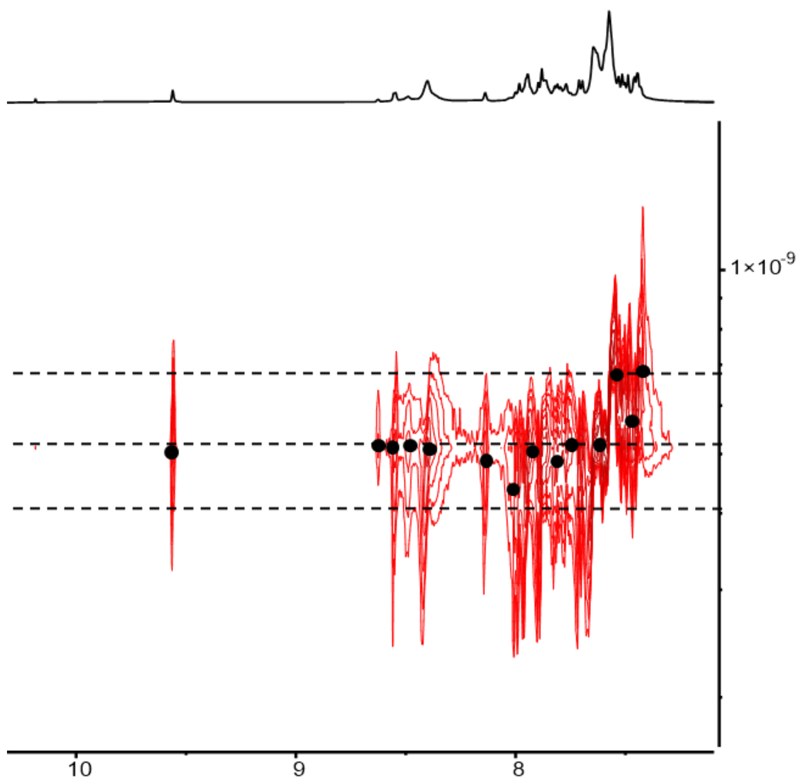

Figure S67. DOSY for [**PPh$_3$**Au-**CS**-BF$_4$-**CS**-Au**PPh$_3$**]$^+$ made by one-pot

Table S8. Diffusion coefficients (*D*) for [**PPh$_3$**Au-**CS**]$^+$

ppm	D (m^2 / s) × 10^{-10}
9.53	4.9
8.63	5.0
8.54	4.9
8.53	5.1
8.02	5.0
8	5.1
7.96	5.2

7.92	5.2
7.87	5.0
7.82	5.1
7.76	5.1
7.7	5.1
7.65	5.5
7.58	5.6

Mean: 5.1 ± 0.2 × 10^{-10} m^2 / s

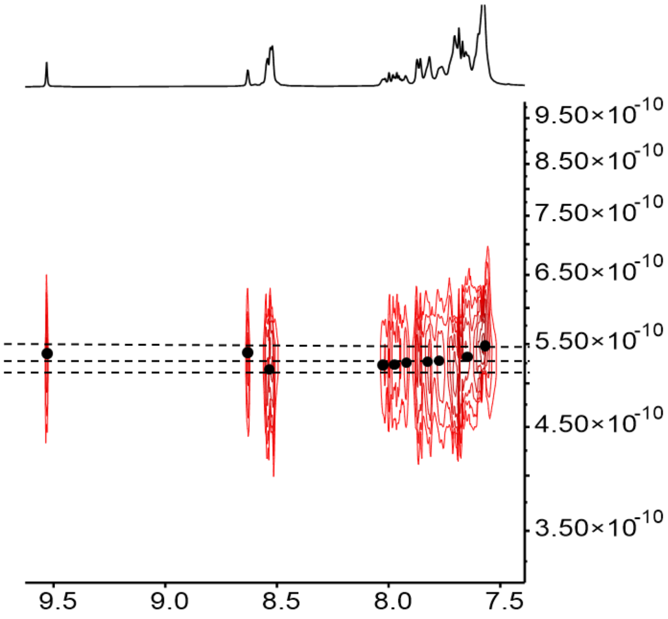

Figure S68. DOSY for [**PPh**$_3$Au-**CS**]$^+$

Table S9. Diffusion coefficients (*D*) for [**PPh**$_3$Au-**CS**-BF_4-**CS**-Au**PPh**$_3$]$^+$ made by cation-anion pathway

ppm	D (m^2 / s) × 10^{-10}
9.55	5.8
8.57	5.0
8.51	5.0

8.41	5.1
8.16	5.5
7.99	5.1
7.93	4.5
7.89	5.0
7.84	5.2
7.79	5.1
7.66	5.0
7.59	5.6

Mean: 5.2 ± 0.6 × 10^{-10} m^2 / s

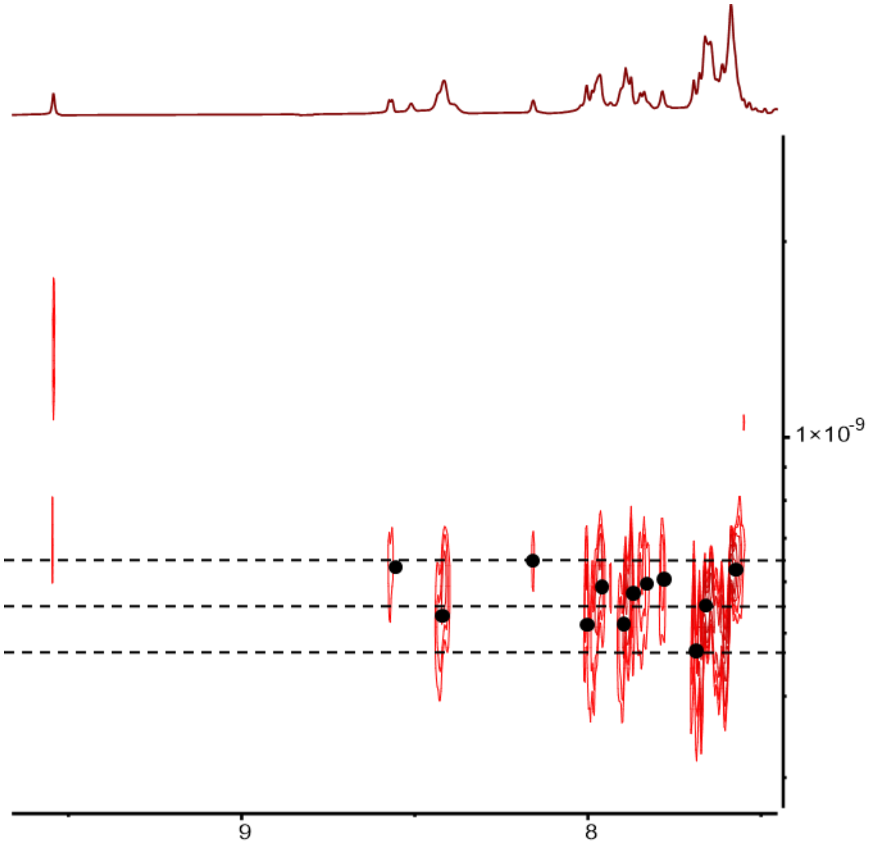

Figure S69. DOSY for [**PPh$_3$**Au-**CS**-BF_4-**CS**-Au**PPh$_3$**]$^+$ made by cation-anion

23. Orientation of the Macrocycles in the Dimer

The diffusion coefficients and ROESY correlations were examined to help distinguish one arrangement over the other (*syn*, *meta* and *anti*). The diffusion coefficients for the monomeric control [**POP**Cu-**CS**]$^+$, 5.5 ±0.2 × 10^{-10} m^2 / s, and the target complex [**POP**Cu-**CS**-BF_4-**CS**-Cu**POP**]$^+$ from the one-pot assembly, 4.8 ±0.6 × 10^{-10} m^2 / s, are the same within error. The diffusion coefficients were the same whether accessed from either cation-anion, 4.6 ±0.4 × 10^{-10} m^2 / s, or anion-cation pathways, 4.9 ±0.2 × 10^{-10} m^2 / s, and they are barely smaller than the monomeric control. As a result, the ratio of [**POP**Cu-**CS**-BF_4-**CS**-Cu**POP**]$^+$ and [**POP**Cu-**CS**]$^+$ ranges 1.0–1.4. The ratios of the three conformations relative to the control and as calculated from molecular modelling are 1.0, 1.2, and 1.4 for the *syn*, *meta* and *anti*, respectively. As a result, diffusion NMR cannot be used to determine the conformation, but the *syn* and *meta* isomers better match the experimental data. ^{1}H-^{1}H ROESY studies on the dimeric [**POP**Cu-**CS**-BF_4-**CS**-Cu**POP**]$^+$ featured intramolecular interactions that were also present in a monomeric control, [**CuCS**]$^+$.

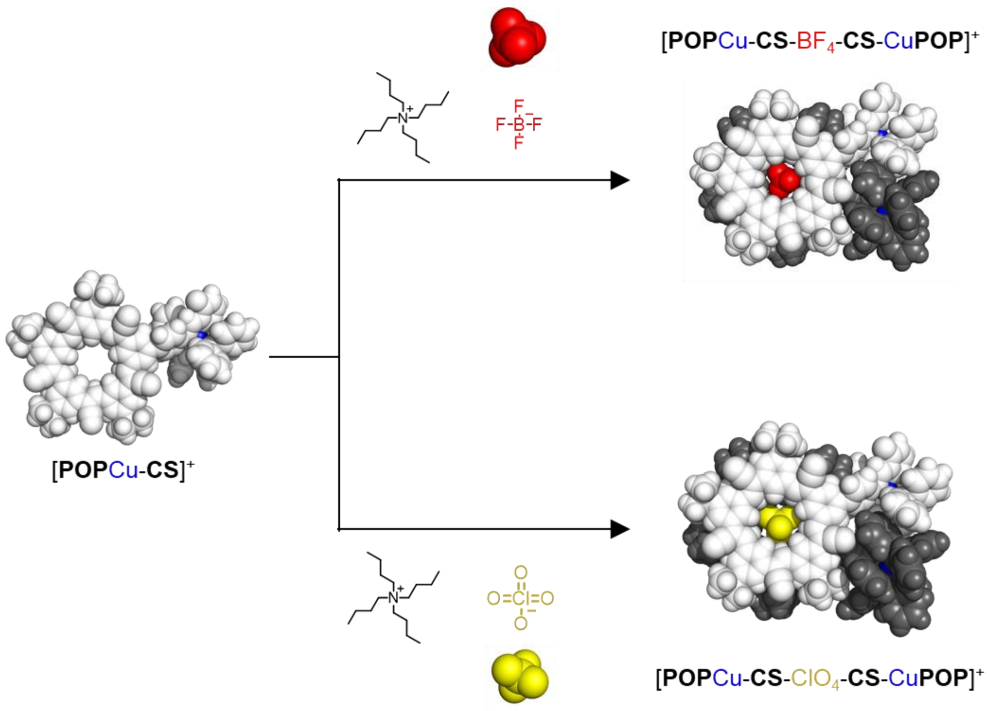

Figure S70. Reaction to form [**POP**Cu-**CS**-BF_4-**CS**-Cu**POP**]$^+$ and [**POP**Cu-**CS**-ClO_4-**CS**-Cu**POP**]$^+$ from [**POP**Cu-CS]$^+$ intermediate.

24. Choice of Anions for the Dimerization of Cationic Intermediates and Follow-Up Studies to Investigate Viability of Sequence-Dependent Target Product

Our selection of anions was strategic. The BF_4^- and ClO_4^- anions bind strongly to cyanostar but are typically weakly coordinating with metal cations. We conducted additional studies using

dibenzyl phosphate (Figure S71-S72, S75) and dibutyl phosphate (Figure S73-S74) that each bind well to cyanostar[15] but can also coordinate to metal complexes. We hypothesized that these stronger anions would interact with the metal ion to disrupt the overall assembly when we used either a one-pot method or prepared the cationic intermediate. We also hypothesized that pre-complexation of these anions with the macrocycle would prevent the phosphates from coordinating with the metal ions. To evaluate this idea, we pre-complexed these phosphate anions with cyanostar. For this purpose, we used control compounds: parent cyanostar (**pCS**), dibutyl phosphate, dibenzyl phosphate, and a model Cu^+ complex. However, we found that pre-complexation failed to prevent the phosphate from coordination of the metal centers. We observed that the copper complexes decomposed in 24 hours (Figure S71-S75).

We investigated the interactions of dibutyl phosphate (**P**$^−$) with Au^+-based cysnostar complexes. We added **P**$^−$ to [**PPh$_3$**Au-**CS**]$^+$ $NTf_2^−$ (Figure S76, the middle spectrum) and expected to form [**PPh$_3$**Au-**CS**-**P**-**CS**-Au**PPh$_3$**]$^+$. The phosphate peak ($H_α$) at 4.5 ppm (Figure S76, inset) suggested that some phosphate anions form [3]pseudorotoxane in accordance with our previous report,[16] while the rest of the phosphate did not bind with cysnostar cavity. The broad signature of NMR peaks, especially in the aromatic region, did not allow us to characterize the spectrum to a well-defined assembly.

To deconvolute the spectrum, we did the following control experiments. We added **P**$^−$ to the model gold(I) complex (Figure S77d). We saw a pronounced upfield shift of the imine proton (H_a), which suggested the detachment of gold ion from the model complex. We inferred that the ionic interaction between positively charged Au^+ and **P**$^−$ was stronger than the coordination of Au^+ with pyridyl-imine ligand. We also observed a small peak (marked as * in Figure S77d) corresponding to the aldehyde proton of **F-PyCHO**. So, the addition of **P**$^−$ to the model gold(I) complex resulted in a mixture of **Ph$_3$P**Au^+**P**$^−$, imine, and its Schiff base precursors. To this mixture, we added **pCS** (2 equiv. with respect to **P**$^−$) (Figure S77c). Our expectation was that if the binding of **P**$^−$ with **pCS** were higher than the ionic interactions between **Ph$_3$P**Au^+ and **P**$^−$, **pCS** would bind with **P**$^−$ to form a [3]pseudorotaxane, and the remaining **Ph$_3$P**Au^+ would reform the model gold(I) complex. The NMR spectrum (Figure S77c) showed that we formed the desired pseudorotaxane based on **pCS** and **P**$^−$, but we did not reform the model gold(I) complex. The broad new peaks (marked as # in Figure S77c) indicated the ionic interaction of **Ph$_3$P**Au^+ with **P**$^−$, which was bound in the pseudorotaxane.

Taking all these control experiments together, we concluded that the addition dibutyl phosphate (**P**$^−$) to [**PPh$_3$**Au-**CS**]$^+$ $NTf_2^−$ led to decomplexation of gold ion, resulting in a soup of different fragments (Figure S78).

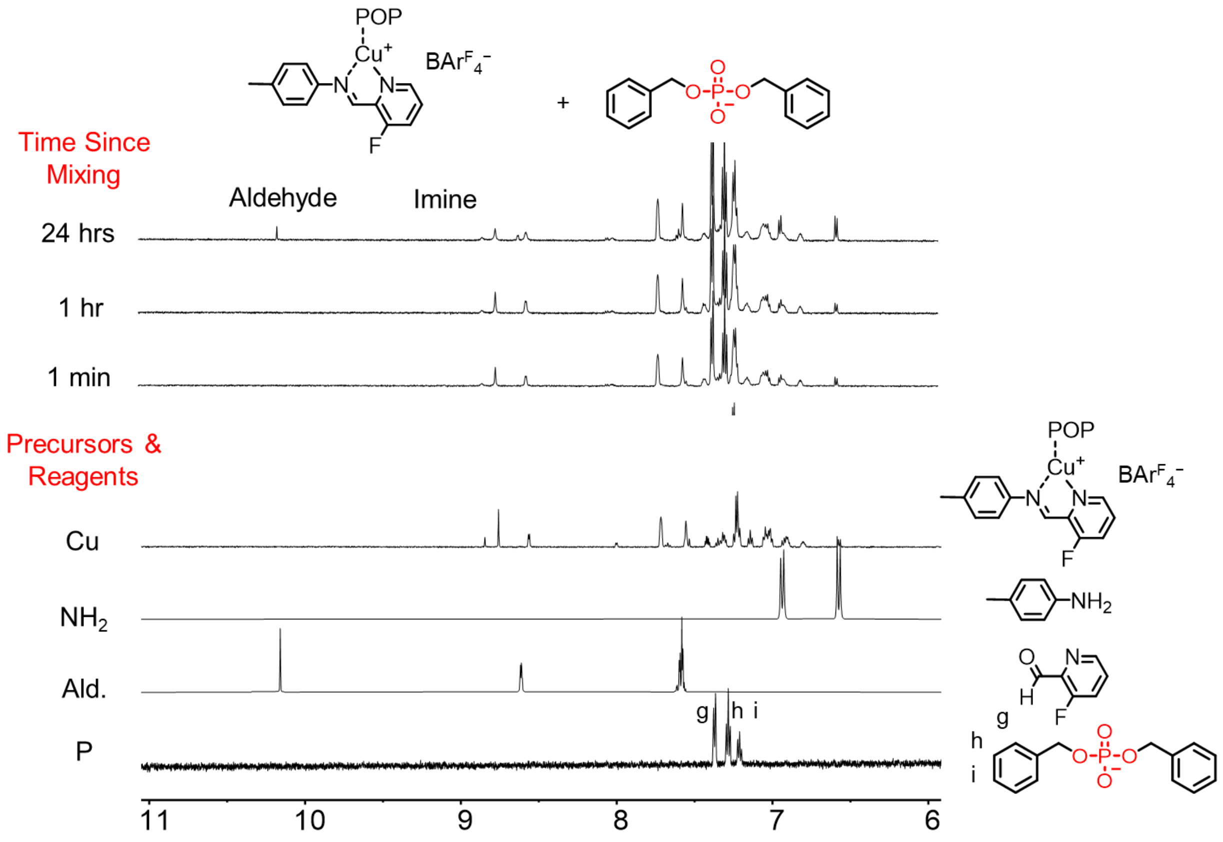

Figure S71. NMR spectra (bottom) of the model Cu^+ complex (1.0 mM), the Schiff base precursors, and dibenzyl phosphate. (Top) NMR spectra from the mixture of the complex (1.0 eq) and the phosphate (1.0 eq) showing the time evolution after mixing in CD_2Cl_2 (600 MHz, 298 K).

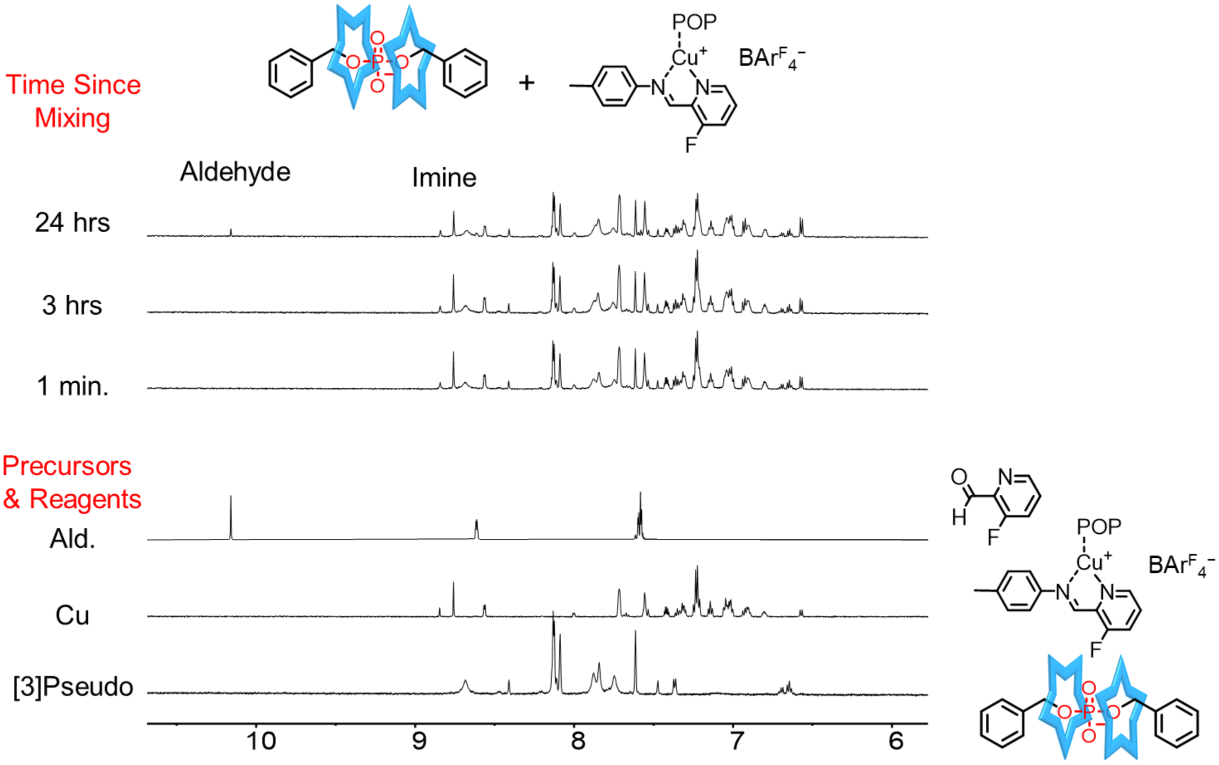

Figure S72. NMR spectra (bottom) of the pyridyl-based aldehyde, the Cu^{+} model complex (1.0 mM), and [3]pseudorotaxane composed of dibenzyl phosphate (0.3 eq) and cyanostar (1.0 equiv.). (Top) NMR spectra from the mixture of the [3]pseudorotaxane and the Cu^{+} model complex showing the time evolution after mixing in CD_2Cl_2 (600 MHz, 298 K).

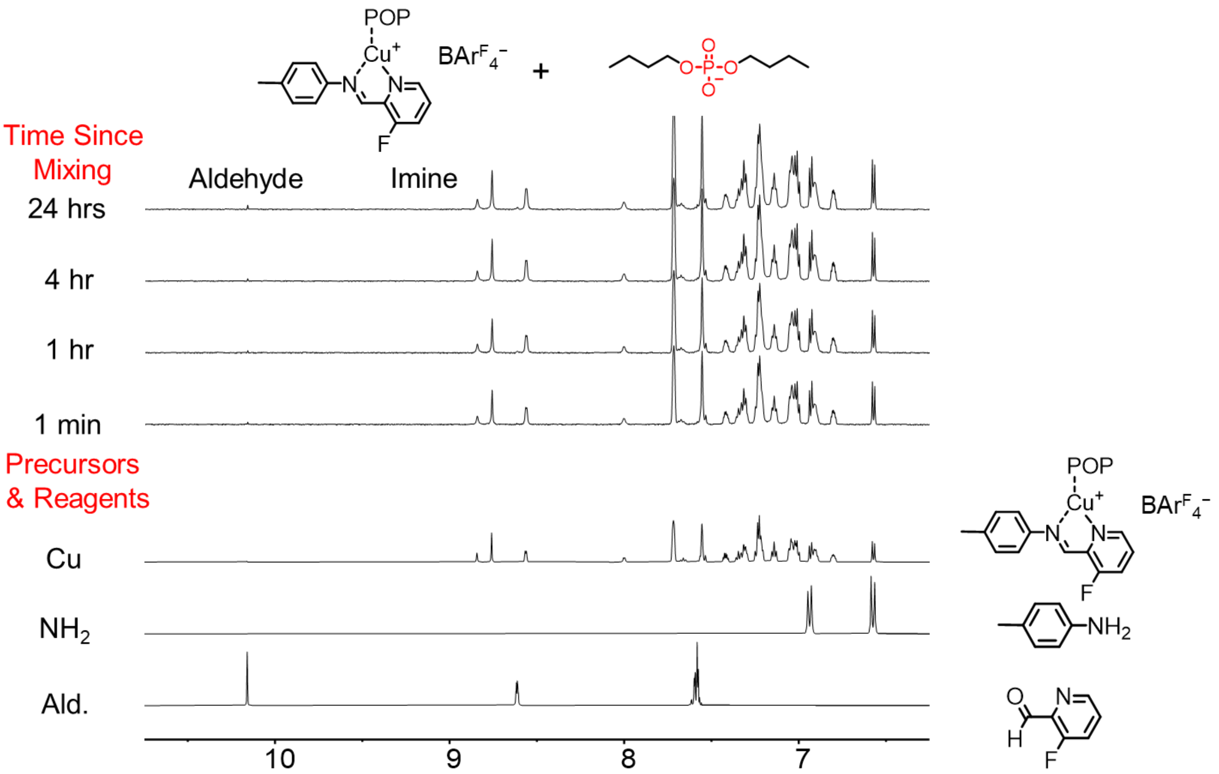

Figure S73. NMR spectra (bottom) of the Cu^{+} model complex (1.0 mM) and the Schiff base precursors. (Top) NMR spectra from the mixture of the Cu^{+} model complex (1.0 equiv.) and dibutyl phosphate (1.0 eq) showing the time evolution after mixing in CD_2Cl_2 (600 MHz, 298 K).

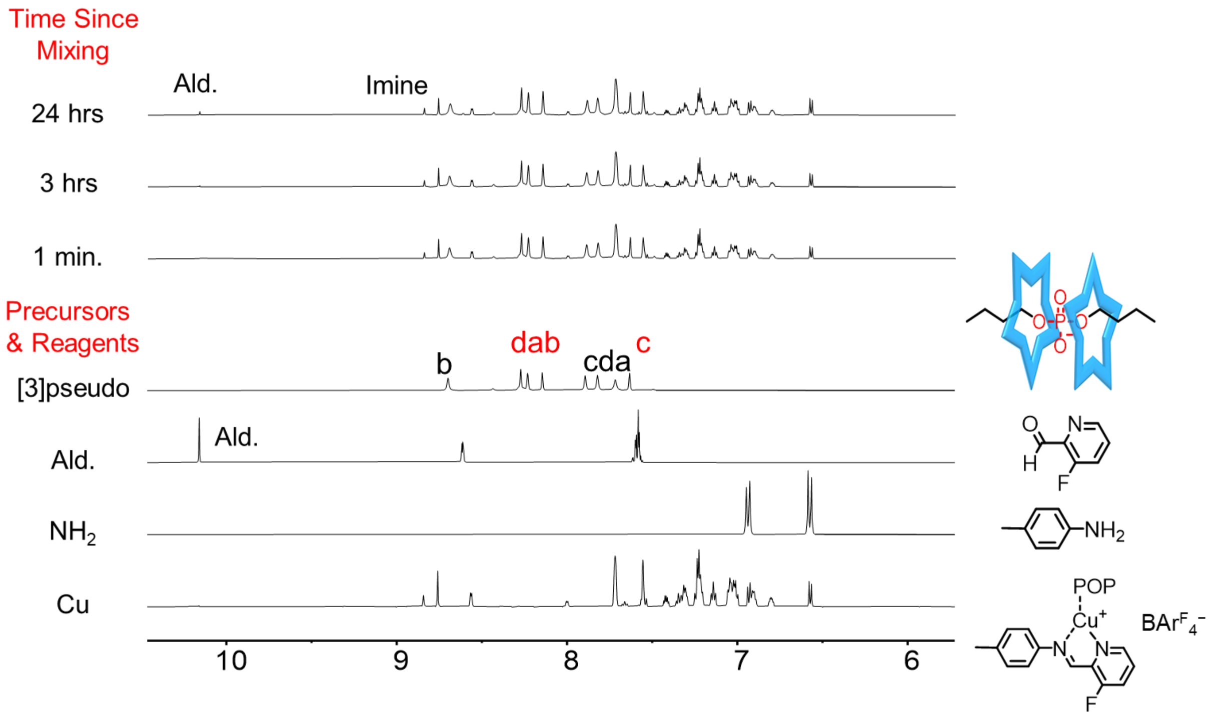

Figure S74. NMR spectra (bottom) of a [3]pseudorotaxane composed of dibutyl phosphate (0.3 eq) and cyanostar (1.0 equiv.), the Schiff base precursors, and the Cu^+ model complex (1.0 mM). (Top) NMR spectra from the mixture of the [3]pseudorotaxane and the Cu^+ model complex showing the time evolution after mixing in CD_2Cl_2 (600 MHz, 298 K).

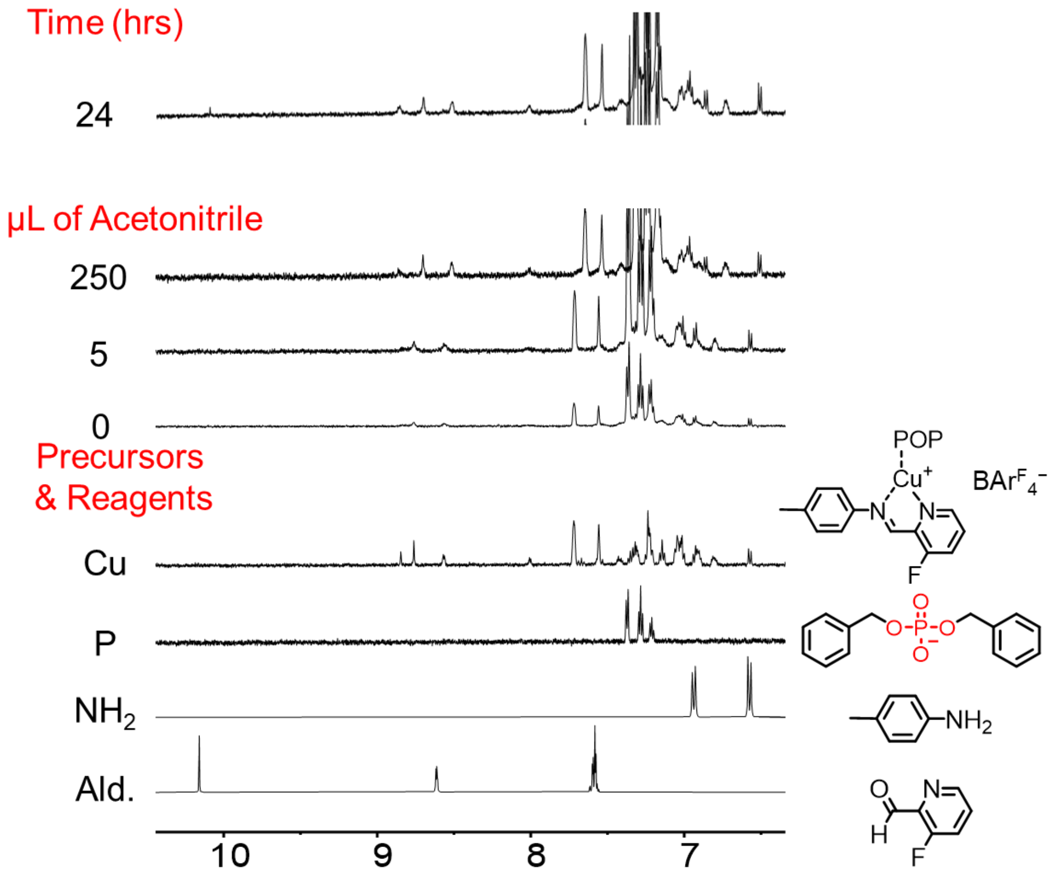

Figure S75. NMR spectra (bottom) of the Cu^{+} model complex (1.0 mM), dibenzyl phosphate, and the Schiff base precursors. NMR spectra (middle) of the mixture of the Cu^{+} model complex and dibenzyl phosphate upon addition of CD_3CN. NMR spectra (top) of the mixture of the Cu^{+} model complex (1.0 mM), dibenzyl phosphate, and CD_3CN showing the time evolution after mixing in CD_2Cl_2 (600 MHz, 298 K).

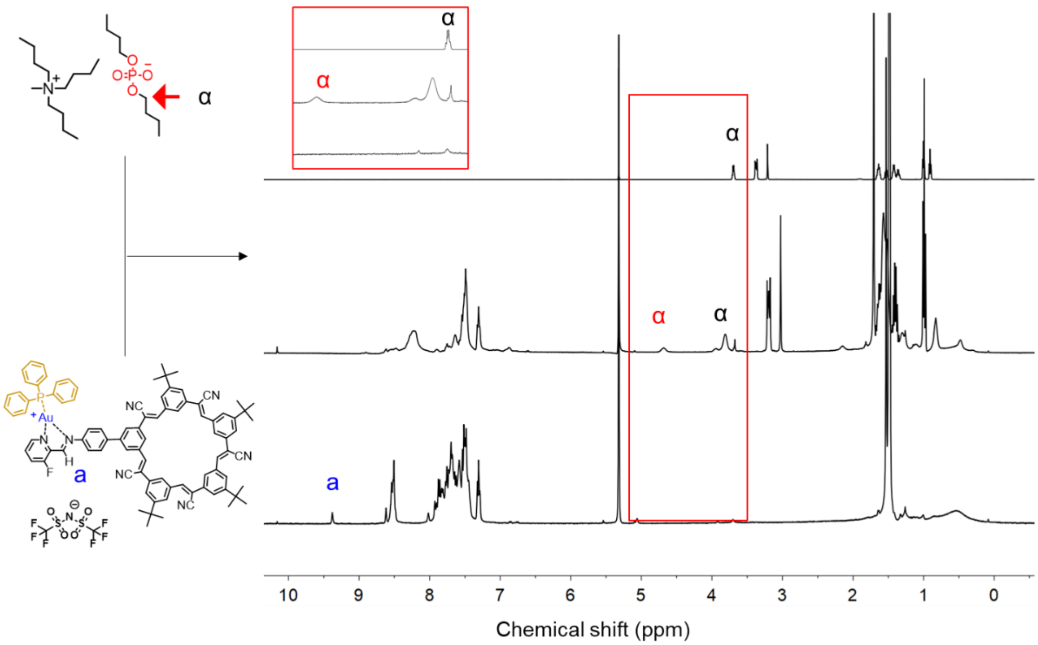

Figure S76. NMR spectra of the di-*n*-butylphosphate anion (top), the mixture of the anion and [**CS**-Au**PPh**$_3$]·NTf$_2$ (middle), and [**CS**-Au**PPh**$_3$]·NTf$_2$ (bottom).

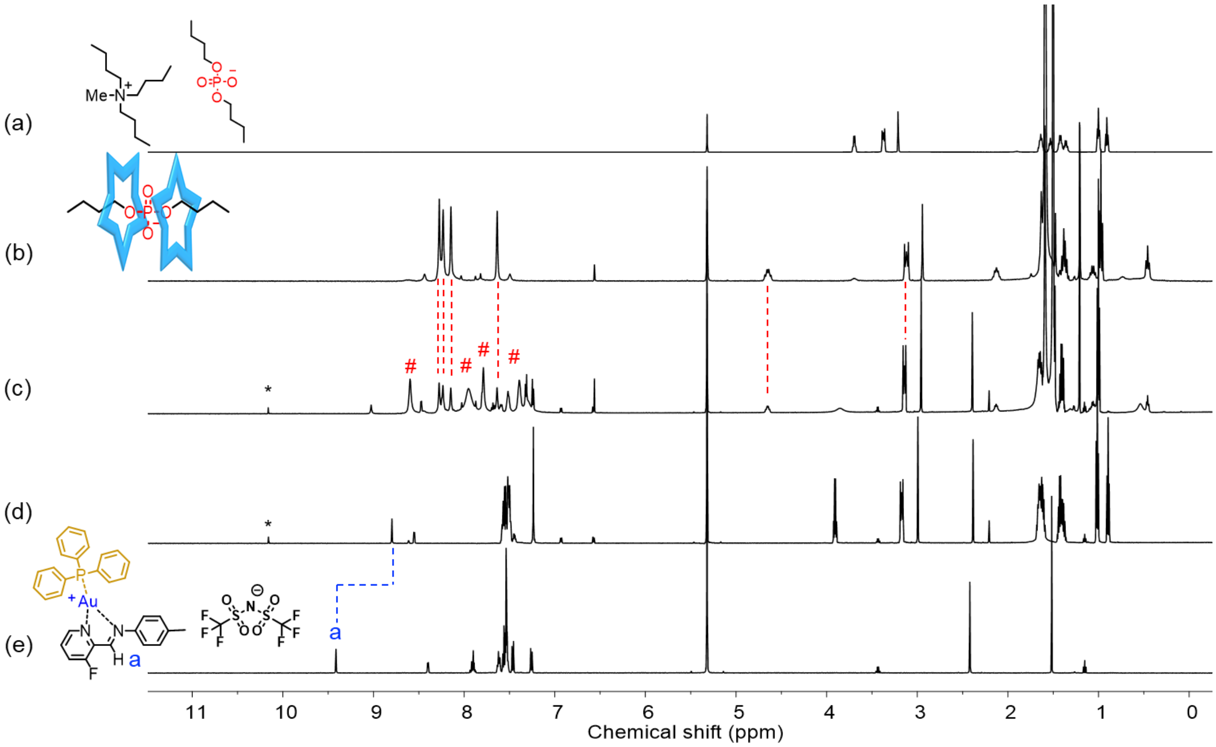

Figure S77. NMR spectra of (a) phosphate anion (**P**$^-$), (b) a [3]pseudorotaxane composed of **P**$^-$ (0.5 eq) and **pCS** (1.0 equiv.), (c) a solution after the addition of **pCS** to a mixture of the model gold(I) complex and **P**$^-$, (d) the mixture of the model gold(I) complex and **P**$^-$, and (e) the model gold(I) complex.

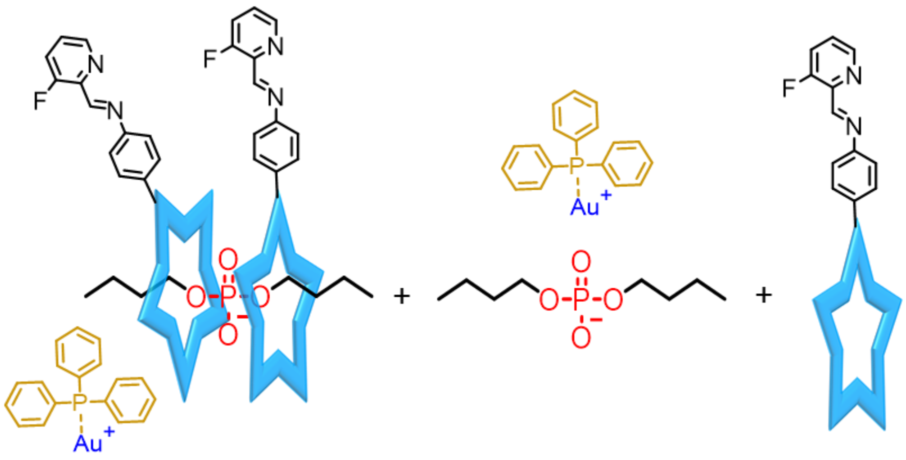

Figure S78. Proposed mixture of products upon addition of dibutyl phosphate to [**PPh**$_3$Au-**CS**]$^+$ NTf$_2^-$

25. Molecular Mechanics

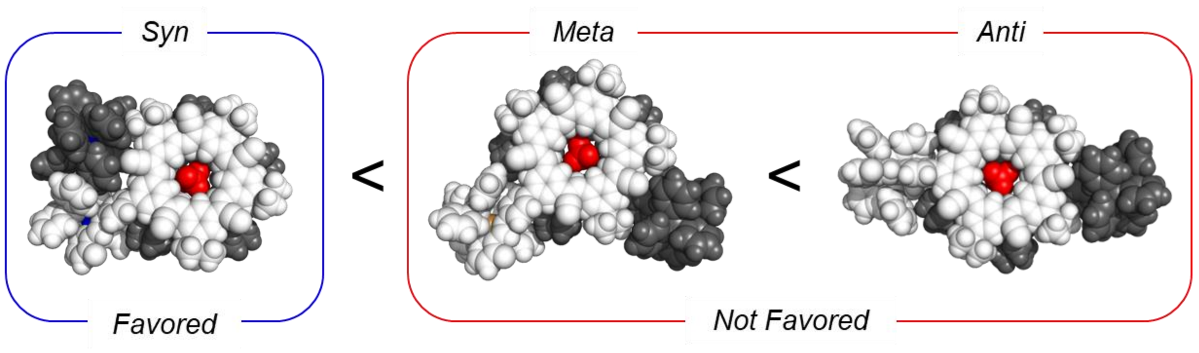

Figure S79. Equilibrium geometry of *syn*, *meta*, and *anti*-configurations were optimized using molecular mechanics in the gas phase (MMFF).

Calculated Energy for *Syn*: 4088 kJ / mol

Calculated Energy for *Meta*: 4104 kJ / mol

Calculated Energy for *Anti*: 4174 kJ / mol

Coordinates for *syn*-Dimer

Atom	x	y	z
C	3.013131	3.310557	-11.3728
C	2.620161	1.860823	-8.99411
C	4.02175	2.486635	-10.8432
C	1.789427	3.363007	-10.6772
C	1.55688	2.606732	-9.52355
C	3.846385	1.739482	-9.65907
H	2.436996	1.298474	-8.08046
C	4.988008	0.934306	-9.10551
C	5.007824	0.54393	-7.80895
H	4.249213	0.930431	-7.14412
C	5.927036	0.395491	-10.0508
N	6.670447	-0.02944	-10.8373
C	8.229983	-1.20716	-6.66806
C	5.539919	-0.8996	-5.89797
C	7.713459	-1.86056	-5.53644
C	7.34443	-0.39802	-7.40478
C	5.99221	-0.2745	-7.06781
C	6.368241	-1.72907	-5.13116
H	4.491492	-0.77313	-5.62879
C	5.898716	-2.39931	-3.87434
C	4.768273	-2.01002	-3.24073
H	4.255502	-1.11335	-3.59163
C	6.570875	-3.60872	-3.4916
N	7.136799	-4.58213	-3.20411
C	4.110935	-3.71768	0.132679
C	2.745235	-2.27783	-1.8629
C	2.732062	-3.45655	0.233463
C	4.782425	-3.21707	-0.99779
C	4.110857	-2.55127	-2.02833
C	2.021117	-2.74022	-0.75256
H	2.241967	-1.73428	-2.66082
C	0.552466	-2.46757	-0.57743
C	-0.08151	-1.51964	-1.29945
H	0.522038	-0.85991	-1.9248
C	-0.20227	-3.40113	0.199655
N	-0.79097	-4.1846	0.821088
C	-3.7152	-0.6992	-0.35699
C	-1.9172	-0.44846	-2.49358
C	-4.11096	-0.15348	-1.59563
C	-2.40087	-1.18826	-0.24611
C	-1.50172	-1.11204	-1.32425
C	-3.23757	-0.02913	-2.68885

H	-1.19436	-0.35021	-3.30511
C	-3.69349	0.704127	-3.92008
C	-2.81759	1.271795	-4.76745
H	-1.76571	1.304761	-4.48858
C	-5.07189	0.543316	-4.28503
N	-6.18994	0.397628	-4.57002
C	-4.43097	3.366695	-7.54611
C	-2.00645	2.068423	-6.9373
C	-3.35569	3.381624	-8.44841
C	-4.241	2.690937	-6.32414
C	-3.06641	1.987179	-6.02827
C	-2.14439	2.741946	-8.15824
H	-1.0841	1.548922	-6.68525
C	-0.98673	2.858285	-9.13657
C	0.292361	2.599981	-8.75502
C	-1.29322	3.070725	-10.5042
N	-1.56596	3.250792	-11.619
C	4.889059	-4.51242	1.198989
C	9.70033	-1.3428	-7.10791
C	-5.76786	4.074928	-7.8375
C	3.209329	4.143816	-12.6541
C	-5.87305	5.325373	-6.94333
H	-5.84327	5.069775	-5.87832
H	-6.81067	5.862903	-7.12389
H	-5.04832	6.020272	-7.13851
C	-6.95996	3.135979	-7.53909
H	-6.87383	2.198407	-8.10109
H	-7.91159	3.60502	-7.81614
H	-7.03699	2.881016	-6.47719
C	-5.92631	4.528791	-9.30679
H	-5.82361	3.685333	-9.99953
H	-6.91295	4.975711	-9.47851
H	-5.18713	5.287843	-9.58292
H	-5.03444	2.742898	-5.58472
H	-3.46248	3.917487	-9.38879
C	3.981035	-5.19739	2.241472
H	3.428267	-4.46903	2.845883
H	4.570197	-5.80616	2.935105
H	3.260014	-5.86538	1.758084
C	5.839014	-3.56492	1.955503
H	5.278969	-2.7751	2.46726
H	6.557617	-3.08148	1.284448
H	6.415493	-4.1057	2.715003
C	5.720401	-5.6358	0.535781
H	6.508421	-5.2444	-0.11593

H	5.084242	-6.29282	-0.06902
H	6.218889	-6.25661	1.289797
C	9.773931	-1.79051	-8.58516
H	10.81237	-1.95067	-8.89838
H	9.350219	-1.04692	-9.26834
H	9.23048	-2.73011	-8.73987
C	10.50733	-2.37537	-6.28753
H	11.53702	-2.45552	-6.6563
H	10.06141	-3.37496	-6.34923
H	10.57035	-2.09406	-5.23024
C	10.40752	0.01874	-6.95551
H	9.934326	0.80168	-7.55827
H	11.45605	-0.04269	-7.26928
H	10.39386	0.350254	-5.91152
C	2.077022	3.846652	-13.6629
H	2.235019	4.385667	-14.6046
H	1.093654	4.149794	-13.2885
H	2.027414	2.776364	-13.8957
C	4.542816	3.865096	-13.3854
H	4.621443	4.45888	-14.3041
H	4.631688	2.810474	-13.6714
H	5.408249	4.125137	-12.7654
C	3.187284	5.642893	-12.2935
H	3.319286	6.266483	-13.1852
H	3.99507	5.888663	-11.5951
H	2.243082	5.94217	-11.8256
H	1.016737	4.029664	-11.0492
H	4.985013	2.444771	-11.3466
H	7.740186	0.155959	-8.25075
H	8.380424	-2.47133	-4.93266
H	5.859611	-3.34492	-1.05191
H	2.1964	-3.80124	1.114706
H	-2.08215	-1.59376	0.710861
H	-5.11608	0.26406	-1.66772
H	0.489845	2.41975	-7.69848
H	-4.92967	0.754082	3.883639
C	-5.24978	0.22291	2.99768
C	-6.01442	-0.97247	0.615027
C	-6.59891	-0.04021	2.784791
C	-4.29156	-0.07642	2.013972
C	-4.6619	-0.63119	0.780337
C	-6.96898	-0.67498	1.598149
H	-3.2603	0.207497	2.204342
H	-8.00457	-0.9329	1.404718
H	-6.35521	-1.43057	-0.31182

C	-9.68798	1.268326	4.474314
C	-11.252	2.523031	6.274614
C	-10.995	1.606241	4.117683
N	-9.24898	1.540505	5.642761
C	-9.93778	2.137855	6.526791
C	-11.8023	2.247539	5.035953
H	-9.46814	2.318706	7.489183
H	-12.8185	2.522283	4.78548
H	-11.8289	3.023833	7.044767
N	-7.51554	0.372464	3.679305
C	-8.81366	0.625095	3.470596
H	-9.28681	0.418994	2.496199
Cu	-7.15433	0.828916	5.929985
P	-7.16255	-0.95699	7.545546
P	-5.67439	2.645506	6.470557
C	-8.83181	-1.01645	8.449777
C	-11.3163	-1.16169	9.715966
C	-8.90646	-1.01679	9.843781
C	-10.001	-1.11637	7.692199
C	-11.2419	-1.1815	8.324226
C	-10.149	-1.08308	10.47512
H	-8.00499	-0.96848	10.44692
H	-9.94573	-1.13852	6.606064
H	-12.1476	-1.2495	7.727944
H	-10.2061	-1.07765	11.56012
H	-12.2832	-1.21457	10.20827
C	-6.97235	-2.66277	6.77288
C	-6.8069	-5.2265	5.666218
C	-6.85845	-3.78322	7.601796
C	-7.03459	-2.83707	5.39213
C	-6.94577	-4.11481	4.838753
C	-6.77083	-5.06058	7.048558
H	-6.84429	-3.66741	8.68255
H	-7.16095	-1.98773	4.731462
H	-6.9763	-4.23774	3.759236
H	-6.67636	-5.92608	7.696759
H	-6.72724	-6.21856	5.231954
C	-3.89611	2.076682	6.759746
C	-1.24743	1.296498	7.201694
C	-3.46099	0.811133	6.366014
C	-2.99652	2.949615	7.381496
C	-1.67572	2.560043	7.601535
C	-2.13847	0.422459	6.583393
H	-4.13945	0.108977	5.89699
H	-3.31695	3.940628	7.689078

H	-0.97841	3.243341	8.077482
H	-1.80025	-0.56346	6.272461
H	-0.21861	0.990984	7.366766
C	-5.7421	3.890927	5.042054
C	-5.8919	5.854552	3.063837
C	-6.98365	4.402235	4.652058
C	-4.579	4.345412	4.42085
C	-4.65488	5.327676	3.433279
C	-7.05771	5.383257	3.66458
H	-7.8952	4.042514	5.122
H	-3.60611	3.946248	4.694464
H	-3.74585	5.675484	2.948903
H	-8.02426	5.777107	3.363351
H	-5.94751	6.619276	2.294365
C	-5.77445	-0.52844	8.749908
C	-3.67955	0.262235	10.42186
C	-5.80996	0.722347	9.383045
C	-4.68104	-1.36991	8.944348
C	-3.63207	-0.96971	9.772962
C	-4.77194	1.107949	10.23444
H	-4.61632	-2.32348	8.432638
H	-2.76109	-1.60866	9.894406
H	-4.78521	2.06597	10.74422
H	-2.85362	0.572505	11.05632
C	-6.26581	3.539428	8.043912
C	-7.18766	4.933644	10.29929
C	-6.17666	4.936328	8.104498
C	-6.78338	2.85011	9.140549
C	-7.27139	3.543858	10.25206
C	-6.63269	5.628974	9.227696
H	-5.75767	5.49851	7.273096
H	-7.71051	2.996244	11.0816
H	-6.56337	6.713565	9.258438
H	-7.55627	5.473092	11.16738
O	-6.9275	1.489435	9.137403
F	-11.484	1.328012	2.899872
C	7.176774	4.463309	-8.53651
C	5.175835	4.143845	-6.58319
C	5.937527	5.118331	-8.6454
C	7.381226	3.65109	-7.40448
C	6.421084	3.526384	-6.39523
C	4.919071	4.981677	-7.67887
H	4.426229	4.01829	-5.80284
C	3.588599	5.636943	-7.89626
C	2.482583	5.222586	-7.23662

H	2.545222	4.317651	-6.63143
C	3.574625	6.855243	-8.65179
N	3.585776	7.838797	-9.26949
C	-0.79431	6.894577	-8.32274
C	0.286868	5.435967	-6.16506
C	-1.54777	6.623899	-7.15925
C	0.521928	6.419304	-8.35086
C	1.087222	5.742248	-7.2637
C	-1.03657	5.895011	-6.06399
H	0.739097	4.884847	-5.34212
C	-1.90843	5.59342	-4.87848
C	-1.58908	4.620099	-3.99298
H	-0.7376	3.97514	-4.2129
C	-2.98396	6.500215	-4.60627
N	-3.84872	7.250859	-4.41227
C	-4.22512	3.861939	-1.30269
C	-1.48693	3.495076	-1.81022
C	-3.37578	3.27179	-0.34658
C	-3.63665	4.314638	-2.49941
C	-2.26776	4.182911	-2.75108
C	-1.99298	3.081127	-0.57156
H	-0.42775	3.365435	-2.03301
C	-1.16617	2.355671	0.454066
C	0.00877	1.790604	0.135684
H	0.292052	1.750716	-0.91713
C	-1.54486	2.510548	1.827201
N	-1.85526	2.644583	2.937361
C	1.688525	-0.31381	2.862487
C	2.309222	1.011805	0.484113
C	3.006246	-0.28533	2.372585
C	0.689916	0.360359	2.1439
C	0.994442	1.072294	0.974911
C	3.348196	0.374269	1.179752
H	2.522699	1.543894	-0.4433
C	4.755749	0.31221	0.659828
C	5.045939	0.587901	-0.63019
H	4.224099	0.744027	-1.32925
C	5.80357	0.161733	1.627843
N	6.638677	0.036845	2.42567
C	8.685757	-0.05586	-1.67611
C	6.415833	1.378514	-2.5143
C	8.724903	0.77713	-2.80813
C	7.471332	-0.12622	-0.9667
C	6.350263	0.626587	-1.33253
C	7.604878	1.513416	-3.24835

H	5.52532	1.931746	-2.80924
C	7.692198	2.320747	-4.50874
C	6.57856	2.704876	-5.17276
C	8.9734	2.856731	-4.86422
N	10.01903	3.279633	-5.14185
C	-5.73775	4.051938	-1.06683
C	-1.41158	7.703163	-9.47762
C	9.898417	-0.88504	-1.21091
C	8.284587	4.597463	-9.59928
C	9.582087	-2.38593	-1.37378
H	9.374544	-2.63032	-2.42161
H	8.711177	-2.69264	-0.78389
H	10.42657	-3.00544	-1.05019
C	10.20753	-0.58911	0.273962
H	10.38184	0.481402	0.434412
H	11.10391	-1.12629	0.605735
H	9.393195	-0.89503	0.939193
C	11.1949	-0.5989	-2.00296
H	11.48409	0.456247	-1.93313
H	11.08796	-0.85458	-3.06321
H	12.03284	-1.19169	-1.61676
H	7.410179	-0.80565	-0.12127
H	9.644028	0.833319	-3.38632
C	-6.27435	3.313409	0.179508
H	-6.1008	2.234437	0.107166
H	-7.35444	3.462909	0.295844
H	-5.80482	3.669634	1.100371
C	-6.55695	3.523739	-2.26618
H	-6.35778	2.461058	-2.43473
H	-6.33912	4.055997	-3.19762
H	-7.63326	3.633326	-2.08722
C	-6.02382	5.55709	-0.89139
H	-5.77972	6.126926	-1.79532
H	-5.43194	5.979259	-0.07171
H	-7.08213	5.738016	-0.66953
C	-0.49363	7.825381	-10.7145
H	-0.98124	8.393732	-11.5155
H	-0.24048	6.839975	-11.1232
H	0.43962	8.347493	-10.4744
C	-1.71613	9.133311	-8.98338
H	-2.13099	9.75044	-9.78882
H	-0.80743	9.627013	-8.6187
H	-2.44358	9.139943	-8.16384
C	-2.72159	7.044418	-9.95652
H	-3.15548	7.593052	-10.8006

H	-3.48407	7.015548	-9.17191
H	-2.5449	6.01536	-10.2865
C	9.608005	5.040439	-8.93538
H	10.39218	5.199974	-9.68489
H	9.992563	4.294584	-8.23179
H	9.477694	5.979321	-8.38434
C	7.971003	5.632349	-10.7042
H	8.799662	5.711607	-11.4182
H	7.807319	6.631669	-10.2842
H	7.08157	5.353932	-11.2807
C	8.496	3.235787	-10.2916
H	9.284313	3.296373	-11.0509
H	7.579129	2.905926	-10.7925
H	8.787532	2.451597	-9.58421
H	8.312426	3.097315	-7.33185
H	5.74262	5.730821	-9.52231
H	1.116494	6.57002	-9.24353
H	-2.57434	6.985059	-7.12223
H	-4.2789	4.768457	-3.24727
H	-3.8106	2.920306	0.588932
H	-0.32756	0.311047	2.523088
H	3.763952	-0.82746	2.938837
H	5.616094	2.310428	-4.84696
H	-0.13403	-3.83939	5.285445
C	0.448365	-2.92878	5.318165
C	1.903904	-0.56356	5.329342
C	0.963109	-2.46331	6.523723
C	0.648029	-2.2166	4.124686
C	1.393449	-1.03067	4.110906
C	1.687335	-1.268	6.519635
H	0.225531	-2.61462	3.205272
H	2.133337	-0.88335	7.430178
H	2.489874	0.352957	5.360085
C	0.817013	-3.63086	10.09244
C	0.662466	-5.25371	12.23396
C	1.009693	-3.09225	11.36612
N	0.564857	-4.8754	9.945863
C	0.48388	-5.68437	10.92148
C	0.932739	-3.91834	12.46846
H	0.256139	-6.72014	10.69586
H	1.078439	-3.52841	13.46726
H	0.584123	-5.96047	13.05324
N	0.795605	-3.16543	7.65781
C	0.904059	-2.73739	8.920904
H	1.069039	-1.67034	9.146493

Cu	0.281039	-5.41775	7.797276
P	-1.94604	-6.19972	7.343931
P	1.968174	-6.93377	6.996728
C	-3.07786	-5.89604	8.832039
C	-4.72009	-5.46816	11.05644
C	-4.11328	-6.78726	9.1259
C	-2.87338	-4.78842	9.654299
C	-3.68852	-4.57804	10.76716
C	-4.93238	-6.57328	10.23561
H	-4.29264	-7.65387	8.49717
H	-2.07436	-4.08568	9.434741
H	-3.51724	-3.71748	11.40832
H	-5.7359	-7.26988	10.45892
H	-5.35616	-5.30029	11.92101
C	-2.54976	-5.23013	5.840964
C	-3.34875	-3.72397	3.62254
C	-3.12076	-3.96892	6.014603
C	-2.35431	-5.7196	4.54775
C	-2.76947	-4.9766	3.443867
C	-3.51399	-3.21636	4.907855
H	-3.24662	-3.55538	7.008452
H	-1.8649	-6.67447	4.380537
H	-2.6233	-5.36739	2.440058
H	-3.96055	-2.23666	5.046779
H	-3.66486	-3.14277	2.760707
C	1.478597	-7.55362	5.284324
C	0.704249	-8.4145	2.741913
C	1.22048	-6.62374	4.276093
C	1.375224	-8.91762	5.008208
C	0.983837	-9.3466	3.739522
C	0.825635	-7.05309	3.009739
H	1.329335	-5.56074	4.469909
H	1.587595	-9.65706	5.77417
H	0.891899	-10.4088	3.530167
H	0.609691	-6.32347	2.232296
H	0.394521	-8.74599	1.754357
C	3.628814	-6.04122	6.875503
C	6.082269	-4.71861	6.695336
C	3.942163	-5.02162	7.774673
C	4.553524	-6.40884	5.894663
C	5.778258	-5.74695	5.805037
C	5.164991	-4.35764	7.680882
H	3.233336	-4.72851	8.543981
H	4.323289	-7.2042	5.190754
H	6.491069	-6.02505	5.033268

H	5.395866	-3.55159	8.37214
H	7.030286	-4.19321	6.617253
C	-1.94579	-8.07259	7.003697
C	-1.97611	-10.812	6.442276
C	-1.0471	-8.92565	7.654714
C	-2.88495	-8.60584	6.110658
C	-2.89221	-9.9687	5.822443
C	-1.06428	-10.2954	7.364803
H	-3.61933	-7.96123	5.633603
H	-3.61342	-10.3704	5.115227
H	-0.38573	-10.9869	7.853137
H	-1.98035	-11.8758	6.22028
C	2.118644	-8.37926	8.201635
C	2.38677	-10.4263	10.09691
C	3.376876	-8.91977	8.485144
C	0.993071	-8.90295	8.84146
C	1.129408	-9.89512	9.816411
C	3.508304	-9.9448	9.423915
H	4.26678	-8.53504	7.993752
H	0.255336	-10.2537	10.35328
H	4.492098	-10.3521	9.644484
H	2.494389	-11.2069	10.84515
O	-0.24636	-8.35841	8.61945
F	1.268156	-1.78788	11.5438
B	1.909137	1.461484	-4.14964
F	3.10889	1.327617	-4.42656
F	1.372211	0.36726	-4.12684
F	1.362252	2.173356	-5.00715
F	1.800116	1.987074	-3.05283

<u>Coordinates for *anti*-Dimer</u>

Atom	x	y	z
C	2.423527	-4.8745	4.570917
C	0.944065	-3.70076	2.506713
C	1.03998	-4.65225	4.700607
C	3.021804	-4.56668	3.333139
C	2.282086	-4.03975	2.272527
C	0.2676	-4.08673	3.672793
H	0.398955	-3.23682	1.68506
C	-1.19008	-3.81764	3.890441
C	-1.83786	-2.9222	3.113085
H	-1.24468	-2.2857	2.454711
C	-1.90691	-4.69694	4.775239

N	-2.46904	-5.40667	5.50639
C	-5.48542	-2.26551	4.057055
C	-3.7248	-1.92306	1.897233
C	-5.92433	-1.73342	2.837645
C	-4.12952	-2.61916	4.162208
C	-3.25167	-2.50257	3.084927
C	-5.06318	-1.53084	1.74624
H	-3.02315	-1.80701	1.070568
C	-5.59862	-0.83521	0.54122
C	-4.75812	-0.2631	-0.35176
H	-3.69605	-0.17278	-0.09582
C	-6.98002	-1.06089	0.210839
N	-8.10247	-1.27182	-0.00519
C	-6.44247	1.919955	-2.99258
C	-4.04867	0.52605	-2.58088
C	-5.46452	1.843845	-4.00034
C	-6.20815	1.210132	-1.80127
C	-5.0662	0.434637	-1.62152
C	-4.24862	1.162568	-3.82014
H	-3.13526	-0.03738	-2.3893
C	-3.1949	1.204893	-4.89059
C	-1.91669	0.903868	-4.57328
H	-1.66792	0.800255	-3.51788
C	-3.60546	1.386448	-6.25627
N	-3.95567	1.566395	-7.35084
C	0.736898	1.350007	-7.28324
C	0.349752	0.033787	-4.84537
C	1.715888	0.500839	-6.74347
C	-0.4638	1.507907	-6.57952
C	-0.68936	0.818221	-5.38476
C	1.54545	-0.18632	-5.53314
H	0.166355	-0.48681	-3.90369
C	2.69538	-0.9926	-5.00182
C	2.810383	-1.31563	-3.75403
H	2.078745	-0.82611	-3.01528
C	3.523807	-1.60537	-5.98413
N	4.171255	-2.02633	-6.84539
C	6.054319	-2.93005	-2.64465
C	3.411351	-2.66533	-1.75759
C	5.612649	-3.59075	-1.48328
C	5.116945	-2.17692	-3.37866
C	3.770324	-2.09174	-3.01782
C	4.291014	-3.48093	-1.01705
H	2.379725	-2.53923	-1.42942
C	3.938141	-4.08602	0.308723

C	2.873	-3.61518	0.989027
C	4.543171	-5.35201	0.643287
N	5.069912	-6.36364	0.874073
C	-7.73246	2.741253	-3.15152
C	-6.4298	-2.46761	5.249101
C	7.514704	-3.0052	-3.12346
C	3.264588	-5.46765	5.712749
C	8.175862	-1.61957	-3.00231
H	8.174113	-1.28004	-1.96247
H	7.665664	-0.86187	-3.60353
H	9.219054	-1.64475	-3.32995
C	7.551574	-3.45854	-4.59662
H	7.025648	-4.41123	-4.72606
H	8.580517	-3.58682	-4.94134
H	7.082819	-2.73232	-5.26623
C	8.379827	-3.99639	-2.31561
H	7.963999	-5.0091	-2.35089
H	9.398643	-4.04537	-2.71003
H	8.463872	-3.6949	-1.26552
H	5.467899	-1.62266	-4.24414
H	6.318141	-4.17383	-0.89958
C	-7.88757	3.383334	-4.54752
H	-7.07882	4.092926	-4.75554
H	-8.82919	3.936504	-4.62693
H	-7.88931	2.625417	-5.3382
C	-7.7583	3.884516	-2.11411
H	-6.89336	4.546185	-2.23263
H	-7.74673	3.513465	-1.08341
H	-8.66054	4.496817	-2.22647
C	-8.95189	1.822657	-2.93042
H	-8.99123	1.422915	-1.91142
H	-8.92658	0.969083	-3.61676
H	-9.88987	2.362352	-3.09672
C	-6.40909	-3.95228	5.666236
H	-7.09221	-4.13601	6.501578
H	-5.41536	-4.28153	5.983433
H	-6.71223	-4.59941	4.83452
C	-7.90111	-2.10459	4.950371
H	-8.53631	-2.2744	5.825942
H	-8.3054	-2.71123	4.131438
H	-8.00941	-1.04966	4.676567
C	-5.96434	-1.58071	6.419223
H	-6.61528	-1.69923	7.291175
H	-5.97972	-0.52332	6.137768
H	-4.94383	-1.81482	6.738483

C	3.77738	-6.84853	5.265925
H	4.345269	-7.33485	6.064578
H	4.435586	-6.77668	4.393398
H	2.945799	-7.50707	4.991263
C	2.473935	-5.65365	7.025017
H	3.112494	-6.05297	7.816176
H	1.644444	-6.3556	6.895762
H	2.069331	-4.70368	7.392345
C	4.468297	-4.56512	6.048158
H	5.055938	-4.98056	6.874235
H	4.132398	-3.57476	6.361424
H	5.150628	-4.43675	5.202917
H	4.091348	-4.71237	3.214331
H	0.549213	-4.8936	5.639584
H	-3.76568	-2.98576	5.116652
H	-6.96445	-1.43769	2.740749
H	-6.92808	1.294095	-0.99177
H	-5.63683	2.354708	-4.94148
H	-1.21611	2.174428	-6.98721
H	2.658172	0.413385	-7.28017
H	2.425254	-2.6768	0.65848
H	0.884128	5.057393	-10.0879
C	1.102785	4.007232	-9.97233
C	1.601209	1.310613	-9.61497
C	1.71923	3.29624	-10.9993
C	0.757952	3.38562	-8.76211
C	1.012322	2.025246	-8.56004
C	1.957718	1.935415	-10.8106
H	0.307476	3.98869	-7.97818
H	2.420266	1.325196	-11.5759
H	1.799722	0.247158	-9.51516
C	3.043417	4.223482	-14.3999
C	3.564473	5.656055	-16.6134
C	3.718857	3.6131	-15.4573
N	2.681795	5.446339	-14.4774
C	2.903395	6.16667	-15.4993
C	3.988721	4.342137	-16.5949
H	2.554832	7.1815	-15.474
H	4.482897	3.903279	-17.4114
H	3.730042	6.267654	-17.4607
N	2.077029	3.913099	-12.139
C	2.737969	3.42656	-13.1966
H	3.092101	2.390501	-13.2236
Cu	1.638432	6.121901	-12.6533
P	-0.66965	6.651134	-13.0663

P	2.830445	7.836726	-11.4655
C	-1.19045	5.986314	-14.7562
C	-1.94567	4.975276	-17.2484
C	-2.07838	6.70115	-15.5619
C	-0.69576	4.756113	-15.1957
C	-1.07046	4.253118	-16.4406
C	-2.45356	6.195451	-16.8067
H	-2.48589	7.646384	-15.2311
H	-0.0176	4.18635	-14.5692
H	-0.67531	3.311066	-16.7716
H	-3.14102	6.746575	-17.419
H	-2.22922	4.595025	-18.2027
C	-1.68073	5.812143	-11.707
C	-3.08303	4.539748	-9.65559
C	-2.33406	4.602025	-11.9496
C	-1.73025	6.382603	-10.4322
C	-2.4325	5.747672	-9.40976
C	-3.03343	3.967231	-10.9243
H	-2.28954	4.131137	-12.9253
H	-1.21165	7.314401	-10.2229
H	-2.45636	6.191057	-8.42035
H	-3.52604	3.018869	-11.1095
H	-3.61883	4.032685	-8.86113
C	1.774253	8.471546	-10.0378
C	0.216617	9.362618	-7.89163
C	1.59251	7.660137	-8.91832
C	1.189282	9.738625	-10.0681
C	0.405849	10.18071	-9.00009
C	0.810571	8.105689	-7.85594
H	2.073904	6.694178	-8.86199
H	1.340385	10.39544	-10.9154
H	-0.05528	11.16253	-9.02787
H	0.666822	7.481535	-6.99585
H	-0.38319	9.701667	-7.05649
C	4.444729	7.130277	-10.7622
C	6.808327	6.074703	-9.71211
C	5.096905	6.086472	-11.4187
C	4.988354	7.653217	-9.58352
C	6.16534	7.122823	-9.05796
C	6.275388	5.558312	-10.8925
H	4.694279	5.667197	-12.336
H	4.49767	8.452208	-9.04953
H	6.542499	7.51218	-8.13371
H	6.754925	4.750527	-11.4144
H	7.696391	5.668655	-9.30327

C	-0.95363	8.525075	-13.0239
C	-1.4513	11.27197	-12.8956
C	0.041403	9.427667	-13.4173
C	-2.19843	9.003173	-12.5901
C	-2.44383	10.3723	-12.5213
C	-0.21698	10.80256	-13.351
H	-2.98363	8.308493	-12.3026
H	-3.40757	10.73494	-12.1757
H	0.523578	11.53425	-13.6503
H	-1.63861	12.34125	-12.8414
C	3.235479	9.243715	-12.6612
C	3.979455	11.20295	-14.5214
C	4.438443	9.942219	-12.5118
C	2.381967	9.575447	-13.718
C	2.774879	10.51884	-14.6721
C	4.804044	10.92345	-13.4339
H	5.098438	9.722092	-11.6862
H	2.139098	10.71296	-15.5293
H	5.730955	11.44366	-13.302
H	4.270138	11.9312	-15.2516
O	1.208373	8.888593	-13.9113
F	4.099273	2.3425	-15.398
C	4.71649	2.612498	-4.7192
C	2.962826	2.532326	-2.52793
C	3.518525	3.34696	-4.71969
C	5.039551	1.892061	-3.55607
C	4.201341	1.881466	-2.4425
C	2.634113	3.343641	-3.62287
H	2.316818	2.509145	-1.65636
C	1.329431	4.035249	-3.73856
C	0.288362	3.673262	-2.95841
H	0.369482	2.760507	-2.38931
C	1.319451	5.269516	-4.46195
N	1.360499	6.264204	-5.05169
C	-3.02329	5.416492	-3.74169
C	-1.78414	3.988318	-1.67994
C	-3.67184	5.20093	-2.51328
C	-1.72752	4.891138	-3.90186
C	-1.07136	4.229195	-2.86452
C	-3.08471	4.467714	-1.46884
H	-1.27222	3.441232	-0.89022
C	-3.85891	4.190504	-0.2236
C	-3.49168	3.217679	0.64305
H	-2.70282	2.52729	0.34997
C	-4.84266	5.173348	0.161276

N	-5.64152	5.974079	0.434432
C	-6.0011	2.541873	3.445599
C	-3.29937	2.135604	2.845013
C	-5.13222	1.965424	4.384502
C	-5.46115	2.945568	2.212702
C	-4.10264	2.828926	1.930847
C	-3.78059	1.723669	4.103959
H	-2.24394	2.028741	2.594391
C	-2.92241	1.00412	5.101279
C	-1.75682	0.452975	4.707745
H	-1.55507	0.410303	3.638513
C	-3.27726	1.073626	6.48926
N	-3.59601	1.117186	7.606353
C	0.206435	-1.54715	7.304325
C	0.558804	-0.27863	4.84395
C	1.469866	-1.47871	6.69848
C	-0.88429	-0.94868	6.66309
C	-0.70985	-0.2656	5.457426
C	1.675468	-0.85001	5.463908
H	0.675319	0.25339	3.896935
C	3.026341	-0.91866	4.839612
C	3.198062	-0.71297	3.516618
H	2.320851	-0.64056	2.872351
C	4.143804	-0.90491	5.73934
N	5.002171	-0.90895	6.521854
C	6.697422	-1.53346	2.244483
C	4.448312	-0.02456	1.511095
C	6.695448	-0.74956	1.078953
C	5.545903	-1.50873	3.044852
C	4.451065	-0.70068	2.737417
C	5.581768	0.005596	0.676763
H	3.564054	0.56308	1.265652
C	5.616045	0.734673	-0.63353
C	4.461539	1.128006	-1.20506
C	6.896254	1.147208	-1.14534
N	7.954496	1.424691	-1.54337
C	-7.49945	2.725873	3.715179
C	-3.6954	6.172	-4.89704
C	7.89266	-2.41337	2.637713
C	5.670875	2.586762	-5.92333
C	7.513016	-3.89701	2.466338
H	7.236131	-4.11796	1.429863
H	6.666072	-4.18244	3.097434
H	8.350754	-4.55051	2.729821
C	8.28064	-2.14589	4.106939

H	8.471351	-1.0801	4.273367
H	9.182589	-2.70027	4.383763
H	7.4969	-2.44753	4.80818
C	9.150054	-2.14921	1.781945
H	9.456435	-1.09962	1.844418
H	8.978268	-2.39381	0.727867
H	9.994398	-2.75974	2.115434
H	5.493539	-2.17082	3.904382
H	7.572542	-0.74974	0.440627
C	-7.92539	2.320827	5.142777
H	-7.71873	1.262884	5.338566
H	-8.99911	2.474112	5.294982
H	-7.40068	2.914185	5.900481
C	-8.29917	1.857049	2.723528
H	-8.0256	0.800039	2.814388
H	-8.12544	2.148041	1.681403
H	-9.3761	1.939792	2.908443
C	-7.87687	4.20903	3.526312
H	-7.72183	4.550462	2.497001
H	-7.27541	4.853372	4.17807
H	-8.93216	4.379942	3.76426
C	-2.74843	7.262335	-5.44236
H	-3.23702	7.851282	-6.22273
H	-1.84174	6.843795	-5.8855
H	-2.43805	7.948763	-4.64601
C	-5.01013	6.875671	-4.49516
H	-5.4361	7.426799	-5.33809
H	-4.84425	7.590465	-3.681
H	-5.77268	6.159627	-4.17081
C	-4.02437	5.167927	-6.01515
H	-4.51154	5.661855	-6.85958
H	-4.70132	4.387544	-5.65872
H	-3.12841	4.66915	-6.39333
C	7.000361	3.249982	-5.51615
H	7.69551	3.280898	-6.3572
H	7.497693	2.70795	-4.70439
H	6.841343	4.272547	-5.16974
C	5.12162	3.332329	-7.15882
H	5.817242	3.271513	-7.99367
H	4.965923	4.390273	-6.94355
H	4.177108	2.895133	-7.49848
C	5.94677	1.133639	-6.36134
H	6.596879	1.097219	-7.24015
H	5.017248	0.629303	-6.62475
H	6.432699	0.540326	-5.58158

H	5.954566	1.312007	-3.54285
H	3.240611	3.905729	-5.6046
H	-1.23922	4.990238	-4.86101
H	-4.67271	5.596646	-2.36835
H	-6.12679	3.353455	1.459333
H	-5.52273	1.669294	5.352735
H	-1.8585	-1.01444	7.138331
H	2.300345	-1.96409	7.213186
H	3.525867	0.785458	-0.76813
H	-1.23565	-4.9598	10.10019
C	-0.65607	-4.05298	10.0051
C	0.796498	-1.70995	9.702338
C	0.04947	-3.54985	11.09462
C	-0.64857	-3.38947	8.770418
C	0.088699	-2.21343	8.602307
C	0.77698	-2.36908	10.93227
H	-1.20866	-3.81675	7.94212
H	1.368374	-1.9545	11.73927
H	1.387129	-0.80154	9.600856
C	0.451635	-4.60932	14.67189
C	0.406776	-6.13886	16.88104
C	0.910518	-4.07135	15.87332
N	0.014977	-5.80823	14.62127
C	-0.00937	-6.57886	15.62857
C	0.881594	-4.84756	17.01198
H	-0.39583	-7.57969	15.47975
H	1.212213	-4.44832	17.95746
H	0.345087	-6.80235	17.73236
N	0.033725	-4.19823	12.27058
C	0.430448	-3.75743	13.46976
H	0.748499	-2.71332	13.61894
Cu	-0.71075	-6.36509	12.62795
P	-3.09292	-6.69776	12.69838
P	0.488522	-8.16406	11.58295
C	-3.77144	-6.07749	14.35238
C	-4.7437	-5.12323	16.7921
C	-4.81221	-6.74501	14.99855
C	-3.23304	-4.92262	14.9247
C	-3.71135	-4.45062	16.14483
C	-5.29572	-6.26765	16.21791
H	-5.25599	-7.63331	14.56023
H	-2.43774	-4.38179	14.41976
H	-3.27607	-3.55841	16.58329
H	-6.10522	-6.78854	16.71682
H	-5.11914	-4.7499	17.73669

C	-3.84713	-5.69766	11.28647
C	-4.86598	-4.195	9.174906
C	-4.43259	-4.45455	11.53157
C	-3.77591	-6.18725	9.978984
C	-4.28573	-5.43598	8.923546
C	-4.94148	-3.70368	10.47542
H	-4.48238	-4.04876	12.53601
H	-3.31	-7.14654	9.772178
H	-4.21464	-5.81119	7.905559
H	-5.38133	-2.72906	10.66539
H	-5.25061	-3.6013	8.355817
C	-0.38966	-8.60833	9.975283
C	-1.72035	-9.17229	7.584994
C	-0.39577	-7.67519	8.93677
C	-1.03153	-9.83427	9.80464
C	-1.70267	-10.1124	8.613166
C	-1.06049	-7.9567	7.7453
H	0.120891	-6.72591	9.052052
H	-1.0224	-10.579	10.59238
H	-2.21471	-11.0616	8.485592
H	-1.07126	-7.22483	6.942806
H	-2.24505	-9.38312	6.656797
C	2.264715	-7.63844	11.17848
C	4.902122	-6.91784	10.61142
C	2.922881	-6.71061	11.98479
C	2.932538	-8.21093	10.09016
C	4.25048	-7.85276	9.809729
C	4.238146	-6.34688	11.69673
H	2.419186	-6.26084	12.83523
H	2.432745	-8.93707	9.454527
H	4.761205	-8.29396	8.958469
H	4.739528	-5.61166	12.31894
H	5.923986	-6.62818	10.38743
C	-3.53225	-8.53016	12.49599
C	-4.23655	-11.2074	12.11171
C	-2.69757	-9.54159	12.98168
C	-4.7269	-8.86626	11.84443
C	-5.07421	-10.2001	11.64732
C	-3.05801	-10.8805	12.78491
H	-5.39266	-8.0882	11.4817
H	-5.9949	-10.4507	11.12876
H	-2.4426	-11.6927	13.15177
H	-4.50329	-12.2482	11.95656
C	0.560919	-9.65419	12.74638
C	0.815566	-11.7547	14.58697

C	1.695685	-10.4734	12.73949
C	-0.46898	-9.93411	13.64604
C	-0.32184	-10.9508	14.59496
C	1.8177	-11.524	13.64942
H	2.50637	-10.2895	12.04131
H	-1.09705	-11.1191	15.3346
H	2.707553	-12.146	13.63876
H	0.924349	-12.5531	15.31266
O	-1.58817	-9.14197	13.69209
F	1.369087	-2.81403	15.94937
B	-0.19179	-0.06928	0.211225
F	-0.87005	0.5927	-0.59065
F	1.010986	0.084508	-0.03695
F	-0.47884	-1.24824	0.108437
F	-0.42444	0.306644	1.351587

Coordinates for ***meta*-Dimer**

Atom	x	y	z
C	-7.1919	-1.82799	3.150977
C	-5.67704	-1.17441	0.872793
C	-7.79054	-1.26797	2.009516
C	-5.79937	-2.03728	3.113424
C	-5.04073	-1.77304	1.967532
C	-7.05641	-0.93008	0.854946
H	-5.07004	-0.95263	-0.00456
C	-7.73763	-0.25561	-0.29476
C	-7.02955	0.423879	-1.2247
H	-5.96084	0.570296	-1.06367
C	-9.12956	-0.53581	-0.48336
N	-10.2581	-0.78628	-0.59492
C	-9.10384	2.3615	-3.80093
C	-6.5076	1.329128	-3.4458
C	-8.12633	2.471426	-4.80571
C	-8.73882	1.693075	-2.61831
C	-7.47235	1.124346	-2.44827
C	-6.81879	1.958687	-4.65843

H	-5.51268	0.91638	-3.28139
C	-5.80675	2.165871	-5.74596
C	-4.47773	2.045038	-5.5214
H	-4.13274	1.902456	-4.49699
C	-6.31046	2.266547	-7.08276
N	-6.75109	2.339047	-8.15424
C	-2.22577	3.023834	-8.47731
C	-2.13077	1.549947	-6.08409
C	-1.07293	2.309689	-8.10427
C	-3.34086	2.945854	-7.62049
C	-3.3338	2.168378	-6.45728
C	-0.99411	1.561253	-6.91136
H	-2.1281	0.979598	-5.15602
C	0.29523	0.888971	-6.52969
C	0.532469	0.507981	-5.25524
H	-0.19103	0.802123	-4.4943
C	1.184055	0.506132	-7.5842
N	1.886478	0.21593	-8.46216
C	3.998909	-0.92464	-4.42975
C	1.38713	-0.84041	-3.40768
C	3.646775	-1.5854	-3.23918
C	2.996063	-0.21643	-5.1089
C	1.676517	-0.19387	-4.62723
C	2.346378	-1.58503	-2.71578
H	0.363923	-0.80207	-3.03327
C	2.096624	-2.27593	-1.41529
C	1.021763	-1.98788	-0.65115
H	0.386492	-1.14944	-0.94058
C	2.903227	-3.43096	-1.15226
N	3.589886	-4.35745	-1.01949
C	0.963481	-3.63407	2.819057
C	-0.774	-2.43448	0.956287
C	-0.41922	-3.55208	3.055515
C	1.43734	-3.14132	1.586867
C	0.580431	-2.57977	0.63088
C	-1.31401	-2.96993	2.134003
H	-1.42205	-1.96156	0.218978
C	-2.76898	-2.86936	2.46867
C	-3.58639	-2.00593	1.827142
C	-3.30959	-3.87197	3.332035
N	-3.7348	-4.70011	4.024469
C	-2.29953	3.873687	-9.76118
C	-10.5185	2.954476	-3.94591
C	1.940802	-4.24201	3.844463
C	-8.00268	-2.17545	4.413899

C	3.053718	-3.23014	4.190186
H	2.628131	-2.32105	4.625762
H	3.642788	-2.93637	3.316211
H	3.756024	-3.64756	4.921338
C	2.579575	-5.51202	3.246186
H	1.813975	-6.25083	2.981541
H	3.264425	-5.98357	3.960698
H	3.156279	-5.29557	2.340114
C	1.279875	-4.64245	5.184494
H	0.522143	-5.42232	5.047
H	2.023602	-5.03777	5.886331
H	0.803205	-3.7838	5.672204
H	2.505543	-3.18181	1.393305
H	-0.81327	-3.92305	3.999041
C	-1.03264	3.790619	-10.6433
H	-0.14731	4.164168	-10.1158
H	-1.14215	4.39784	-11.55
H	-0.83169	2.761978	-10.9643
C	-2.49454	5.357596	-9.38715
H	-1.65212	5.721714	-8.78927
H	-3.40942	5.525319	-8.80837
H	-2.55946	5.986527	-10.2827
C	-3.48788	3.411014	-10.6327
H	-4.45225	3.563765	-10.1365
H	-3.40621	2.345397	-10.8771
H	-3.52605	3.968054	-11.5763
C	-11.5854	1.875338	-3.65303
H	-12.5971	2.267319	-3.8109
H	-11.5455	1.517822	-2.6187
H	-11.458	1.006478	-4.30939
C	-10.8228	3.511501	-5.35536
H	-11.8487	3.894421	-5.41593
H	-10.7183	2.737196	-6.12432
H	-10.159	4.343331	-5.61597
C	-10.686	4.115442	-2.94423
H	-11.6852	4.560155	-3.01698
H	-9.95602	4.908883	-3.13878
H	-10.5508	3.789705	-1.90681
C	-7.64049	-3.58792	4.926849
H	-8.26381	-3.87041	5.783609
H	-6.60118	-3.65496	5.263187
H	-7.78728	-4.3421	4.144824
C	-9.5342	-2.16531	4.198229
H	-10.0634	-2.47693	5.10695
H	-9.83029	-2.85075	3.395577

H	-9.90584	-1.16496	3.948591
C	-7.68007	-1.14067	5.506291
H	-8.23846	-1.3487	6.425912
H	-7.94586	-0.12873	5.182906
H	-6.61422	-1.13671	5.758809
H	-5.30738	-2.40171	4.009781
H	-8.85638	-1.05602	2.027748
H	-9.46303	1.64322	-1.81055
H	-8.37646	2.99938	-5.72241
H	-4.22331	3.526288	-7.87397
H	-0.19633	2.360506	-8.74437
H	3.269153	0.310302	-6.0186
H	4.445589	-2.08629	-2.68989
H	-3.14359	-1.28922	1.134199
H	8.073763	0.907909	-5.75557
C	7.527871	0.011293	-5.50137
C	6.065681	-2.24759	-4.8704
C	8.192963	-1.2084	-5.39759
C	6.149899	0.117441	-5.24029
C	5.400856	-1.0088	-4.8833
C	7.438932	-2.34498	-5.11166
H	5.688611	1.1012	-5.27829
H	7.893702	-3.32715	-5.06181
H	5.521824	-3.16114	-4.63491
C	11.80907	-2.25914	-5.52402
C	14.46234	-2.19394	-5.9687
C	12.57472	-3.38737	-5.22268
N	12.36124	-1.20714	-5.99534
C	13.60577	-1.12828	-6.23535
C	13.93623	-3.35933	-5.44324
H	13.97745	-0.19921	-6.65191
H	14.55435	-4.2171	-5.21272
H	15.52413	-2.1024	-6.17087
N	9.528469	-1.2786	-5.54485
C	10.35233	-2.30152	-5.28616
H	9.976163	-3.24655	-4.86179
C	u<br>10.871613	0.423198	-6.32038
P	10.54117	0.96994	-8.63637
P	11.36048	2.317876	-4.92719
C	11.32629	-0.34846	-9.73645
C	12.47184	-2.31984	-11.3527
C	11.90513	-0.00288	-10.9594
C	11.31502	-1.68508	-9.33101
C	11.88978	-2.66829	-10.1355

C	12.4777	-0.98798	-11.7653
H	11.91442	1.029619	-11.295
H	10.85398	-1.96781	-8.38747
H	11.88017	-3.70503	-9.80974
H	12.92745	-0.7164	-12.7164
H	12.91751	-3.08664	-11.9802
C	8.670034	1.024882	-8.91135
C	5.889123	1.080248	-9.18176
C	8.000175	-0.05881	-9.48155
C	7.94261	2.136699	-8.47614
C	6.555904	2.164804	-8.61514
C	6.611637	-0.03002	-9.61515
H	8.543082	-0.94254	-9.80251
H	8.447808	2.978413	-8.00987
H	5.996286	3.025655	-8.26337
H	6.088752	-0.88304	-10.0403
H	4.805286	1.087423	-9.27294
C	10.2025	3.747095	-5.34306
C	8.455228	5.839774	-5.97317
C	8.846337	3.650251	-5.02844
C	10.68308	4.910304	-5.94718
C	9.810677	5.950324	-6.27007
C	7.976599	4.692896	-5.34552
H	8.458651	2.774336	-4.5145
H	11.73848	5.023765	-6.17093
H	10.19092	6.848628	-6.74829
H	6.923526	4.613048	-5.09104
H	7.772267	6.649469	-6.21481
C	11.15474	1.828917	-3.10763
C	10.88391	1.084175	-0.42782
C	11.55256	0.558019	-2.68826
C	10.63448	2.732706	-2.17731
C	10.49537	2.357756	-0.84046
C	11.41356	0.185102	-1.3516
H	11.96665	-0.15107	-3.39923
H	10.32951	3.729984	-2.48248
H	10.07643	3.05708	-0.12158
H	11.71228	-0.81066	-1.03492
H	10.76754	0.789406	0.611571
C	11.30945	2.653039	-9.0412
C	12.36857	5.159486	-9.6733
C	12.4792	3.089568	-8.40913
C	10.68861	3.464991	-9.99973
C	11.21246	4.717764	-10.3084
C	13.00624	4.345196	-8.73456

H	9.79046	3.124631	-10.51
H	10.71807	5.346464	-11.0445
H	13.91675	4.714533	-8.27461
H	12.78101	6.135841	-9.91395
C	13.16132	2.835304	-5.21503
C	15.87662	3.453215	-5.53505
C	13.91018	3.317273	-4.13576
C	13.77083	2.714498	-6.46739
C	15.1355	2.980833	-6.61659
C	15.26047	3.631622	-4.29806
H	13.45394	3.431187	-3.15551
H	15.61832	2.821987	-7.57718
H	15.83595	3.994779	-3.44998
H	16.93415	3.673471	-5.65328
O	13.07828	2.207125	-7.53861
F	12.0177	-4.49959	-4.72027
C	1.945352	4.734108	-6.24784
C	0.231597	4.218605	-4.07826
C	0.644211	5.264956	-6.2012
C	2.353499	3.927307	-5.16728
C	1.537117	3.712397	-4.05094
C	-0.23622	5.02272	-5.12773
H	-0.40219	4.022256	-3.21429
C	-1.64103	5.540928	-5.17587
C	-2.60262	5.012457	-4.386
H	-2.36507	4.12647	-3.79699
C	-1.87818	6.734735	-5.93201
N	-2.03561	7.698805	-6.56063
C	-6.16393	6.292142	-4.99871
C	-4.64319	4.976835	-3.02667
C	-6.71871	5.942011	-3.75486
C	-4.81096	5.972744	-5.21289
C	-4.02672	5.370709	-4.224
C	-5.9794	5.287258	-2.74532
H	-4.03028	4.474315	-2.27904
C	-6.65104	4.885836	-1.46624
C	-6.12847	3.948849	-0.64084
H	-5.25123	3.390203	-0.96968
C	-7.74179	5.706577	-1.03295
N	-8.61601	6.398955	-0.71095
C	-8.37521	2.963511	2.316183
C	-5.68051	2.844992	1.518947
C	-7.38296	2.45066	3.170648
C	-7.9608	3.445481	1.059536
C	-6.617	3.4532	0.669352

C	-6.02385	2.377762	2.800561
H	-4.64404	2.81854	1.183582
C	-5.02367	1.750663	3.73162
C	-3.82434	1.322155	3.280258
H	-3.64693	1.347197	2.204351
C	-5.30923	1.772052	5.134487
N	-5.56704	1.791958	6.266832
C	-1.53322	-0.48791	5.776262
C	-1.43231	0.833292	3.303106
C	-0.32328	-0.31165	5.080579
C	-2.70457	0.022249	5.19539
C	-2.66472	0.717326	3.976517
C	-0.23569	0.364512	3.854297
H	-1.41635	1.362634	2.350133
C	1.089684	0.45495	3.169084
C	1.189203	0.714698	1.84755
H	0.274571	0.743995	1.253545
C	2.239642	0.510219	4.022461
N	3.120097	0.556161	4.777817
C	4.711278	0.417281	0.344928
C	2.211877	1.593596	-0.20158
C	4.511247	1.216267	-0.79335
C	3.623995	0.270229	1.229166
C	2.38625	0.883693	0.994261
C	3.275201	1.830015	-1.0839
H	1.232821	2.032546	-0.39016
C	3.120258	2.630787	-2.33874
C	1.906029	2.914252	-2.85961
C	4.289832	3.281837	-2.84055
N	5.24516	3.814567	-3.22699
C	-9.86646	3.007731	2.702689
C	-6.97824	6.983385	-6.10913
C	6.056278	-0.27671	0.638141
C	2.893213	4.98691	-7.43653
C	5.850215	-1.80088	0.763916
H	5.441329	-2.21537	-0.16313
H	5.166603	-2.06639	1.576111
H	6.798557	-2.3133	0.964177
C	6.635351	0.26841	1.960131
H	6.776932	1.354556	1.909992
H	7.608905	-0.18454	2.181266
H	5.981739	0.059939	2.814339
C	7.128121	-0.06407	-0.45726
H	7.379607	0.99587	-0.57834
H	6.794407	-0.4503	-1.42788

H	8.057913	-0.58846	-0.20636
H	3.753899	-0.36113	2.10379
H	5.332162	1.342412	-1.49585
C	-10.1577	2.520228	4.140356
H	-9.86574	1.472962	4.278894
H	-11.2281	2.585101	4.370073
H	-9.62894	3.124842	4.886255
C	-10.675	2.10679	1.746086
H	-10.3375	1.066998	1.812718
H	-10.5823	2.418973	0.700175
H	-11.7427	2.123659	1.993893
C	-10.3938	4.45589	2.601007
H	-10.3534	4.842621	1.577138
H	-9.80938	5.133755	3.234131
H	-11.4403	4.518448	2.9218
C	-6.25075	8.259143	-6.59088
H	-6.84845	8.797134	-7.33618
H	-5.28698	8.038145	-7.06171
H	-6.0627	8.944653	-5.75615
C	-8.39496	7.417708	-5.66875
H	-8.92189	7.930769	-6.48224
H	-8.35694	8.108867	-4.81863
H	-9.01315	6.559233	-5.38344
C	-7.14043	6.01477	-7.29825
H	-7.71288	6.477595	-8.11045
H	-7.67306	5.10708	-6.99399
H	-6.17534	5.707153	-7.71613
C	4.301187	5.387356	-6.94001
H	4.960183	5.641725	-7.77868
H	4.79407	4.577773	-6.39289
H	4.253987	6.258702	-6.27651
C	2.422057	6.116131	-8.38221
H	3.156571	6.299464	-9.17575
H	2.282222	7.059142	-7.84099
H	1.479332	5.865042	-8.88154
C	3.000909	3.696352	-8.26886
H	3.669531	3.832543	-9.12646
H	2.022834	3.396468	-8.65961
H	3.390654	2.861712	-7.67592
H	3.327304	3.450139	-5.21858
H	0.289511	5.858388	-7.03997
H	-4.37887	6.187934	-6.1856
H	-7.76828	6.157816	-3.57175
H	-8.72183	3.804298	0.37262
H	-7.67751	2.067261	4.143728

H	-3.64214	-0.1289	5.72215
H	0.573402	-0.7575	5.513193
H	1.027798	2.447229	-2.41314
H	-2.97068	-3.74245	8.790254
C	-2.2651	-2.94969	8.588821
C	-0.51856	-0.88322	8.021984
C	-1.23491	-2.68128	9.487198
C	-2.40123	-2.22209	7.392869
C	-1.50932	-1.1919	7.074023
C	-0.37745	-1.61965	9.202559
H	-3.19553	-2.50064	6.704404
H	0.421498	-1.34017	9.878653
H	0.180478	-0.06801	7.841764
C	-0.01857	-4.31603	12.66017
C	0.083431	-5.93161	14.81003
C	1.055334	-4.19988	13.54541
N	-0.93791	-5.17969	12.86522
C	-0.93987	-5.96202	13.86558
C	1.115254	-5.0262	14.64849
H	-1.77172	-6.65004	13.962
H	1.935435	-4.95754	15.35089
H	0.061809	-6.60859	15.65731
N	-1.06357	-3.44242	10.58385
C	-0.07692	-3.41858	11.48849
H	0.767327	-2.71654	11.398
C	u -<br>2.493028	-5.12067	11.27661
P	-4.62243	-4.38174	12.10829
P	-2.60114	-7.17229	10.03257
C	-4.41965	-3.63799	13.83164
C	-4.09591	-2.51145	16.36865
C	-5.4025	-3.81579	14.80721
C	-3.27874	-2.8885	14.12911
C	-3.11558	-2.32867	15.39544
C	-5.23925	-3.25347	16.07414
H	-6.29926	-4.38847	14.59156
H	-2.51308	-2.73328	13.37222
H	-2.22335	-1.74946	15.61779
H	-6.00558	-3.39287	16.83168
H	-3.96948	-2.07303	17.35459
C	-5.24096	-3.06993	10.89584
C	-6.03415	-1.15914	9.019155
C	-5.08414	-1.71058	11.17124
C	-5.79667	-3.46942	9.677313
C	-6.19522	-2.51544	8.743422

C	-5.47994	-0.75759	10.23277
H	-4.63157	-1.37843	12.10033
H	-5.90525	-4.52424	9.439724
H	-6.61371	-2.83419	7.794275
H	-5.33814	0.300434	10.43818
H	-6.32345	-0.41011	8.285837
C	-4.08329	-7.15044	8.862852
C	-6.274	-7.08031	7.123399
C	-4.0796	-6.29332	7.761434
C	-5.17541	-7.99552	9.068274
C	-6.27261	-7.9537	8.20722
C	-5.17375	-6.25735	6.897528
H	-3.21885	-5.66289	7.553637
H	-5.18592	-8.69724	9.895346
H	-7.12332	-8.60704	8.380051
H	-5.16229	-5.59156	6.039123
H	-7.12195	-7.04882	6.444719
C	-1.01195	-7.38761	9.024289
C	1.33805	-7.6929	7.54424
C	0.198742	-6.90581	9.524033
C	-1.0411	-8.03492	7.785437
C	0.1324	-8.1836	7.045906
C	1.37084	-7.0543	8.782754
H	0.23703	-6.40214	10.48571
H	-1.97485	-8.41941	7.383544
H	0.104479	-8.67319	6.07601
H	2.307148	-6.66061	9.169267
H	2.25013	-7.7978	6.963002
C	-5.84513	-5.82708	12.19042
C	-7.70834	-7.90831	12.25829
C	-5.41941	-7.12874	12.48064
C	-7.2032	-5.5729	11.95556
C	-8.13003	-6.61205	11.98088
C	-6.36014	-8.16522	12.51765
H	-7.54529	-4.56175	11.74693
H	-9.17948	-6.40837	11.78374
H	-6.07115	-9.18519	12.74812
H	-8.42975	-8.72098	12.2801
C	-2.7625	-8.61022	11.25441
C	-2.82894	-10.7166	13.10201
C	-2.12004	-9.82047	10.97184
C	-3.48098	-8.48155	12.44737
C	-3.47733	-9.51638	13.38777
C	-2.16028	-10.8716	11.88933
H	-1.56569	-9.94758	10.04546

H	-3.98138	-9.38589	14.34166
H	-1.64854	-11.8043	11.66429
H	-2.83615	-11.5263	13.82667
O	-4.08521	-7.29321	12.77735
F	2.031434	-3.30048	13.35005
B	-2.37777	1.229372	-1.2195
F	-3.09007	1.875978	-1.99825
F	-1.22863	1.14571	-1.68074
F	-2.84137	0.111975	-1.07771
F	-2.34286	1.792093	-0.13557

26. X-Ray Crystallography

Single crystals of gold(I) model complex suitable for X-ray diffraction were grown by vapor diffusion of diethyl ether into a dichloromethane solution of the complex. A yellow crystal (Figure S65, block, approximate dimensions 0.15 × 0.14 × 0.12 mm^3) was placed onto the tip of a MiTeGen pin and mounted on a Bruker Venture D8 diffractometer equipped with a PhotonIII detector at 100.0 K.

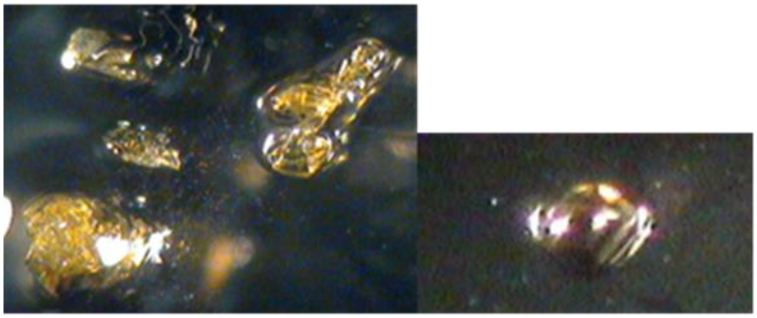

Figure S80. Microscope images of bulk material and crystal selected.

Data collection

The data collection was carried out using Mo Kα radiation (λ = 0.71073 Å, IμS micro-source) with a frame time of 2 seconds and a detector distance of 40 mm. A collection strategy was calculated and complete data to a resolution of 0.70 Å with a redundancy of 4.5 were collected. The total exposure time was 1.36 hours. The frames were integrated with the Bruker SAINT[17] software package using a narrow-frame algorithm. The integration of the data using a triclinic unit cell yielded a total of 88532 reflections to a maximum θ angle of 30.52° (0.70 Å resolution), of which 10365 were independent (average redundancy 8.541, completeness = 99.7%, R_{int} = 3.16%, R_{sig} = 1.70%) and 9867 (95.20%) were greater than $2\sigma(F^2)$. The final cell constants of a = 8.6133(4) Å, b = 14.6051(6) Å, c = 15.5136(8) Å, α = 62.663(2)°, β = 79.227(2)°, γ = 82.934(2)°, volume = 1701.53(14) $Å^3$, are based upon the refinement of the XYZ-centroids of 9286 reflections above 20 σ(I) with 5.298° < 2θ < 60.97°. Data were corrected for absorption effects using the Multi-Scan method (SADABS).[18] The ratio of minimum to maximum apparent transmission was 0.862. The calculated minimum and maximum transmission coefficients (based on crystal size) are 0.5470

and 0.6100. Please refer to Table S10 for additional crystal and refinement information.

Structure solution and refinement

The space group P-1 was determined based on intensity statistics and systematic absences. The structure was solved using the SHELX suite of programs[19, 20] and refined using full-matrix least-squares on F^2 within the OLEX2 suite.[21] An intrinsic phasing solution was calculated, which provided most non-hydrogen atoms from the E-map. Full-matrix least squares / difference Fourier cycles were performed, which located the remaining non-hydrogen atoms. All non-hydrogen atoms were refined with anisotropic displacement parameters. The hydrogen atoms were placed in ideal positions and refined as riding atoms with relative isotropic displacement parameters. The final full matrix least squares refinement converged to R1 = 0.0213 and wR2 = 0.0514 (F^2, all data). The goodness-of-fit was 1.030. On the basis of the final model, the calculated density was 1.861 g/cm^3 and F(000), 932 e$^-$.

Structure description (CCDC Deposition Number 2091810)

Asymmetric unit ($CF_3SO_2N^-$ molecules have 0.5 occupancy each and are disordered over a special position)

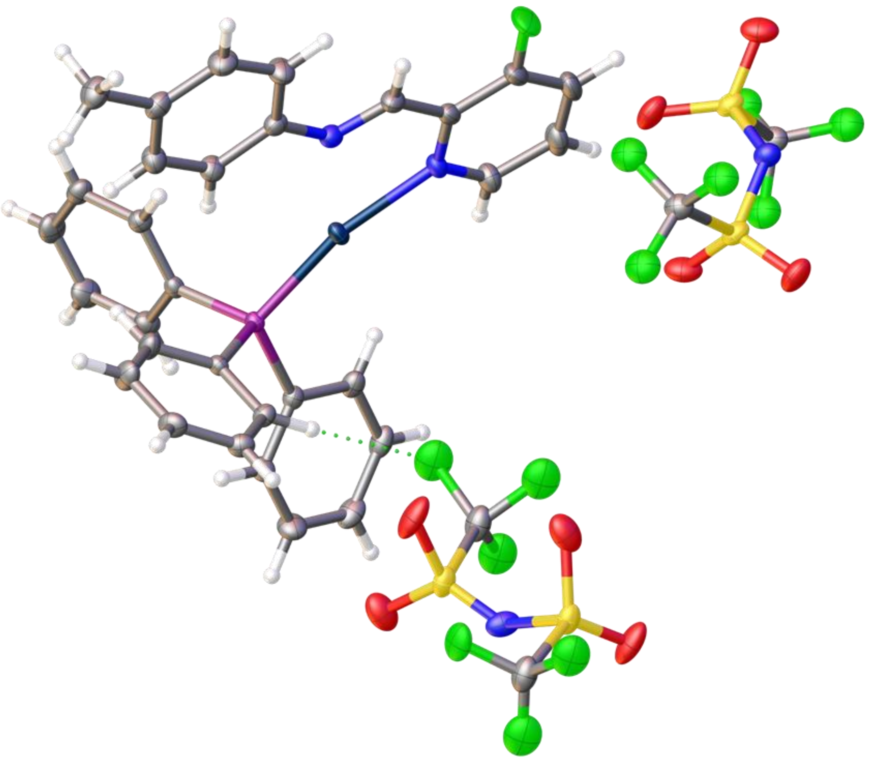

Table S10. Crystal data and structure refinement.

Empirical formula	C33 H26 Au F7 N3 O4 P S2	
Formula weight	953.62	
Crystal color, shape, size	yellow block, 0.15 × 0.14 × 0.12 mm^3	
Temperature	100.0 K	
Wavelength	0.71073 Å	
Crystal system, space group	Triclinic, P-1	
Unit cell dimensions	a = 8.6133(4) Å	α = 62.663(2)°.
	b = 14.6051(6) Å	β = 79.227(2)°.
	c = 15.5136(8) Å	γ = 82.934(2)°.
Volume	1701.53(14) $Å^3$	
Z	2	
Density (calculated)	1.861 g/cm^3	
Absorption coefficient	4.575 mm^{-1}	
F(000)	932	
**Data collection**		
Diffractometer	Bruker VENTURE D8	
Theta range for data collection	2.409 to 30.516°.	
Index ranges	-12<=h<=12, -20<=k<=20, -22<=l<=22	
Reflections collected	88532	
Independent reflections	10365 [R_{int} = 0.0316]	
Observed Reflections	9867	
Completeness to theta = 25.242°	99.8 %	
**Solution and Refinement**		
Absorption correction	Semi-empirical from equivalents	
Max. and min. transmission	0.7461 and 0.6433	
Solution	Intrinsic methods	
Refinement method	Full-matrix least-squares on F^2	
Weighting scheme	w = $[\sigma^2Fo^2+ AP^2+ BP]^{-1}$, with	
	P = $(Fo^2+ 2 Fc^2)/3$, A = 0.0216, B = 2.8537	
Data / restraints / parameters	10365 / 537 / 494	
Goodness-of-fit on F^2	1.030	
Final R indices [I>2σ(I)]	R1 = 0.0213, wR2 = 0.0506	
R indices (all data)	R1 = 0.0229, wR2 = 0.0514	
Extinction coefficient	n/a	
Largest diff. peak and hole	1.454 and -1.086 e.$Å^{-3}$	

27. References

(1) Dhara, A.; Sadhukhan, T.; Sheetz, E. G.; Olsson, A. H.; Raghavachari, K.; Flood, A. H. Zero-Overlap Fluorophores for Fluorescent Studies at Any Concentration. *J. Am. Chem. Soc.* **2020**, *28*, 12167-12180.

(2) Rosenau, C. P.; Jelier, B. J.; Gossert, A. D.; Togni, A. Exposing the Origins of Irreproducibility in Fluorine NMR Spectroscopy. *Angewandte Chemie International Edition* **2018**, *30*, 9528-9533.

(3) Yakelis, N. A.; Bergman, R. G. Safe Preparation and Purification of Sodium Tetrakis[(3,5-trifluoromethyl)phenyl]borate (NaBArF24): Reliable and Sensitive Analysis of Water in Solutions of Fluorinated Tetraarylborates. *Organometallics* **2005**, *14*, 3579-3581.

(4) Carreras, L.; Rovira, L.; Vaquero, M.; Mon, I.; Martin, E.; Benet-Buchholz, J.; Vidal-Ferran, A. Syntheses, characterisation and solid-state study of alkali and ammonium BArF salts. *RSC Adv.* **2017**, *52*, 32833-32841, 10.1039/C7RA05928K.

(5) Fatila, E. M.; Twum, E. B.; Sengupta, A.; Pink, M.; Karty, J. A.; Raghavachari, K.; Flood, A. H. Anions Stabilize Each Other inside Macrocyclic Hosts. *Angew. Chem. Int. Ed.* **2016**, *45*, 14057-14062.

(6) Fatila, E. M.; Pink, M.; Twum, E. B.; Karty, J. A.; Flood, A. H. Phosphate-phosphate oligomerization drives higher order co-assemblies with stacks of cyanostar macrocycles. *Chem. Sci.* **2018**, *11*, 2863-2872.

(7) Fatila, E. M.; Twum, E. B.; Karty, J. A.; Flood, A. H. Ion Pairing and Co-facial Stacking Drive High-Fidelity Bisulfate Assembly with Cyanostar Macrocyclic Hosts. *Chem. Eur. J.* **2017**, *44*, 10652-10662.

(8) Zhao, W.; Qiao, B.; Tropp, J.; Pink, M.; Azoulay, J. D.; Flood, A. H. Linear Supramolecular Polymers Driven by Anion-Anion Dimerization of Difunctional Phosphonate Monomers Inside Cyanostar Macrocycles. *J. Am. Chem. Soc.* **2019**, *12*, 4980-4989.

(9) Zhao, W.; Tropp, J.; Qiao, B.; Pink, M.; Azoulay, J. D.; Flood, A. H. Tunable Adhesion from Stoichiometry-Controlled and Sequence-Defined Supramolecular Polymers Emerges Hierarchically from Cyanostar-Stabilized Anion–Anion Linkages. *Journal of the American Chemical Society* **2020**, *5*, 2579-2591.

(10) Luong, L. M. C.; Aristov, M. M.; Adams, A. V.; Walters, D. T.; Berry, J. F.; Olmstead, M. M.; Balch, A. L. Unsymmetrical Coordination of Bipyridine in Three-Coordinate Gold(I) Complexes. *Inorg. Chem.* **2020**, *6*, 4109-4117.

(11) Rivada-Wheelaghan, O.; Aristizabal, S. L.; Lopez-Serrano, J.; Fayzullin, R. R.; Khusnutdinova, J. R. Controlled and Reversible Stepwise Growth of Linear Copper(I) Chains Enabled by Dynamic Ligand Scaffolds. *Angew. Chem. Int. Ed.* **2017**, *51*, 16267-16271.

(12) Asil, D.; Foster, J. A.; Patra, A.; de Hatten, X.; del Barrio, J.; Scherman, O. A.; Nitschke, J. R.; Friend, R. H. Temperature- and Voltage-Induced Ligand Rearrangement of a Dynamic Electroluminescent Metallopolymer. *Angewandte Chemie International Edition* **2014**, *32*, 8388-8391.

(13) Asil, D.; Foster, J. A.; Patra, A.; de Hatten, X.; del Barrio, J.; Scherman, O. A.; Nitschke, J. R.; Friend, R. H. Temperature- and Voltage-Induced Ligand Rearrangement of a Dynamic Electroluminescent Metallopolymer. *Angew. Chem. Int. Ed.* **2014**, *32*, 8388-8391.

(14) Lee, S.; Chen, C. H.; Flood, A. H. A pentagonal cyanostar macrocycle with cyanostilbene CH donors binds anions and forms dialkylphosphate [3]rotaxanes. *Nat. Chem.* **2013**, *8*, 704-710.

(15) Fadler, R. E.; Al Ouahabi, A.; Qiao, B.; Carta, V.; König, N. F.; Gao, X.; Zhao, W.; Zhang, Y.; Lutz, J.-F.; Flood, A. H. Chain Entropy Beats Hydrogen Bonds to Unfold and Thread Dialcohol Phosphates inside Cyanostar Macrocycles To Form [3]Pseudorotaxanes. *The Journal of Organic Chemistry* **2021**, *6*, 4532-4546.

(16) Fadler, R. E.; Al Ouahabi, A.; Qiao, B.; Carta, V.; Konig, N. F.; Gao, X.; Zhao, W.; Zhang, Y.; Lutz, J. F.; Flood, A. H. Chain Entropy Beats Hydrogen Bonds to Unfold and Thread Dialcohol Phosphates inside Cyanostar Macrocycles To Form [3]Pseudorotaxanes. *J. Org. Chem.* **2021**, *6*, 4532-4546.

(17) SAINT, Bruker Analytical X-Ray Systems, Madison, WI, current version.

(18) SADABS, Bruker Analytical X-Ray Systems, Madison, WI, current version.

(19) Sheldrick, G. SHELXT - Integrated space-group and crystal-structure determination. *Acta Crystallographica Section A* **2015**, *1*, 3-8.

(20) Sheldrick, G. A short history of SHELX. *Acta Cryst. A* **2008**, *1*, 112-122.

(21) Dolomanov, O. V.; Bourhis, L. J.; Gildea, R. J.; Howard, J. A. K.; Puschmann, H. OLEX2: a complete structure solution, refinement and analysis program. *J. Appl. Crystallogr.* **2009**, *2*, 339-341.
